# Comparison of high-titer lactic acid fermentation from NaOH- and NH_3_-H_2_O_2_-pretreated corncob by *Bacillus coagulans* using simultaneous saccharification and fermentation

**DOI:** 10.1038/srep37245

**Published:** 2016-11-17

**Authors:** Zhenting Zhang, Yuejiao Xie, Xiaolan He, Xinli Li, Jinlong Hu, Zhiyong Ruan, Shumiao Zhao, Nan Peng, Yunxiang Liang

**Affiliations:** 1 State Key Laboratory of Agricultural Microbiology, College of Life Science and Technology, Huazhong Agricultural University, Wuhan, 430070, Hubei, P.R. China; 2 Key Laboratory of Microbial Resources (Ministry of Agriculture, China), Institute of Agricultural Resources and Regional Planning, CAAS, Beijing, 100081, P.R. China; 3 Hubei Collaborative Innovation Center for Industrial Fermentation, Wuhan, 430068, P. R. China; 4 Key Laboratory of Development and Application of Rural Renewable Energy (Ministry of Agriculture), Biomass Energy Technology Research Centre, Biogas Institute of Ministry of Agriculture, Chengdu, 610041, Sichuan, P. R. China

## Abstract

Lignocellulose is one of the most abundant renewable feedstocks that has attracted considerable attention as a substrate for biofuel and biochemical production. One such biochemical product, lactic acid, is an important fermentation product because of its great potential for the production of biodegradable and biocompatible polylactic acid. High-titer lactic acid production from lignocellulosic materials has been achieved recently; however, it requires biodetoxification or results in large amounts of waste washing water. In this study, we employed two alkaline pretreatment methods and compared their effects on lactic acid fermentation of pretreated corncob by *Bacillus coagulans* LA204 using fed-batch simultaneous saccharification and fermentation under non-sterile conditions. The lactic acid titer, yield, and productivity from 16% (w/w) NaOH-pretreated and washed corncob were 122.99 g/L, 0.77 g/g corncob, and 1.37 g/L/h, respectively, and from 16% NH_3_-H_2_O_2_-pretreated and washed corncob were 118.60 g/L, 0.74 g/g corncob, and 1.32 g/L/h, respectively. Importantly, the lactic acid titer, yield, and productivity from 18.4% NH_3_-H_2_O_2_-pretreated and unwashed corncob by using fed-batch simultaneous saccharification and fermentation reached 79.47 g/L, 0.43 g/g corncob, and 1.10 g/L/h, respectively, demonstrating that this method is possible for industrial applications and saves washing water.

Lignocellulose, the most abundant global source of biomass, has been largely unutilized for biofuel and biochemical production. Over 800 million tons of lignocellulose have been produced in China since 2008, with approximately 505.5 million tons of primary biomass being available for further utilization^1^. Corncob is one of the most important agricultural residues available in high quantities, with 3.2 to 3.6 million tons produced in 2012 in China^2^. Additionally, corncob possesses great potential value as a raw material for the production of high value added chemicals, fuels, and other industrial products because of its high cellulose and hemicellulose content and high energy density^3^,^4^. However, effective utilization of lignocellulosic feedstock is not always practical because of its seasonal availability, scattered location, and prohibitive transportation and storage costs^5^. Currently, open-field burning of agricultural residues has become the preferred route of disposal; farmers in developing countries, especially in Asia, ignore the potential environmental effects and are unaware of the significance of crop residue returning in the field^6^. Thus, agro residue burning is widely regarded as a main source of toxic air pollutants, with both short- and long-term effects on human health, and fueling global climate changes^7^,^8^.

Lactic acid (LA) is an important biochemical product that has attracted increasing attention because of its widespread application in the food, chemical, cosmetic, and pharmaceutical industries. Furthermore, LA has great potential for the production of biodegradable and biocompatible polylactic acid (PLA) polymers, which has driven the current market development for LA. Fermentative production is the main route for producing LA; the advantages of this method include low production temperature and energy consumption, production of optically pure D- or l-lactic acid (l-LA), and cheap renewable substrates such as lignocellulosic biomass^9^.

The crystalline structure of lignocellulosic biomass results in two major technical obstacles to LA production: biomass pretreatment and hydrolysis and efficient fermentation of pentose from lignocellulosic hydrolysates. The pretreatments applied to agro residues include physical (size reduction), physicochemical (liquid hot water, steam explosion, and ammonia fiber explosion), chemical (acid, alkaline, alkaline/oxidative, wet oxidation, and ozonolysis), and biological methods^10^. Acid hydrolysis and steam can be used to hydrolyze hemicellulose into fermentable mono- or oligosaccharides using high temperature or pressure^11^; and alkaline treatments (lime, sodium hydroxide, wet-oxidation, and soaking with ammonia) provide efficient delignification, resulting in solid residues of cellulose fibers and certain hemicelluloses^12^,^13^,^14^,^15^,^16^. A method combining sodium hydroxide (NaOH) pre-extraction and alkaline hydrogen peroxide (H_2_O_2_) post-treatment was investigated using corn stover as the substrate. It was found that NaOH first solubilized and removed the easily-extracted lignin and xylan and the oxidizing post-treatment then removed the more recalcitrant lignin from the cell walls^17^. This combined approach achieved high enzymatic sugar yields from pretreated corn stover using low oxidant loading.

However, pretreatments generate inhibitors (phenolic compounds and formic acid in alkaline-pretreated biomass and hydroxymethyl furfural [HMF] and furfural in acid-pretreated biomass) that repress LA fermentation. Thus, efficient LA production from pretreated biomass requires the removal of these inhibitors prior to fermentation or the use of inhibitor-tolerant bacteria. Moreover, calcium carbonate (CaCO_3_) or NaOH are required to maintain the neutral or mildly acidic conditions favorable for LA fermentation. The resulting accumulation of sodium lactate or calcium lactate in the fermentation broth can have various stress effects on lactic acid bacteria^18^, however, like in other fermentation systems, removal of toxic products would improve fermentation yields^19^. Recently, several lactic acid bacteria, including *Lactobacillus* strains and *Bacillus coagulans*, have been reported to produce high-titer LA from lignocellulosic materials. *B. coagulans* strains, possessing robust inhibitor tolerance, were shown to be suitable for lignocellulosic LA production and were engineered for ethanol production because of their thermophilic growth characteristics and strong pentose homofermentative activity^16^,^20^. The LA yield and titer obtained with *B. coagulans* DSM 2314 reached 0.26 g/g lime-pretreated wheat stover and 40.7 g/L, respectively^21^. In addition, it has been reported that LA production yield and titer using oil palm empty fruit bunch acid hydrolysate with *B. coagulans* reached 0.97 g/g and 59.2 g/L, respectively^22^. The LA yield and titer obtained from acid-pretreated wheat stover via simultaneous saccharification and fermentation (SSF) using *B. coagulans* IPE22 reached 0.46 g/g acid-pretreated wheat stover and 38.73 g/L, respectively^23^. Interestingly, the LA yield and titer using *B. coagulans* LA204 reached 0.68 g/g substrate and 97.6 g/L, respectively, for fed-batch fermentation with 14.4% solid content using NaOH-pretreated and washed corn stover^12^. However, the major disadvantage of these studies is the substantial volume of waste washing water generated by inhibitor removal. In terms of industrial production, wastewater should be strictly limited because of the high cost of wastewater treatment^24^. In contrast, significant LA production was obtained from sulfuric acid-pretreated and biodetoxified corn stover by *Pediococcus acidilactici* DQ2. The LA titer reached 101.9 g/L, however, the yield only reached 0.38 g/g stover and *P. acidilactici* DQ2 cannot utilize xylose^25^. A high titer (104.4 g/L) of l-LA was obtained from dilute acid-pretreated and biodetoxified corn stover with <30% solid content using an engineered *P. acidilactici* TY112 (CGMCC 8664) strain. The yield reached 0.72 g/g glucose from total corn stover without considering xylose unavailability^24^.

In order to improve lignocellulosic LA production from both cellulose and hemicellulose hydrolysates and reduce the volume of washing water, we compared LA fermentation efficiency using NaOH-pretreated and ammonium-hydrogen peroxide (NH_3_-H_2_O_2_)-pretreated corncob via SSF with strain *B. coagulans* LA204. The LA yield, titer, and productivity reached 0.43 g/g corncob, 79.47 g/L, and 1.10 g/L/h, respectively, using NH_3_-H_2_O_2_-pretreated and unwashed corncob. This study provides a useful industrial application to avoid the generation of waste washing water.

## Results and Discussion

### Effect of NaOH and NH_3_-H_2_O_2_ pretreatments on corncob solid composition

Acid and alkaline pretreatments are commonly used to remove lignin from lignocellulosic materials. However, acid pretreatment results in loss of hemicellulose, while alkaline pretreatment maintains most of the hemicelluloses in the solid content and is thus more feasible for biochemical or biofuel fermentation using SSF^26^. In this study, we selected NaOH and NH_3_-H_2_O_2_ pretreatments to remove the lignin from the corncob and to render the cellulose and hemicellulose accessible to cellulase and hemicellulase. The determined compositional changes in the corncob prior to and post alkaline pretreatment are summarized in Table 1. Following NaOH pretreatment and washing, the cellulosic fraction (as glucose) increased significantly from 37.26% to 59.84%, the hemicellulose fraction (as xylose) decreased from 29.05% to 19.99%, and the lignin content decreased from 19.60% to 6.28%, compared to raw material without pretreatment (Table 1). Subsequent to this pretreatment, 91.71% cellulose, 39.29% hemicellulose, and 18.30% lignin were recovered. These results are in agreement with those of a previous report showing that dilute alkali pretreatment partially solubilizes hemicellulose and leads to swelling as well as disruption of the lignin structure^27^. The ash content remained constant following NaOH pretreatment and washing. However, subsequent to NH_3_-H_2_O_2_ pretreatment and washing the solid fraction exhibited a 19.54% increase in cellulosic composition and the percentage of hemicellulose, lignin, and ash decreased slightly compared to raw material prior to pretreatment (Table 1). Subsequent to 1-day NH_3_ pretreatment and 7-days H_2_O_2_ pretreatment followed by water washing, 94.43% cellulose, 71.28% hemicellulose, and 68.92% lignin were recovered. While 1-day and 7-days NH_3_-H_2_O_2_ pretreatment resulted in a 4.46% and 13.10% increase in cellulosic fraction, respectively, compared with the raw material, the hemicellulose composition was unchanged; this may be due to the remaining solubilized xylose in the pretreated corncob (Table 1). The lignin content remained the same following the 1-day H_2_O_2_ pretreatment but decreased from 19.60% to 16.61% after the 7-day H_2_O_2_ pretreatment. The ash content was unaffected by both pretreatments (Table 1). Subsequent to 1-day NH_3_ pretreatment and 1-day H_2_O_2_ pretreatment, 100.02% cellulose, 95.97% hemicellulose, and 93.68% lignin were recovered. After 1-day NH_3_ pretreatment and 7-days H_2_O_2_ pretreatment, 105.03% cellulose, 97.73% hemicellulose, and 82.62% lignin were recovered. These results demonstrate that NaOH pretreatment with subsequent washing can efficiently remove lignin; however, this pretreatment also solubilizes the hemicellulose fraction, resulting in a loss of oligosaccharides. In contrast, the NH_3_-H_2_O_2_ pretreatment preserves both the hemicellulose fraction and the lignin. Although lignin compounds were detected using the Folin-Ciocalteu method in the NH_3_-H_2_O_2_-pretreated corncob, their structures and characteristics may have changed because the presence of lignin compounds did not hinder LA fermentation when NH_3_-H_2_O_2_ pretreated and washed corncob was used. In addition, NH_3_-H_2_O_2_ pretreated and washed corncob had a smaller inhibitory effect than NH_3_-H_2_O_2_ pretreated and unwashed corncob (see below).

### High-titer and high-yield LA fermentation from NaOH-pretreated and washed corncob

**Figure 1:**
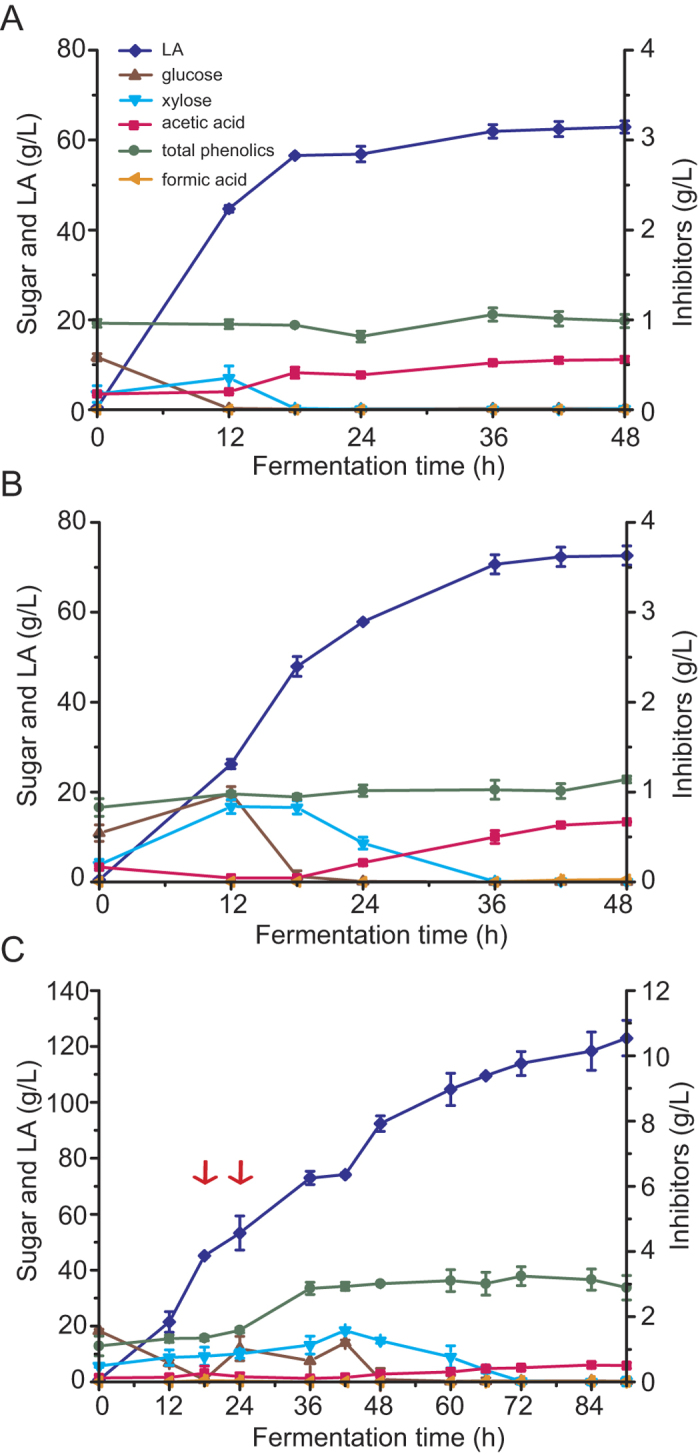
LA fermentation with NaOH-pretreated and washed corncob. (**A**) Concentration of sugars (glucose and xylose), LA, and inhibitors (acetic acid, total phenolics, and formic acid) during fermentation of 8% (w/w) NaOH-pretreated and washed corncob at pH6.0 (adjusted by automatic NaOH solution feeding). (**B**) Fermentation of 8% (w/w) NaOH-pretreated and washed corncob using CaCO_3_ as a neutralizer. (**C**) Fermentation of NaOH-pretreated and washed corncob (8% [w/w] initial substrate fed to 16% from 18 h to 24 h) using CaCO_3_ as aneutralizer. Blue diamond, LA; red square, acetic acid; green circle, total phenolics; brown triangle, glucose; cyan triangle, xylose; orange triangle, formic acid. Experiments were carried out in duplicate and error bars are shown. Where error bars are not visible, they are smaller than the size of the symbol used.

In our previous study, *B. coagulans* LA204 demonstrated remarkably efficient lignocellulosic LA production, with high LA yield and titer and low byproduct generation^12^. Moreover, LA titer and yield were increased when NaOH-pretreated and washed corncob was used compared with non-pretreated corncob^12^. In this study, NaOH and NH_3_-H_2_O_2_ were used to pretreat corncob, one of the most abundant agro biomasses in the world, and the LA fermentation ability of *B. coagulans* LA204 using these materials was compared. The use of 8% NaOH-pretreated and washed corncob as the carbon source, 10 g/L yeast extract as the nitrogen source, and 10 M NaOH solution as the neutralizer resulted in an LA yield of 0.79 g/g total corncob and an LA titer of 62.91 g/L. LA was produced rapidly initially and LA fermentation was nearly complete by 18 h (Fig. 1A); the LA titer reached 56.88 g/L at 18 h and the productivity during this period reached 3.16 g/L/h. Using the same fermentation conditions, with CaCO_3_ as the neutralizer, the LA yield and titer reached 0.91 g/g total corncob and 72.62 g/L, respectively. During the initial stage of fermentation (0 to 18 h) with CaCO_3_ as the neutralizer, LA productivity was 2.66 g/L/h, which was slightly lower than that obtained when NaOH was used as the neutralizer; however, LA was continuously produced during the period from 18 to 36 h with a productivity of 1.26 g/L/h (Fig. 1B). These results demonstrate that during the initial stage of fermentation with a lower LA titer, LA was produced more rapidly when NaOH was used as the neutralizer; however, with increasing amounts of LA, soluble sodium lactate had a stronger inhibitory effect on *B. coagulans* than calcium lactate, which has been previously examined at the transcriptome level^18^. Finally, a significantly higher amount of LA (0.91 g/g vs. 0.79 g/g corncob) was produced from 8% NaOH-pretreated and washed corncob using CaCO_3_ as the neutralizer compared to using NaOH as the neutralizer, as demonstrated by independent samples t-test (*p* < 0.05). Because CaCO_3_ was sufficient for enhancing LA yield, we performed fed-batch fermentation for high-titer LA production from corncob using CaCO_3_ as the neutralizer. A similar fermentation curve as shown in Fig. 1B was obtained during the initial stage (0 to 18 h) using 8% NaOH-pretreated and washed corncob and the LA titer reached 45.14 g/L (Fig. 1C). Corncob was then fed to 16% (w/w) and cellulase and yeast extract were fed from 18 to 24 h to maintain the concentrations at 30 filter paper unit (FPU)/g corncob and 10 g/L, respectively. Fermentation was continued for 90 h; the final LA titer and yield were 122.99 g/L and 0.77 g/g, respectively, and the overall productivity was 1.37 g/L/h (Fig. 1C, Table 2).The l-LA optical purity was 98%. These impressive results represent one of the highest levels of LA production from agro biomass reported to date. However, the key issue of efficient inhibitor removal without washing water requires resolution prior to feasible industrial application. Previously, we reported that *B. coagulans* is sensitive to inhibitors in corn stover created by NaOH pretreatment and that removal of the inhibitors by simple washing enhanced LA yield, titer, and productivity^12^. However, washing generates a large volume of wastewater that may hinder application; thus, a feasible pretreatment method needs to be developed to avoid the generation of wastewater.

### LA production from NH_3_-H_2_O_2_-pretreated corncob

**Figure 2:**
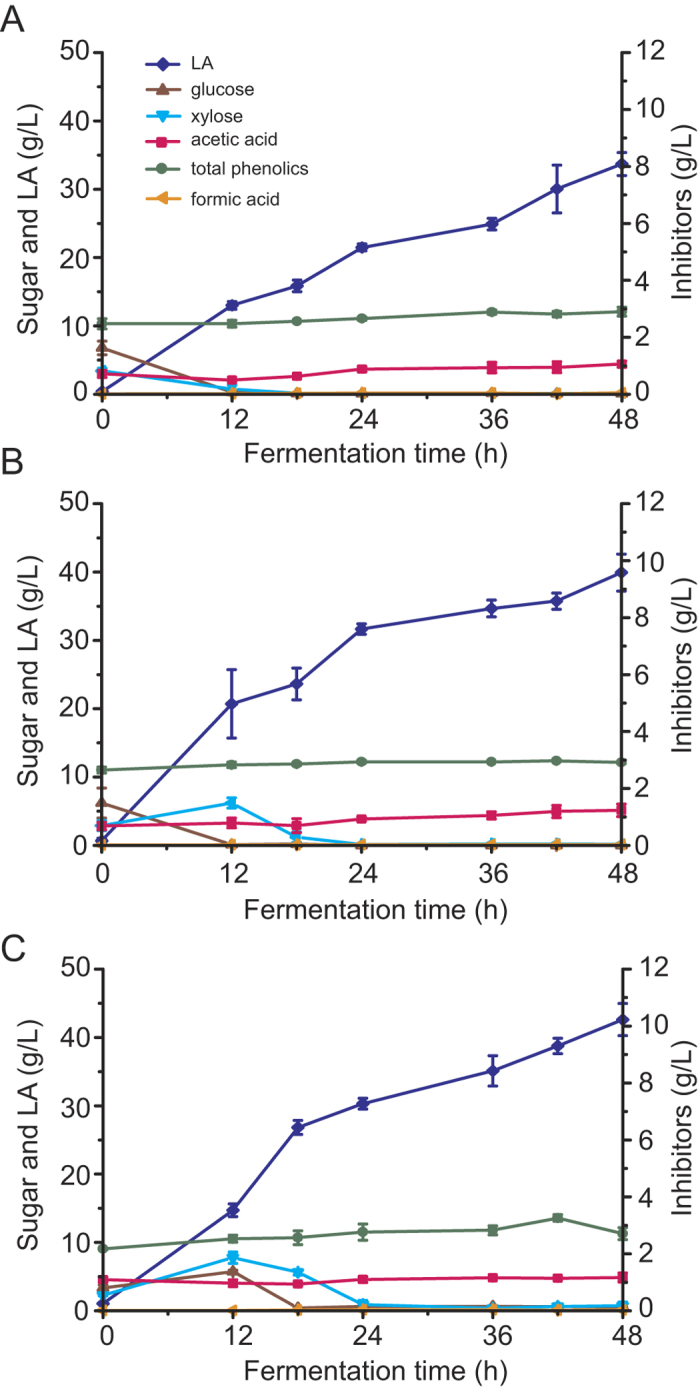
LA fermentation of 8% (w/w) NH_3_-H_2_O_2_-pretreated and unwashed corncob. (**A**) Concentration of sugars (glucose and xylose), LA, and inhibitors (acetic acid, total phenolics, and formic acid) during the fermentation of corncob substrate pretreated for 1 day with NH_3_ and then treated for 1 day with H_2_O_2_ at pH6.0 (adjusted by automatic NaOH solution feeding). (**B**) Fermentation of corncob substrate pretreated for 1 day with NH_3_ followed by 7 days of H_2_O_2_ treatment at pH6.0 (adjusted by automatic NaOH solution feeding). (**C**) Fermentation of corncob substrate pretreated for 1 day with NH_3_ then treated for 7 days with H_2_O_2_ using CaCO_3_ as a neutralizer. Blue diamond, LA; red square, acetic acid; green circle, total phenolics; brown triangle, glucose; cyan triangle, xylose; orange triangle, formic acid. Experiments were carried out in duplicate and error bars are shown. Where error bars are not visible, they are smaller than the size of the symbol used.

The LA yield and titer obtained from alkaline-pretreated corn stover via SSF using *B. coagulans* reached relative high titer and yield^12^. However, the major disadvantage is the substantial volume of waste washing water generated by inhibitor removal. In the above study, a large volume of water was used to wash the pretreated corn stover in order to remove phenolic inhibitors and to neutralize the pH value. The waste washing water contains large amounts of inhibitors and alkaline, and was hard to be reused. Therefore, one pretreatment method with reduced washing water use need to be developed. NH_3_ has been shown to remove lignin from lignocellulosic materials^14^; NH_3_ residues can be collected by volatilization, eliminating the neutralization step post pretreatment. Furthermore, H_2_O_2_ is able to oxidize the phenolic compounds generated by alkaline pretreatment, which may reduce the use of washing water to remove these inhibitors^28^. Thus, we tested LA fermentation efficiency using NH_3_-H_2_O_2_-pretreated corncob. For the first experiment, LA fermentation was performed using corncob pretreated for 1 day with NH_3_ and then further treated for 1 day with H_2_O_2_ with a NaOH solution as the neutralizer. LA yield, titer, and productivity were 0.42 g/g corncob, 33.70 g/L, and 0.70 g/L/h, respectively (Fig. 2A). For the second fermentation, LA was produced using corncob pretreated for 1 day with NH_3_ and then further treated for 7 days with H_2_O_2_, with a NaOH solution as the neutralizer. The final LA yield, titer, and productivity were 0.50 g/g corncob, 39.93 g/L, and 0.83 g/L/h, respectively (Fig. 2B). LA yield and titer were significantly increased (0.50 g/g corncob vs. 0.42 g/g corncob; and 39.93 g/L vs. 33.67 g/L) when using a substrate with an extended H_2_O_2_ pretreatment time compared to 1-day H_2_O_2_ pretreatment, as shown by independent samples t-test (*p* < 0.05). The total phenolic concentration in both experiments was similar; however, we propose that the phenolic compounds from the NH_3_ pretreatment experiment could be oxidized following extended H_2_O_2_ treatment and thus their inhibitory effects were reduced. One significant difference between these fermentations was that a higher amount of LA was produced at 12 h, and importantly, more xylose was liberated using corncob with extended H_2_O_2_ pretreatment (Fig. 2A,B). However, when corncob with a short H_2_O_2_ pretreatment was used as the substrate, both glucose and xylose were consumed at 12 h and no additional xylose accumulated subsequently (Fig. 2A). These results suggest that the extended H_2_O_2_ pretreatment may also enhance xylose liberation from the raw substrate. Because calcium lactate showed less of an inhibitory effect on *B. coagulans*, we next tested LA fermentation efficiency from NH_3_-H_2_O_2_-pretreated and unwashed corncob using CaCO_3_ as the neutralizer. LA productivity during the initial stage (0 to 12 h) was lower using CaCO_3_ as the neutralizer; additionally, more glucose was liberated from the biomass and accumulated in the fermentation culture (Fig. 2C). However, the overall LA titer, yield, and productivity were 42.61 g/L, 0.53 g/g corncob, and 0.89 g/L/h, respectively (Fig. 2C), which was not significantly different compared with the use of NaOH solution as a neutralizer.

### LA production in fed-batch fermentation using NH_3_-H_2_O_2_-pretreated corncob as a substrate

**Figure 3:**
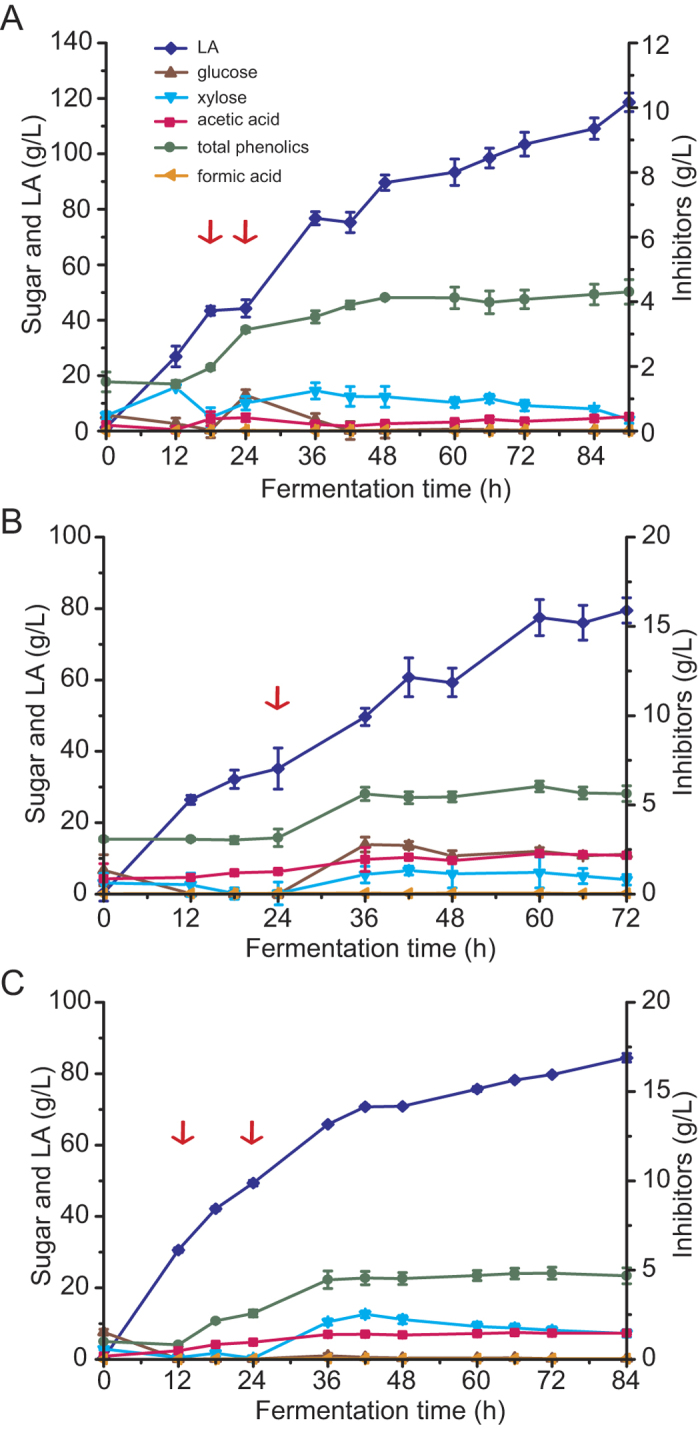
Fed-batch LA fermentation of corncob with 1-day NH_3_ pretreatment followed by 7-days H_2_O_2_ treatment at pH6.0 (adjusted by automatic NaOH solution feeding). (**A**) Concentration of sugars (glucose and xylose), LA, and inhibitors (acetic acid, total phenolics, and formic acid) during fermentation of 8% pretreated and washed corncob substrate fed to 16% corncob from 18 to 24 h. (**B**) Fermentation of 8% pretreated and unwashed corncob substrate fed to 18.4% corncob at 24 h. (**C**) Fermentation of 4% pretreated and washed corncob substrate fed to 8% with pretreated and unwashed corncob at 12 h and fed to 16% with pretreated and unwashed corncob at 24 h. Substrate feeding is indicated by arrows. Blue diamond, LA; red square, acetic acid; green circle, total phenolics; brown triangle, glucose; cyan triangle, xylose; orange triangle, formic acid. Experiments were carried out in duplicate and error bars are shown. Where error bars are not visible, they are smaller than the size of the symbol used.

In order to compare LA fermentation efficiency using NH_3_-H_2_O_2_- and NaOH-pretreated corncob, we first performed fed-batch fermentation using NH_3_-H_2_O_2_-pretreated and washed corncob. CaCO_3_ was used as the neutralizer, similar to the fed-batch fermentation experiment using NaOH-pretreated and washed corncob. The results during the initial stage (0 to 18 h) of fermentation using 8% NH_3_-H_2_O_2_-pretreated and washed corncob were similar to those in Fig. 1C; the LA titer reached 43.39 g/L. The corncob was then fed to 16% (w/w) and cellulase was fed to 30 FPU/g corncob from 18 to 24 h. Fermentation was continued for 90 h and the final LA yield and titer reached 0.74 g/g corncob and 118.60 g/L, respectively, and the overall productivity was 1.32 g/L/h (Fig. 3A, Table 2).There was no significant difference in fermentation efficiency compared to when NaOH-pretreated and wash corncob was used, as shown by independent samples t-test. The l-LA optical purity was 98%. These results indicate that NH_3_-H_2_O_2_ pretreatment efficiently promotes sugar liberation by cellulase and hemicellulase for subsequent LA fermentation, although higher levels of lignin were detected in the pretreated corncob (Table 1). However, NH_3_-H_2_O_2_ pretreatment required 8 days to achieve the same LA titer and yield, while the NaOH method required only 3 h for completion.

In order to fully eliminate the need for washing water, we performed fed-batch fermentation using NH_3_-H_2_O_2_-pretreated (1 day of NH_3_ treatment followed by 7 days of H_2_O_2_ treatment) and unwashed corncob as the substrate. However, in these experiments, we used a NaOH solution as the neutralizer, because the addition of CaCO_3_ to a bioreactor with high solid loading of NH_3_-H_2_O_2_-pretreated corncob led to a viscous fermentation culture, which was difficult to agitate. Fermentation was initiated with 8% unwashed corncob and the substrate was fed to 18.4% (w/w) at 24 h. The total phenolic concentration was 3.0 g/L during the initial stage (0 to 24 h) and increased following substrate feeding to 6.0 g/L. Liberated sugars were consumed rapidly during the initial stage; however, glucose and xylose accumulated in the culture post substrate feeding (Fig. 3B). These results indicate that inhibitors, such as phenolics, inhibited LA fermentation but did not inhibit sugar liberation from the corncob. Finally, the LA titer, yield, and productivity were 79.47 g/L, 0.43 g/g corncob, and 1.10 g/L/h, respectively (Fig. 3B). We hypothesize that initiating fermentation with a small amount of pretreated and washed corncob might increase cell activity and, therefore, enhance LA production efficiency. Thus, in the third experiment, fermentation was initiated with 4% (w/w) NH_3_-H_2_O_2_-pretreated and washed corncob. At this stage, sugars were consumed rapidly, LA was produced at a high rate (titer of 30.60 g/L and yield of 0.77 g/g corncob at 12 h, with productivity of 2.55 g/L/h during this period), and inhibitor concentrations were low (Fig. 3C). The first and second feedings with NH_3_-H_2_O_2_-pretreated and unwashed corncob were conducted at 12 h and 24 h; the substrate was fed to 8% and then to a final concentration of 16% (w/w). Following the feeding, LA was still produced rapidly (12 to 36 h) and the productivity was 1.47 g/L/h during this period. However, once the total phenolic concentration reached a maximum of 4.8 g/L at 36 h and was maintained at that level, LA productivity suddenly decreased to 0.39 g/L/h (36 to 84 h) and xylose accumulated in the culture to a concentration of 8 g/L. Fermentation was continued for 84 h with an LA titer, yield, and productivity of 84.46 g/L, 0.53 g/g corncob, and 1.01 g/L/h, respectively. LA titer and yield could be increased by extending fermentation time; however, this may result in lower overall productivity. In contrast, high-titer LA production was obtained from sulfuric acid-pretreated and biodetoxified corn stover by *P. acidilactici* DQ2. In their studies, acid-pretreated corn stover was subsequently detoxified by inoculation of *Amorphotheca resinae* ZN1 for 5 days in a separated fermentation process and then the biodetoxified corn stover was used for LA fermentation^24^,^25^. Thus, it should be considered to combine NH_3_-H_2_O_2_ pretreatment with biodetoxification to enhance LA fermentation by *B. coagulans* FL204 in our further study. It also should be noted that several additives can enhance fermentation performance. For example, 30 mM citrate buffer greatly influenced acetone-butanol-ethanol fermentation by *Clostridium beijerinckii* when corn stover hydrolysate was used as the substrate^29^. Therefore, useful LA fermentation additives that enhance the yield, titer, and productivity under our pretreatment and fermentation conditions should be identified. Table 3 compares and summarizes LA production from agro-biomass using different pretreatments (acid and alkaline) and fermentation methods (SSF and separate hydrolysis and fermentation [SHF]). This study reports one of the highest LA titers and yields produced from pretreated but unwashed or non-detoxified lignocellulosic materials. Therefore, NH_3_-H_2_O_2_ pretreatment might completely eliminate the need for washing water, at least when corncob is used as the substrate. However, it should also be noted that the LA titer and yield from NH_3_-H_2_O_2_-pretreated corncob are still very low compared with those from NH_3_-H_2_O_2_-pretreated and washed corncob. Moreover, NH_3_-H_2_O_2_ pretreatment is lengthier than the NaOH method, which will impact equipment size, throughput, and process economics.

## Methods

### Raw material and substrate pretreatments

Corncob was harvested in 2014 in the Hubei province of China. After harvest, the corncob was cleaned, dried, and sieved using a 200-mesh. The raw corncob consisted of 37.26 ± 0.56% cellulose, 29.05 ± 0.04% hemicellulose, 19.60 ± 0.64% lignin, and 11.17 ± 0.01% ash. Two pretreatment methods were used in this study. The first method involved pretreating the corncob with 5% NaOH solution at 75 °C for 3 h using 20% (w/w) corncob loading. The resulting slurry was then washed with water until the pH decreased to 8.0 and then filtered to a moisture content of 25% (w/w). In the second method, the corncob was pretreated with 3% (w/w) ammonium hydroxide (NH_3_·H_2_O) for 1 day and then treated with 5% (w/w) H_2_O_2_ solution for 1 day or 7 days using 20% (w/w) corncob loading at room temperature. A portion of the NH_3_-H_2_O_2_-pretreated corncob was washed with water until the pH decreased to 8.0 and then filtered to a moisture content of 25% (w/w). The pH value of the remaining substrate was adjusted to 8.0 using a 50% (w/w) H_2_SO_4_ solution. The cellulase used in this study was Cellic CTec2 (Novozymes, Denmark), which contains cellulase, β-glucosidase, and xylanase activity. YEX medium (10 g/L xylose and 10 g/L yeast extract) was used for seed culturing. The analytical methods of the National Renewable Energy Laboratory (NREL) were used to determine the raw and pretreated material composition in terms of structural carbohydrates and lignin^30^.

### LA fermentation from NaOH-pretreated corncob by SSF

The *B. coagulans* LA204 was inoculated into 200 mL YPX medium, pH6.0 at 50 °C and 100 rpm overnight. Under these conditions, the cells were in a logarithmic growth phase with a concentration of 1.6 × 10^7^ colony forming units (CFU)/mL. The SSF process was established by inoculating 3 L of 8% (w/w) NaOH-pretreated and washed corncob, 10 g/L yeast extract, and 30 filter paper cellulase units (FPU)/g corncob with 300 mL seed culture in a 5-L bioreactor (BAOXING, Shanghai, China). LA fermentation by *B. coagulans* LA204 was performed at optimal conditions (50 °C and pH 6.0 [maintained by an automatic feed of 10 M NaOH solution or using excess CaCO_3_]), as previously reported^12^, for 48 h with agitation at 200 rpm. For the fed-batch experiments, 8% (w/w) NaOH-pretreated and washed corncob, 150 mL seed culture, and 30 FPU/g corncob of cellulase were used to establish fermentation at 50 °C for 18 h in a 1.5 L volume. Washed corncob was fed to 16% (w/w) and enzyme was fed from 18 h to 24 h to maintain a concentration of 30 FPU/g substrate; the total fermentation volume was 3 L. Samples were collected every 6 or 12 h during fermentation and the concentrations of LA, acetic acid, formic acid, glucose, xylose, and total phenolic compounds were determined and the yields and productivities were calculated. Fermentations were performed in duplicate under non-sterile conditions.

### LA fermentation from NH_3_-H_2_O_2_-pretreated corncob by SSF

To establish the SSF process, a 3-L volume of 8% (w/w) NH_3_-H_2_O_2_-pretreated and unwashed corncob, 10 g/L yeast extract, and 30 FPU/g substrate of cellulase were inoculated with 300 mL seed culture in a 5-L bioreactor. Fermentations were carried out at 50 °C for 48 h with agitation at 200 rpm; the pH was maintained at 6.0 using an automatic feed of 10 M NaOH solution or using excess CaCO_3_. Two fed-batch fermentations were carried out to determine LA production ability. In the first fed-batch experiment, 8% NH_3_-H_2_O_2_-pretreated and unwashed corncob, 30 FPU/g substrate of cellulase, and 150 mL seed culture were used to initiate fermentation under the same fermentation conditions in a 1.5-L volume. The NH_3_-H_2_O_2_-pretreated and unwashed corncob was fed to 18.4% (w/w) and cellulase was fed to maintain 30 FPU/g substrate at 24 h; the final volume was 3 L. In the second fed-batch experiment, 4% (w/w) NH_3_-H_2_O_2_-pretreated and washed corncob, 30 FPU/g substrate of cellulase, and 150 mL seed culture were used to initiate fermentation under the same fermentation conditions in a 1.5-L volume. The NH_3_-H_2_O_2_-pretreated and unwashed corncob was fed to 8% (w/w) at 12 h and to 16% at 24 h and cellulase was fed to maintain 30 FPU/g substrate; the final volume was 3 L. In addition, fed-batch fermentations using NH_3_-H_2_O_2_-pretreated and washed corncob were also conducted using CaCO_3_ as the pH neutralizer; 8% NH_3_-H_2_O_2_-pretreated and washed corncob, 30 FPU/g substrate of cellulase, and 150 mL seed culture were used to initiate fermentation under the same fermentation conditions in a 1.5-L volume. The NH_3_-H_2_O_2_ pretreated and washed corncob was fed to 16% (w/w) and cellulase was fed to maintain 30 FPU/g substrate from 18 h to 24 h; the final volume was 3 L. Samples were collected every 6 or 12 h during fermentation and the concentrations of LA, acetic acid, formic acid, glucose, xylose, and total phenolic compounds were determined and the yields and productivities were calculated. These fermentations were conducted in duplicate under non-sterile condition. The crude HPLC data were included in Supplementary Figure 1.

### Analysis of sugars, lactic acid, and inhibitors

The levels of glucose, xylose, LA, acetic acid, and formic acid in the samples were measured using high performance liquid chromatography (HPLC) with a Bio-Rad HPX-87H ion-exclusion column equipped with an Agilent 1200 and a RID-10A or SPD-20A detector. The mobile phase was 5 mM H_2_SO_4_ at a flow rate of 0.6 mL/min at 40 °C. All samples were centrifuged at 12,000 rpm for 2 min and filtered using nylon syringe filters (pore size 0.22 μm) prior to loading. All standards for HPLC analysis (glucose, xylose, lactic acid, acetic acid, and formic acid) were obtained from Sigma-Aldrich. The concentration of l-LA was determined using an SBA-40X biosensor (Shandong Biosensor Institute, China). The total content of phenolic compounds in the samples was determined using the Folin-Ciocalteu method^31^ with gallic acid as a calibration standard.

## Additional Information

**How to cite this article**: Zhang, Z. *et al.* Comparison of high-titer lactic acid fermentation from NaOH- and NH_3_-H_2_O_2_-pretreated corncob by *Bacillus coagulans* using simultaneous saccharification and fermentation. *Sci. Rep.* **6**, 37245; doi: 10.1038/srep37245 (2016).

**Publisher’s note**: Springer Nature remains neutral with regard to jurisdictional claims in published maps and institutional affiliations.

## Supplementary Material

Supplementary Information

## Figures and Tables

**Table 1:** Composition of the contents in raw and pretreated corncob materials (% dry matter).

Sample	Cellulose (as glucose,%)	Hemicellulose (as xylose,%)	Lignin (%)	Ash (%)
Without pretreatment	37.26 ± 0.56	29.05 ± 0.04	19.60 ± 0.64	11.17 ± 0.01
NaOH (wash)	59.84 ± 0.94	19.99 ± 0.01	6.28 ± 1.05	11.34 ± 0.00
NH_3_-H_2_O_2_ (wash, 7d)	44.54 ± 2.80	26.21 ± 0.01	17.10 ± 0.15	10.16 ± 0.01
NH_3_-H_2_O_2_ (unwash, 1d)	38.92 ± 0.97	28.85 ± 0.01	19.00 ± 0.21	10.97 ± 0.00
NH_3_-H_2_O_2_ (unwash, 7d)	42.14 ± 1.15	29.12 ± 0.03	16.61 ± 0.51	10.12 ± 0.00

**Table 2:** Summary of lactic acid fermentation by *Bacillus coagulans* LA204 using corncob as carbon source by SSF.

corncob concentration	8%	8%	8–16%	8%	8%	8%	8–16%	8–18.4%	4-8-16%
Pretreatment	NaOH	NaOH	NaOH	NH_3_-H_2_O_2_^a^	NH_3_-H_2_O_2_^b^	NH_3_-H_2_O_2_^b^	NH_3_-H_2_O_2_^b^	NH_3_-H_2_O_2_^b^	NH_3_-H_2_O_2_^b^
Washing^c^	Y	Y	Y-Y	N	N	N	Y-Y	N-N	Y-N-N
Neutralizer	NaOH	CaCO_3_	CaCO_3_	NaOH	NaOH	CaCO_3_	CaCO_3_	NaOH	NaOH
Lactic acid titer (g/L)	62.91 ± 1.36	72.62 ± 2.14	122.99 ± 6.37	33.67 ± 1.68	39.93 ± 2.71	42.61 ± 2.35	118.60 ± 3.37	79.47 ± 3.55	84.46 ± 1.21
Lactic acid yield (g/g corncob)	0.79	0.91	0.77	0.42	0.50	0.53	0.74	0.43	0.53
Lactic acid productivity (g/L/h)	1.31	1.51	1.37	0.70	0.83	0.89	1.32	1.10	1.01
Acetic acid titer (g/L)	0.56 ± 0.04	0.67 ± 0.01	0.5 ± 0.09	1.06 ± 0.11	1.23 ± 0.22	1.17 ± 0.16	0.45 ± 0.09	2.17 ± 0.17	1.46 ± 0.07
Acetic acid yield (g/g corncob)	0.01	0.01	0.003	0.01	0.02	0.01	0.003	0.01	0.01
Acetic acid productivity (g/L/h)	0.01	0.01	0.01	0.02	0.03	0.02	0.01	0.03	0.02

^a^NH_3_ pretreatment for 1 day and sequential H_2_O_2_ pretreatment for 1 day.

^b^7 days.

^c^Y stands for washed and N for unwashed after alkaline pretreatment.

**Table 3:** Summary of recent publications on lactic acid production from agro-biomass.

Strain	Substrate	Pretreatment	Detoxification	Fermentation mode	Lactic acid			Optical purity	Ref.
					titer (g/L)	yield (g/g)	productivity (g/L/h)		
*B. coagulans* strain IPE22	wheat straw (water insoluble solid after pretreatment)	sulfuric acid	—	SSF	38.73	0.46^a^	0.43	N.D.	23
*B. coagulans* MXL-9	corn fiber hydrolysate	sulfuric acid	—	SHF	39	0.39^a^	0.54	99% L-LA	16
*B. coagulans* DSM 2314	wheat straw	lime	—	fed-batch SSF	40.7	0.43^a^/0.81^c^	0.68	97.2% L-LA	21
*B. coagulans* LA204	corn stover	NaOH	water washing	fed-batch SSF	97.59	0.68^a^	1.63	97.9% L-LA	12
*B. coagulans* JI12	hydrolysate of oil palm empty fruit bunch	sulfuric acid and phosphoric acid	—	fed-batch SHF	137.5	0.98^b^	4.4	99.5% L-LA	22
*Bacillus* sp. strain NL01	corn stover hydrolysate	Steam explosion followed by enzyme saccharification	water washing	fed-batch SHF	75	0.75^b^	1.04	N.D.	32
*Lb. rhamnosus* and *Lb. brevis*	corn stover	NaOH	water washing	fed-batch SSF	60.3	0.7^a^	0.58	N.D.	33
*Lb. plantarum* NCIMB 8826	corn stover	NaOH	—	fed-batch SSF	61.4	0.77^d^	0.32	99% D-LA	34
*Lb. pentosus* FL0421	corn stover	NaOH	water washing	fed-batch SSF	92.3	0.66^a^	1.92	98.1% L-LA	35
*P. acidilactici* DQ2	corn stover	dilute sulphuric acid	bio-detoxification	SSF	101.9	0.77^e^	1.06	63.4% L-LA	25
*P. acidilactici* TY112	corn stover	dilute sulphuric acid	bio-detoxification	SSF	104.4/77.76	0.72^e^/0.65^e^	1.06	99.89% L-LA	24,36
*P. acidilactici* ZP26	corn stover	dilute sulphuric acid	bio-detoxification	SSF	76.76	0.58^e^	1.02	99.32% D-LA	36
*R. oryzae* HZS6	corncob hydrolysate	sulfuric acid	—	SHF	77.2	0.80^b^	0.99	100% L-LA	37
*B. coagulans* LA204	corncob	NaOH/NH_3_-H_2_O_2_	water washing	fed-batch SSF	120.99/118.60	0.77^a^/0.74^a^	1.37/1.32	98% L-LA	This study
		NH_3_-H_2_O_2_	—		79.47	0.43^a^	1.10		

^a^g/g total stover.

^b^g/g total sugar in the hydrolysate.

^c^g/g released total sugar.

^d^g/g used stover.

^e^g/g glucose from total cellulose. N.D.: not determined.
