# Supplementary material for: Comparison of high-titer lactic acid fermentation from NaOH- and NH3-H2O2-pretreated corncob by Bacillus coagulans using simultaneous saccharification and fermentation
Source: Sci Rep. 2016 Nov 17;6:37245. doi: 10.1038/srep37245 (PMC5112544; doi:10.1038/srep37245)
Supplement: Supplementary Information [file srep37245-s1.pdf]

Comparison of high-titer lactic acid fermentation from NaOH- and NH_3-H_2O_2-pretreated corncob by *Bacillus coagulans* using simultaneous saccharification and fermentation

Zhenting Zhang[a], Yuejiao Xie[a], Xiaolan He[a], Xinli Li[a], Jinlong Hu[a], Zhiyong Ruan[b], Shumiao Zhao[a, c], Nan Peng[a, c, d]*, Yunxiang Liang[a, c]*

[a]State Key Laboratory of Agricultural Microbiology, College of Life Science and Technology, Huazhong Agricultural University, Wuhan, 430070, Hubei, P.R. China

[b]Key Laboratory of Microbial Resources (Ministry of Agriculture, China), Institute of Agricultural Resources and Regional Planning, CAAS, Beijing, 100081, China

[c]Hubei Collaborative Innovation Center for Industrial Fermentation, Wuhan, 430068, P. R. China

[d]Key Laboratory of Development and Application of Rural Renewable Energy (Ministry of Agriculture), Biomass Energy Technology Research Centre, Biogas Institute of Ministry of Agriculture, Chengdu, 610041, Sichuan, P. R. China

*To whom correspondence should be addressed.

Tel: +86 27 8728 1267; Fax: +86 27 8728 0670; Email: nanp@mail.hzau.edu.cn or fa-lyx@163.com

Figure S1

A: The curves of fermentation on 8% (w/w) NaOH pretreated and washed corncob at pH6.0 adjusted by automatic feeding of NaOH solution

0 h

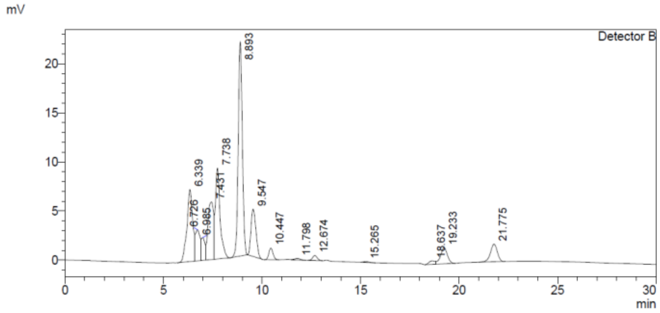

Detector B

Peak#	Ret. Time	Area	Height	Conc.
1	6.339	150039	7347	14.623
2	6.726	51667	3169	5.035
3	6.985	28872	2325	2.814
4	7.431	119083	5862	11.606
5	7.738	154500	9248	15.057
6	8.893	318255	21772	31.016
7	9.547	82450	4810	8.035
8	10.447	17569	1186	1.712
9	11.798	3247	149	0.316
10	12.674	8556	521	0.834
11	15.265	1477	85	0.144
12	18.637	7651	353	0.746
13	19.233	39291	1637	3.829
14	21.775	43425	1791	4.232
Total		1026082	60254	

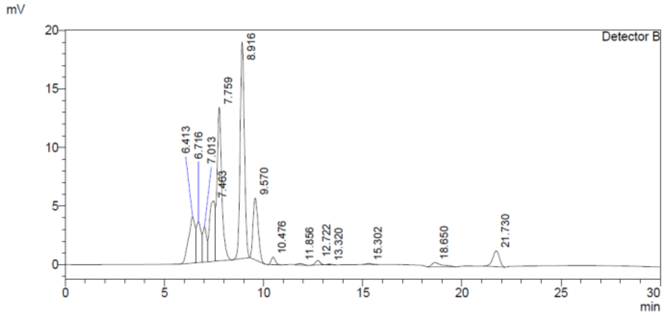

Detector B

Peak#	Ret. Time	Area	Height	Conc.
1	6.413	89325	3950	9.468
2	6.716	58096	3462	6.158
3	7.013	41758	2985	4.426
4	7.463	101078	5167	10.714
5	7.759	216929	13080	22.994
6	8.916	277531	18447	29.418
7	9.570	92364	5263	9.790
8	10.476	9673	646	1.025
9	11.856	2985	144	0.316
10	12.722	6560	393	0.695
11	13.320	1049	70	0.111
12	15.302	1884	100	0.200
13	18.650	12865	360	1.364
14	21.730	31317	1358	3.320
Total		943413	55424	

12 h

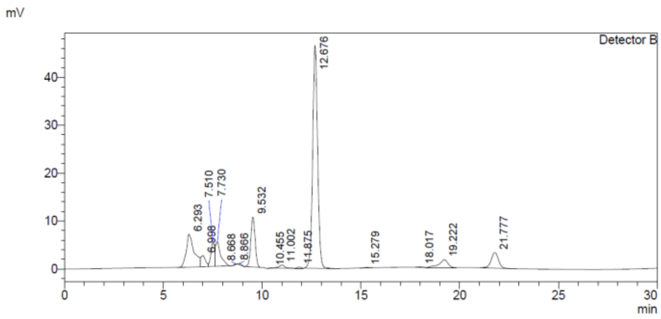

Detector B

Peak#	Ret. Time	Area	Height	Conc.
1	6.293	189410	6871	12.516
2	6.998	39508	2345	2.611
3	7.510	66688	4960	4.407
4	7.730	94252	5111	6.228
5	8.668	1437	157	0.095
6	8.866	3283	311	0.217
7	9.532	155290	10405	10.261
8	10.455	604	54	0.040
9	11.002	13141	676	0.868
10	11.875	3432	263	0.227
11	12.676	808264	46469	53.409
12	15.279	1736	101	0.115
13	18.017	1728	103	0.114
14	19.222	51984	1681	3.435
15	21.777	82578	3267	5.457
Total		1513336	82774	

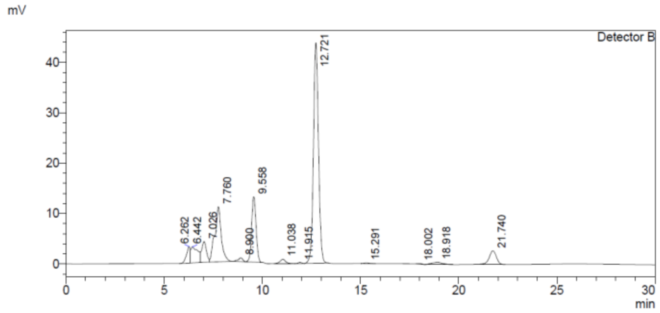

Detector B

Peak#	Ret. Time	Area	Height	Conc.
1	6.262	43483	3274	2.819
2	6.442	76296	3188	4.947
3	7.026	71329	4148	4.625
4	7.760	248920	10921	16.139
5	8.900	11982	746	0.777
6	9.558	199992	12972	12.966
7	11.038	18102	895	1.174
8	11.915	2681	200	0.174
9	12.721	781574	43706	50.673
10	15.291	2217	122	0.144
11	18.002	1436	90	0.093
12	18.918	17398	438	1.128
13	21.740	66967	2710	4.342
Total		1542377	83411	

18 h

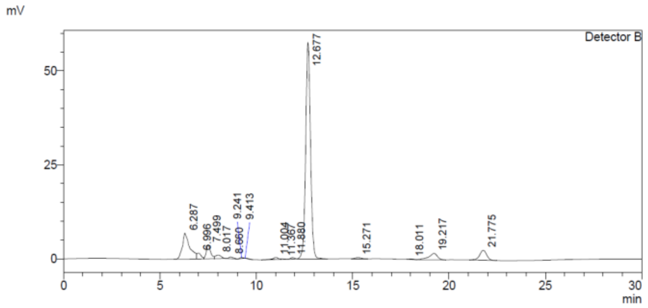

Detector B

Peak#	Ret. Time	Area	Height	Conc.
1	6.287	184506	6872	12.780
2	6.996	22275	1521	1.543
3	7.499	57461	3683	3.980
4	8.017	20738	952	1.436
5	8.660	7765	505	0.538
6	9.241	3381	278	0.234
7	9.413	1028	16	0.071
8	11.004	10908	567	0.756
9	11.367	1719	116	0.119
10	11.880	4871	366	0.337
11	12.677	1001843	57512	69.395
12	15.271	5516	292	0.382
13	18.011	1746	110	0.121
14	19.217	53661	1719	3.717
15	21.775	66258	2650	4.590
Total		1443677	77158	

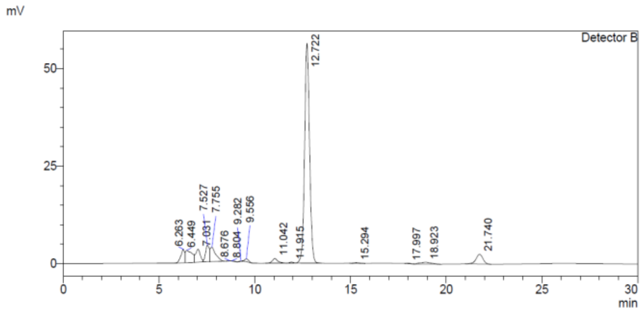

Detector B

Peak#	Ret. Time	Area	Height	Conc.
1	6.263	46801	3456	3.230
2	6.449	76627	3165	5.289
3	7.031	56427	3296	3.895
4	7.527	58073	4245	4.008
5	7.755	73737	3707	5.089
6	8.676	899	99	0.062
7	8.804	965	31	0.067
8	9.282	1546	141	0.107
9	9.556	10322	735	0.712
10	11.042	25831	1226	1.783
11	11.915	3536	266	0.244
12	12.722	1009592	56312	69.680
13	15.294	3957	210	0.273
14	17.997	1266	80	0.087
15	18.923	18058	468	1.246
16	21.740	61253	2486	4.228
Total		1448891	79923	

24 h

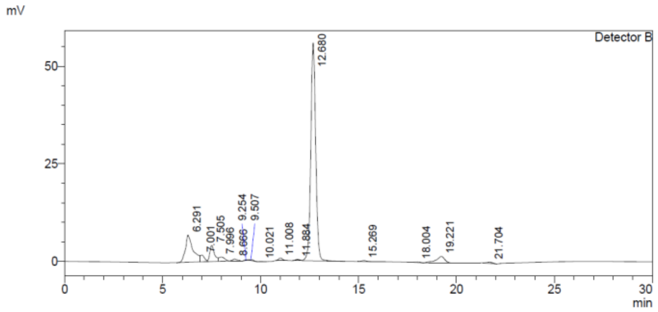

Detector B

Peak#	Ret. Time	Area	Height	Conc.
1	6.291	189098	6998	13.735
2	7.001	25464	1721	1.849
3	7.505	69050	4149	5.015
4	7.996	21210	1070	1.541
5	8.666	7724	489	0.561
6	9.254	1494	143	0.108
7	9.507	3082	216	0.224
8	10.021	358	35	0.026
9	11.008	9037	636	0.656
10	11.884	5935	390	0.431
11	12.680	980688	55873	71.229
12	15.269	4073	225	0.296
13	18.004	1867	110	0.136
14	19.221	51662	1666	3.752
15	21.704	6060	296	0.440
Total		1376803	74017	

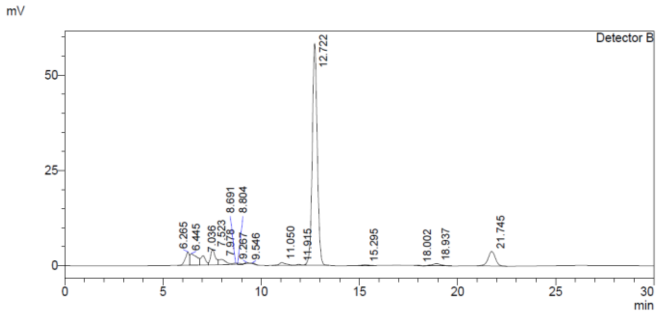

Detector B

Peak#	Ret. Time	Area	Height	Conc.
1	6.265	49043	3536	3.340
2	6.445	74649	3054	5.084
3	7.036	41129	2471	2.801
4	7.523	68562	3964	4.670
5	7.978	33202	1377	2.261
6	8.691	4586	332	0.312
7	8.804	2376	262	0.162
8	9.267	3773	333	0.257
9	9.546	2869	214	0.195
10	11.050	19263	805	1.312
11	11.915	3609	273	0.246
12	12.722	1042036	58144	70.973
13	15.295	4716	250	0.321
14	18.002	1464	89	0.100
15	18.937	20729	584	1.412
16	21.745	96209	3788	6.553
Total		1468214	79478	

36 h

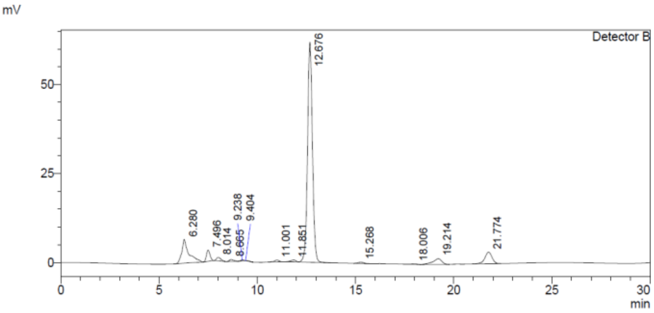

Detector B

Peak#	Ret. Time	Area	Height	Conc.
1	6.280	168165	6684	11.427
2	7.496	40937	3150	2.782
3	8.014	15895	1004	1.080
4	8.665	8621	535	0.586
5	9.238	3697	315	0.251
6	9.404	1658	37	0.113
7	11.001	8600	512	0.584
8	11.851	9170	512	0.623
9	12.676	1074099	61687	72.984
10	15.268	6415	347	0.436
11	18.006	1615	99	0.110
12	19.214	51693	1631	3.512
13	21.774	81124	3223	5.512
Total		1471687	79737	

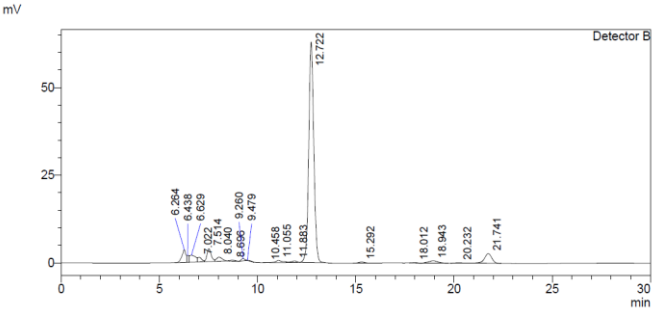

Detector B

Peak#	Ret. Time	Area	Height	Conc.
1	6.264	50926	3639	3.458
2	6.438	15334	1970	1.041
3	6.629	40688	1971	2.763
4	7.022	18939	1339	1.286
5	7.514	55131	3675	3.744
6	8.040	24068	1196	1.634
7	8.696	5355	301	0.364
8	9.260	8275	628	0.562
9	9.479	1613	37	0.110
10	10.458	351	27	0.024
11	11.055	15132	623	1.028
12	11.883	8046	434	0.546
13	12.722	1127446	62920	76.564
14	15.292	5873	310	0.399
15	18.012	1961	115	0.133
16	18.943	23712	715	1.610
17	20.232	1810	73	0.123
18	21.741	67902	2727	4.611
Total		1472561	82701	

42 h

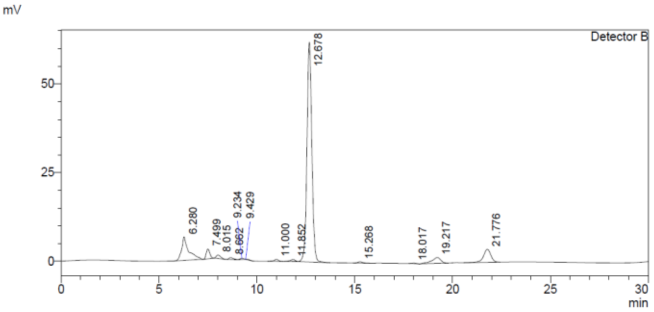

Detector B

Peak#	Ret. Time	Area	Height	Conc.
1	6.280	166044	6621	11.172
2	7.499	35691	2740	2.401
3	8.015	15771	1009	1.061
4	8.662	8084	536	0.544
5	9.234	4259	341	0.287
6	9.429	1741	72	0.117
7	11.000	8818	525	0.593
8	11.852	9495	524	0.639
9	12.678	1080678	61961	72.709
10	15.268	6941	373	0.467
11	18.017	1676	102	0.113
12	19.217	53160	1684	3.577
13	21.776	93954	3653	6.321
Total		1486312	80141	

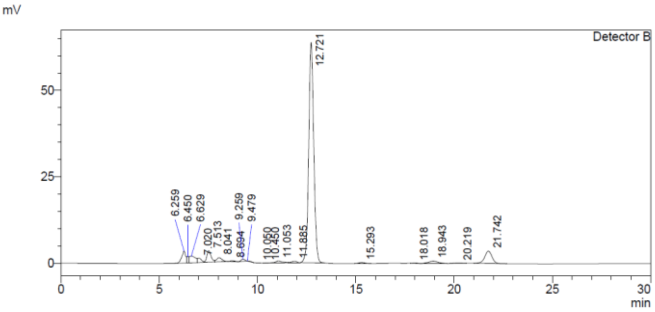

Detector B

Peak#	Ret. Time	Area	Height	Conc.
1	6.259	47383	3455	3.171
2	6.450	13311	1809	0.891
3	6.629	40897	1921	2.737
4	7.020	17305	1230	1.158
5	7.513	49670	3325	3.324
6	8.041	23103	1184	1.546
7	8.694	4364	277	0.292
8	9.259	8441	647	0.565
9	9.479	1235	44	0.083
10	10.050	27	6	0.002
11	10.450	356	27	0.024
12	11.053	14319	626	0.958
13	11.885	8128	444	0.544
14	12.721	1140257	63701	76.306
15	15.293	6074	319	0.406
16	18.018	1896	113	0.127
17	18.943	24418	744	1.634
18	20.219	2542	99	0.170
19	21.742	90605	3555	6.063
Total		1494330	83526	

48 h

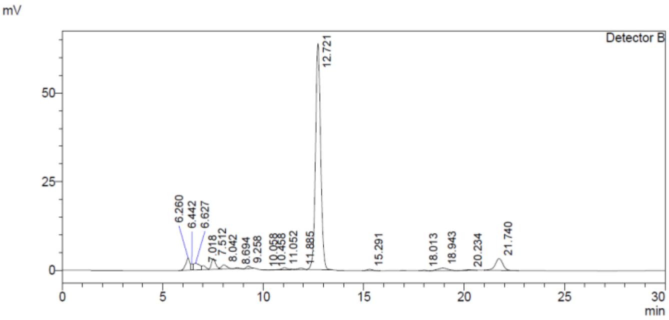

Detector B

Peak#	Ret. Time	Area	Height	Conc.
1	6.260	48445	3497	3.269
2	6.442	13144	1791	0.887
3	6.627	38852	1859	2.622
4	7.018	14994	1115	1.012
5	7.512	44874	3029	3.028
6	8.042	22011	1162	1.485
7	8.694	4323	283	0.292
8	9.258	9981	703	0.673
9	10.058	72	10	0.005
10	10.458	331	25	0.022
11	11.052	13664	633	0.922
12	11.885	8278	450	0.559
13	12.721	1142869	63804	77.118
14	15.291	6166	325	0.416
15	18.013	2017	117	0.136
16	18.943	25090	770	1.693
17	20.234	3130	118	0.211
18	21.740	83726	3297	5.650
Total		1481966	82988	

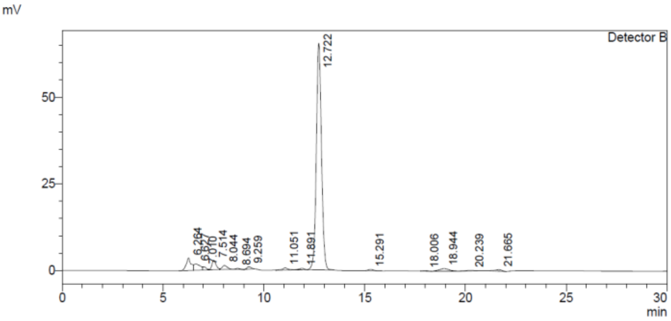

Detector B

Peak#	Ret. Time	Area	Height	Conc.
1	6.264	62374	3626	4.368
2	6.627	37844	1814	2.651
3	7.010	12386	968	0.868
4	7.514	39873	2712	2.793
5	8.044	21089	1167	1.477
6	8.694	4600	303	0.322
7	9.259	10221	709	0.716
8	11.051	12484	646	0.874
9	11.891	8373	465	0.586
10	12.722	1172139	65423	82.094
11	15.291	6473	338	0.453
12	18.006	1680	101	0.118
13	18.944	25834	812	1.809
14	20.239	3572	137	0.250
15	21.665	8864	385	0.621
Total		1427806	79608	

B: The curves of fermentation on 8% (w/w) NaOH pretreated and washed corncob at pH6.0 adjusted by $CaCO_3$

0 h

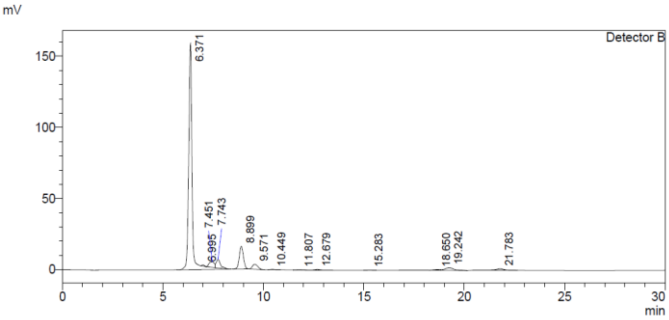

Detector B

Peak#	Ret. Time	Area	Height	Conc.
1	6.371	1883745	159170	76.653
2	6.995	7172	840	0.292
3	7.451	69187	3897	2.815
4	7.743	91196	5991	3.711
5	8.899	233658	16035	9.508
6	9.571	64544	3462	2.626
7	10.449	4882	345	0.199
8	11.807	2555	127	0.104
9	12.679	7351	451	0.299
10	15.283	1716	99	0.070
11	18.650	9456	434	0.385
12	19.242	58770	1994	2.391
13	21.783	23274	1001	0.947
Total		2457507	193845	

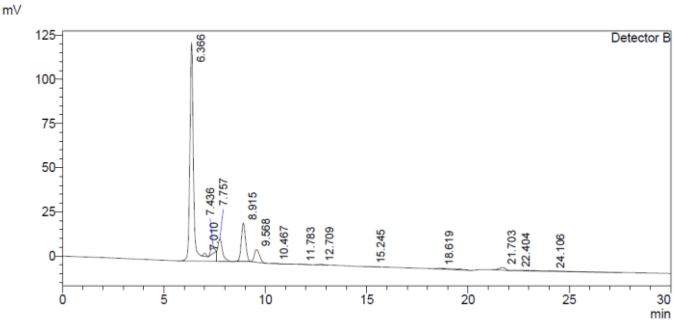

Detector B

Peak#	Ret. Time	Area	Height	Conc.
1	6.366	1623076	122892	65.525
2	7.010	16045	1747	0.648
3	7.436	46453	2794	1.875
4	7.757	213424	12951	8.616
5	8.915	325065	21724	13.123
6	9.568	125165	7189	5.053
7	10.467	4690	329	0.189
8	11.783	2366	113	0.096
9	12.709	8077	488	0.326
10	15.245	1344	79	0.054
11	18.619	37881	494	1.529
12	21.703	32148	1495	1.298
13	22.404	20149	277	0.813
14	24.106	21152	206	0.854
Total		2477035	172779	

12 h

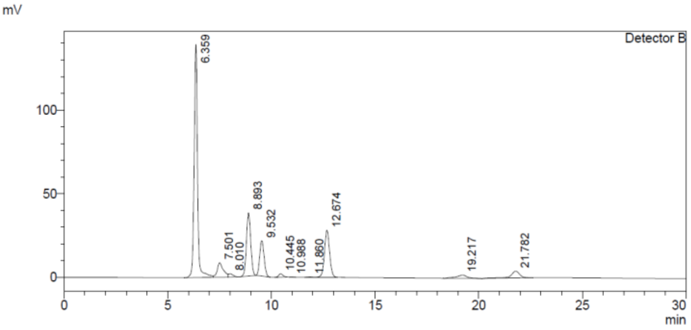

Detector B

Peak#	Ret. Time	Area	Height	Conc.
1	6.359	1573475	139497	47.111
2	7.501	178832	8592	5.354
3	8.010	28020	1845	0.839
4	8.893	545421	37743	16.330
5	9.532	305960	21092	9.161
6	10.445	28022	1945	0.839
7	10.988	828	82	0.025
8	11.860	2517	180	0.075
9	12.674	484631	28140	14.510
10	19.217	71177	1982	2.131
11	21.782	121072	4065	3.625
Total		3339955	245165	

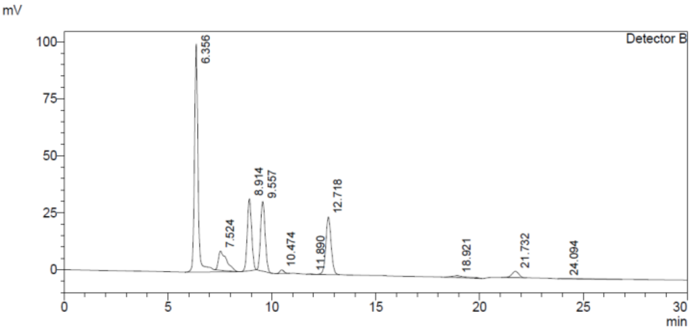

Detector B

Peak#	Ret. Time	Area	Height	Conc.
1	6.356	1241729	99756	41.555
2	7.524	232468	8426	7.780
3	8.914	464790	31533	15.554
4	9.557	458671	30634	15.349
5	10.474	23567	1617	0.789
6	11.890	2085	152	0.070
7	12.718	447533	25198	14.977
8	18.921	42814	808	1.433
9	21.732	66246	2800	2.217
10	24.094	8286	103	0.277
Total		2988189	201028	

18 h

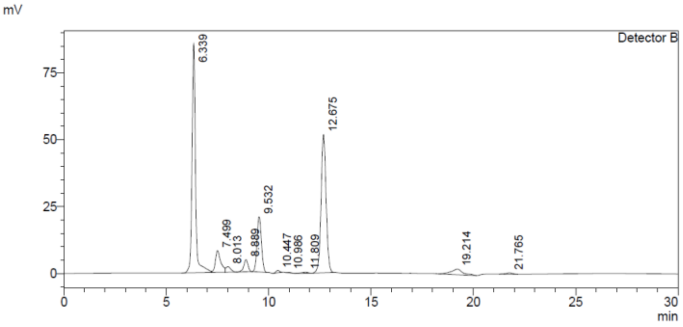

Detector B

Peak#	Ret. Time	Area	Height	Conc.
1	6.339	1013819	85616	39.407
2	7.499	151071	8186	5.872
3	8.013	34156	2095	1.328
4	8.889	65802	4495	2.558
5	9.532	303191	20666	11.785
6	10.447	10444	848	0.406
7	10.986	1432	140	0.056
8	11.809	7339	330	0.285
9	12.675	891070	51702	34.636
10	19.214	83288	2134	3.237
11	21.765	11044	518	0.429
Total		2572656	176732	

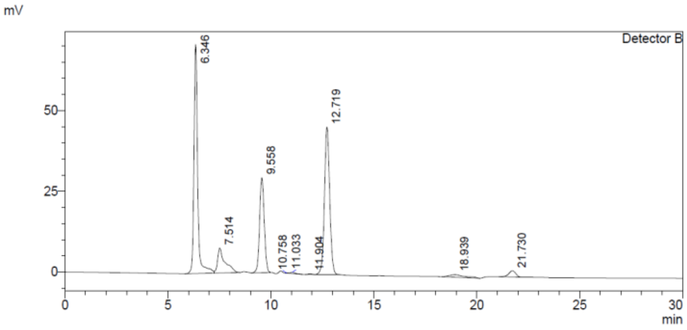

Detector B

Peak#	Ret. Time	Area	Height	Conc.
1	6.346	873850	70822	35.991
2	7.514	191163	7685	7.873
3	9.558	452944	29374	18.655
4	10.758	385	39	0.016
5	11.033	6070	410	0.250
6	11.904	2144	166	0.088
7	12.719	812904	45586	33.481
8	18.939	43541	849	1.793
9	21.730	44986	1932	1.853
Total		2427986	156862	

24 h

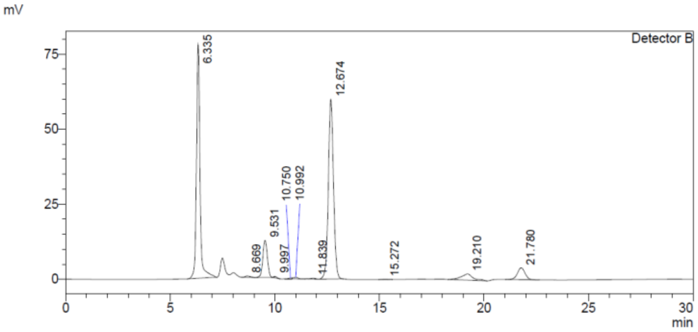

Detector B

Peak#	Ret. Time	Area	Height	Conc.
1	6.335	915887	77900	39.024
2	8.669	7958	431	0.339
3	9.531	182814	12356	7.789
4	9.997	5074	472	0.216
5	10.750	2677	248	0.114
6	10.992	7912	521	0.337
7	11.839	4406	257	0.188
8	12.674	1034028	59848	44.058
9	15.272	1760	99	0.075
10	19.210	82173	2096	3.501
11	21.780	102275	3972	4.358
Total		2346964	158198	

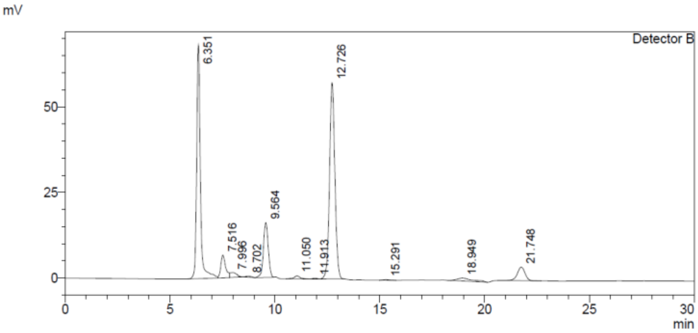

Detector B

Peak#	Ret. Time	Area	Height	Conc.
1	6.351	843680	68296	34.694
2	7.516	106747	6630	4.390
3	7.996	30870	1392	1.269
4	8.702	5922	354	0.244
5	9.564	249822	16019	10.273
6	11.050	19002	922	0.781
7	11.913	3423	255	0.141
8	12.726	1023489	57392	42.088
9	15.291	2577	140	0.106
10	18.949	45762	925	1.882
11	21.748	100504	3976	4.133
Total		2431798	156302	

36 h

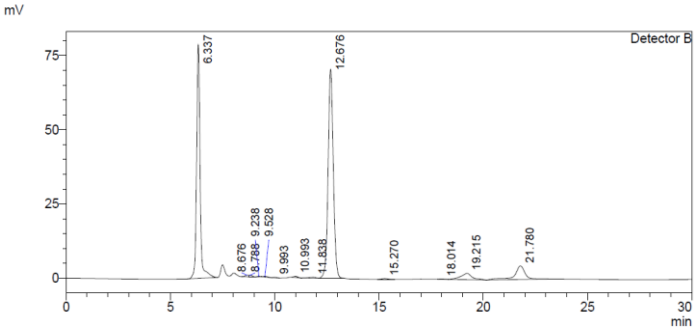

Detector B

Peak#	Ret. Time	Area	Height	Conc.
1	6.337	917273	78655	38.100
2	8.676	6515	523	0.271
3	8.788	4323	442	0.180
4	9.238	1722	158	0.072
5	9.528	3294	233	0.137
6	9.993	2673	222	0.111
7	10.993	5413	446	0.225
8	11.838	6658	334	0.277
9	12.676	1218913	70295	50.629
10	15.270	4959	269	0.206
11	18.014	1236	75	0.051
12	19.215	80739	2128	3.354
13	21.780	153805	4554	6.389
Total		2407522	158334	

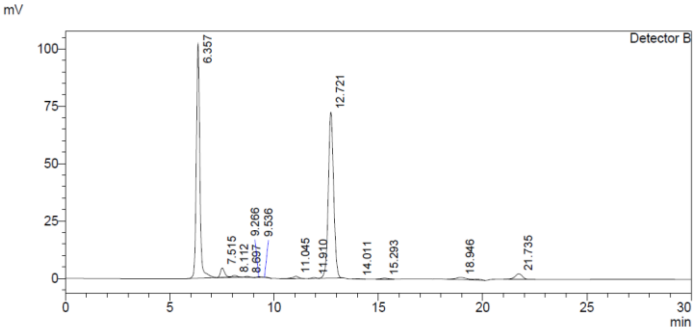

Detector B

Peak#	Ret. Time	Area	Height	Conc.
1	6.357	1205007	101927	44.253
2	7.515	58655	4121	2.154
3	8.112	13839	726	0.508
4	8.697	8001	470	0.294
5	9.266	3651	316	0.134
6	9.536	2402	137	0.088
7	11.045	19319	1036	0.709
8	11.910	4848	357	0.178
9	12.721	1294689	72378	47.546
10	14.011	1882	126	0.069
11	15.293	6792	352	0.249
12	18.946	45561	981	1.673
13	21.735	58361	2411	2.143
Total		2723007	185337	

42 h

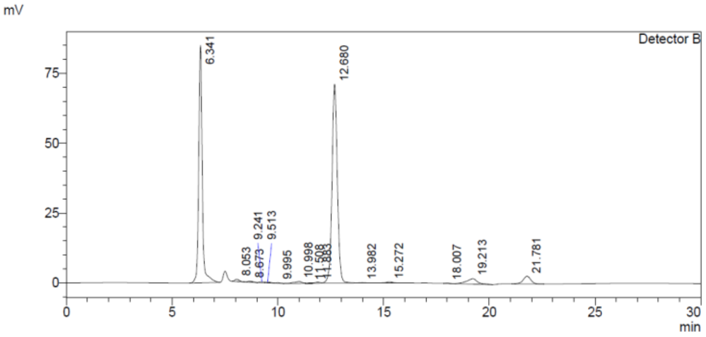

Peak#	Ret. Time	Area	Height	Conc.
1	6.338	980624	84725	41.203
2	8.041	16094	1020	0.676
3	8.668	8207	458	0.345
4	9.245	2042	190	0.086
5	9.531	2895	193	0.122
6	9.987	1399	129	0.059
7	10.758	3478	319	0.146
8	10.998	10491	656	0.441
9	11.873	4094	280	0.172
10	12.678	1253306	71894	52.660
11	13.986	3215	211	0.135
12	15.272	13323	687	0.560
13	18.052	7724	399	0.325
14	19.220	63511	1896	2.669
15	21.743	9572	456	0.402
Total		2379974	163514	

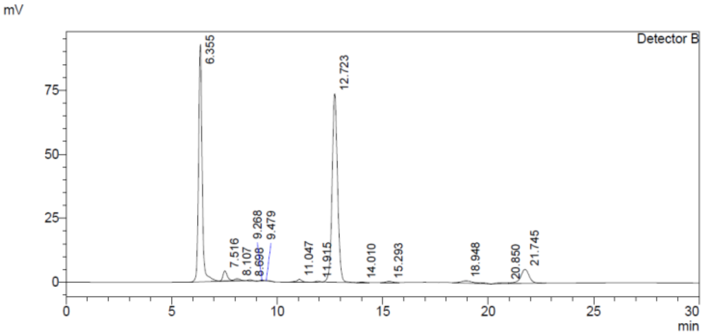

Peak#	Ret. Time	Area	Height	Conc.
1	6.355	1103316	92547	40.038
2	7.516	56047	3927	2.034
3	8.107	14099	747	0.512
4	8.698	7651	466	0.278
5	9.268	4281	342	0.155
6	9.479	1743	26	0.063
7	11.047	18704	1037	0.679
8	11.915	4798	358	0.174
9	12.723	1324038	73618	48.048
10	14.010	2332	151	0.085
11	15.293	7922	407	0.287
12	18.948	40983	924	1.487
13	20.850	13976	352	0.507
14	21.745	155762	5517	5.652
Total		2755653	180419	

48 h

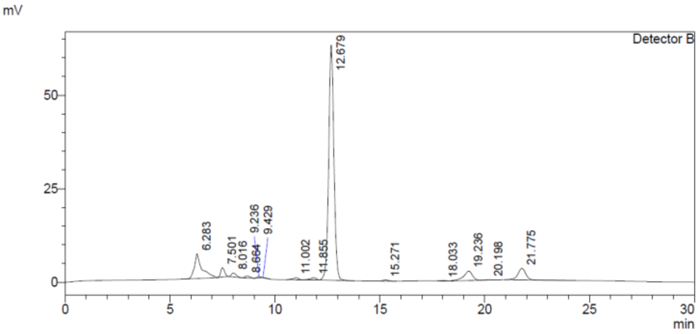

Peak#	Ret. Time	Area	Height	Conc.
1	6.339	1002781	85890	40.857
2	7.499	54154	3830	2.206
3	8.044	18418	1075	0.750
4	8.671	7107	428	0.290
5	9.238	2136	191	0.087
6	9.502	2077	111	0.085
7	9.988	2108	155	0.086
8	10.758	3918	324	0.160
9	10.994	10409	666	0.424
10	11.870	3823	279	0.156
11	12.676	1251482	71920	50.990
12	13.969	1129	81	0.046
13	15.269	7975	428	0.325
14	18.034	2208	126	0.090
15	19.212	72314	2007	2.946
16	21.745	12312	517	0.502
Total		2454351	168027	

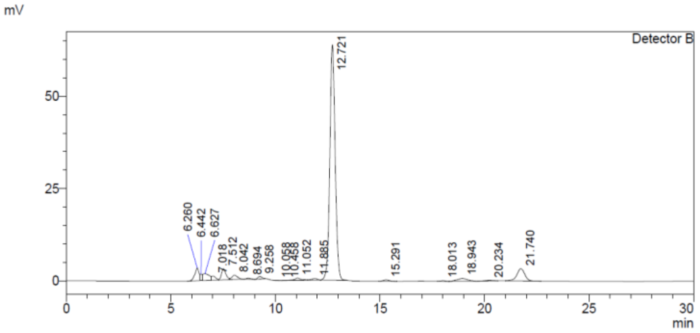

Peak#	Ret. Time	Area	Height	Conc.
1	6.354	1045715	87912	41.271
2	7.516	46725	3307	1.844
3	8.092	14550	812	0.574
4	8.698	6794	427	0.268
5	9.272	3832	304	0.151
6	9.454	2115	46	0.083
7	11.049	18984	1056	0.749
8	11.917	5114	377	0.202
9	12.724	1329378	74553	52.466
10	14.013	2776	180	0.110
11	15.294	10088	515	0.398
12	18.010	2388	135	0.094
13	18.948	37382	894	1.475
14	21.658	7941	374	0.313
Total		2533782	170892	

C: The curves of fed-batch fermentation from 8% to 16% (w/w) NaOH pretreated and washed corncob at pH6.0 adjusted by $CaCO_3$

0 h

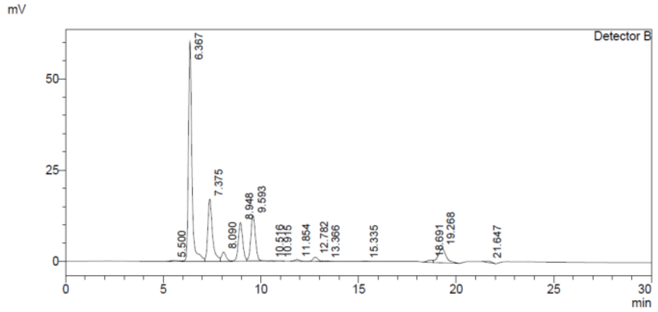

Detector B

Peak#	Ret. Time	Area	Height	Conc.
1	6.315	502605	32533	32.379
2	7.003	11697	1206	0.754
3	7.743	275103	12544	17.723
4	8.899	438432	28687	28.245
5	9.546	122975	7388	7.922
6	10.447	5015	352	0.323
7	11.728	3891	190	0.251
8	12.699	12288	679	0.792
9	18.639	38749	552	2.496
10	20.606	34827	547	2.244
11	21.625	17933	774	1.155
12	22.327	88736	682	5.717
Total		1552251	86134	

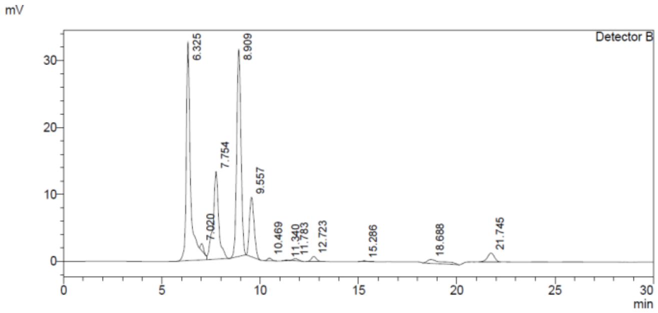

Detector B

Peak#	Ret. Time	Area	Height	Conc.
1	6.325	505347	32569	33.999
2	7.020	10787	1052	0.726
3	7.754	264120	13081	17.769
4	8.909	469159	30877	31.564
5	9.557	145309	8871	9.776
6	10.469	5972	412	0.402
7	11.340	895	74	0.060
8	11.783	5911	312	0.398
9	12.723	12850	735	0.865
10	15.286	2176	113	0.146
11	18.688	32828	567	2.209
12	21.745	31022	1302	2.087
Total		1486377	89966	

12 h

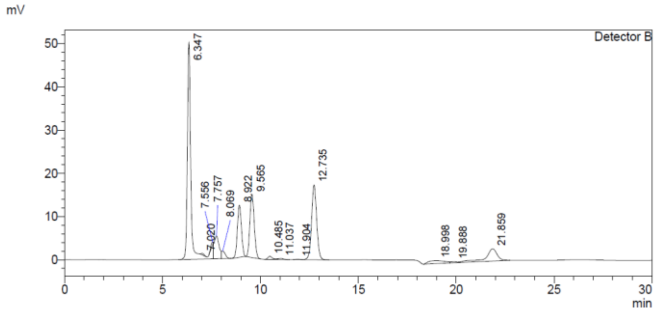

Detector B

Peak#	Ret. Time	Area	Height	Conc.
1	5.509	10467	304	0.453
2	6.378	1040648	87986	45.036
3	7.755	126749	3803	5.485
4	8.073	43706	2824	1.891
5	8.947	69057	4459	2.989
6	9.594	395619	25114	17.121
7	10.520	14064	937	0.609
8	10.925	7831	375	0.339
9	11.930	1466	113	0.063
10	12.778	478018	26534	20.687
11	19.278	123085	4137	5.327
Total		2310710	156586	

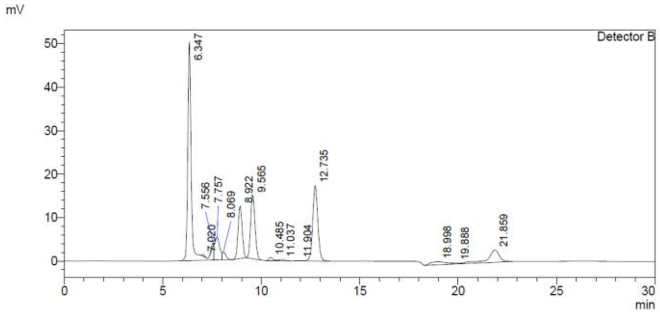

Detector B

Peak#	Ret. Time	Area	Height	Conc.
1	6.347	637823	50207	38.550
2	7.020	3624	328	0.219
3	7.556	8139	607	0.492
4	7.757	92990	5296	5.620
5	8.069	21686	1765	1.311
6	8.922	176614	12043	10.675
7	9.565	221375	14579	13.380
8	10.485	10774	712	0.651
9	11.037	5042	233	0.305
10	11.904	1245	92	0.075
11	12.735	315328	17310	19.058
12	18.998	38957	690	2.355
13	19.888	5259	236	0.318
14	21.859	115674	2850	6.991
Total		1654530	106949	

18 h

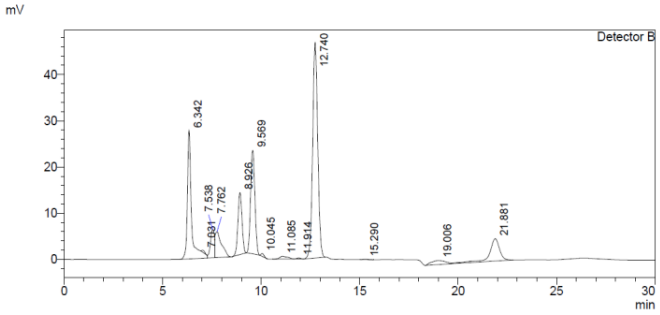

Detector B

Peak#	Ret. Time	Area	Height	Conc.
1	6.342	400690	27786	18.000
2	7.031	4434	415	0.199
3	7.538	88644	6144	3.982
4	7.762	122482	5623	5.502
5	8.926	194977	13408	8.759
6	9.569	331868	22396	14.908
7	10.045	6335	594	0.285
8	11.085	18373	639	0.825
9	11.914	3237	221	0.145
10	12.740	838808	46583	37.681
11	15.290	1603	87	0.072
12	19.006	43940	887	1.974
13	21.881	170677	4817	7.667
Total		2226066	129600	

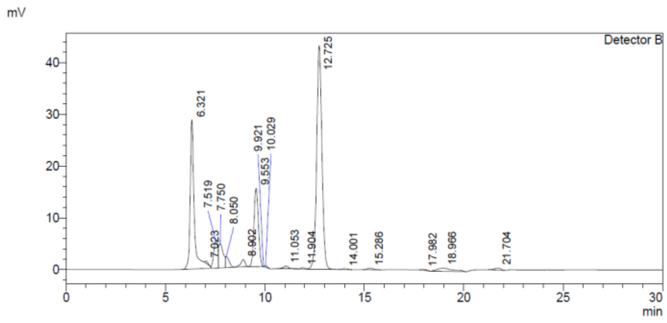

Detector B

Peak#	Ret. Time	Area	Height	Conc.
1	6.321	412608	28790	24.108
2	7.023	4293	403	0.251
3	7.519	73872	5278	4.316
4	7.750	78912	4651	4.611
5	8.050	25295	2099	1.478
6	8.902	21930	1411	1.281
7	9.553	231321	15135	13.516
8	9.921	0	-0	0.000
9	10.029	3171	290	0.185
10	11.053	11750	570	0.687
11	11.904	3978	263	0.232
12	12.725	792055	43146	46.278
13	14.001	2520	153	0.147
14	15.286	5677	291	0.332
15	17.982	2851	154	0.167
16	18.966	32726	636	1.912
17	21.704	8562	395	0.500
Total		1711520	103664	

24 h

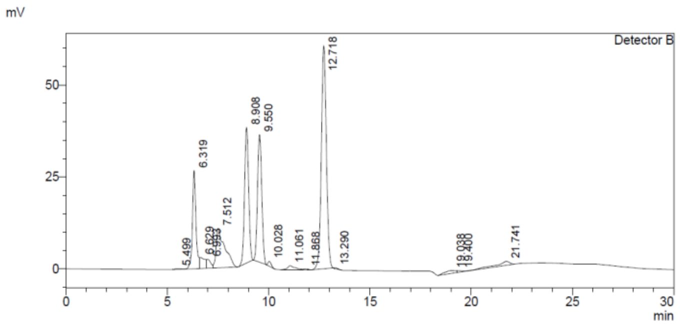

Detector B

Peak#	Ret. Time	Area	Height	Conc.
1	5.499	3382	195	0.115
2	6.319	321431	26719	10.968
3	6.629	55864	3007	1.906
4	6.993	31473	2356	1.074
5	7.512	313913	10680	10.712
6	8.908	519454	36820	17.726
7	9.550	492183	34640	16.795
8	10.028	14401	1313	0.491
9	11.061	27617	1098	0.942
10	11.868	3246	208	0.111
11	12.718	1055979	60558	36.034
12	13.290	3017	250	0.103
13	19.038	29336	761	1.001
14	19.400	7103	408	0.242
15	21.741	52146	1039	1.779
Total		2930544	180052	

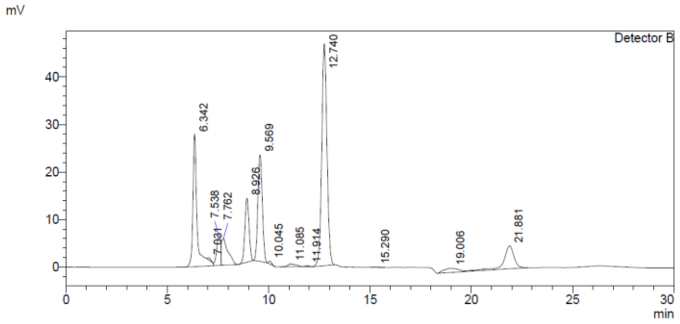

Detector B

Peak#	Ret. Time	Area	Height	Conc.
1	6.342	400690	27786	18.000
2	7.031	4434	415	0.199
3	7.538	88644	6144	3.982
4	7.762	122482	5623	5.502
5	8.926	194977	13408	8.759
6	9.569	331868	22396	14.908
7	10.045	6335	594	0.285
8	11.085	18373	639	0.825
9	11.914	3237	221	0.145
10	12.740	838808	46583	37.681
11	15.290	1603	87	0.072
12	19.006	43940	887	1.974
13	21.881	170677	4817	7.667
Total		2226066	129600	

36 h

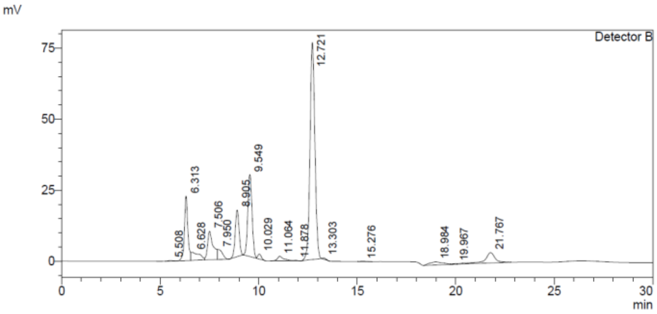

Detector B

Peak#	Ret. Time	Area	Height	Conc.
1	5.508	3518	190	0.123
2	6.313	277479	22742	9.718
3	6.628	80450	2879	2.817
4	7.506	205654	10044	7.202
5	7.950	57495	3509	2.014
6	8.905	227884	16393	7.981
7	9.549	416397	28829	14.583
8	10.029	21677	1776	0.759
9	11.064	39319	1639	1.377
10	11.878	3222	221	0.113
11	12.721	1330573	76374	46.598
12	13.303	4853	413	0.170
13	15.276	1531	85	0.054
14	18.984	49817	1076	1.745
15	19.967	3397	169	0.119
16	21.767	132144	3612	4.628
Total		2855410	169950	

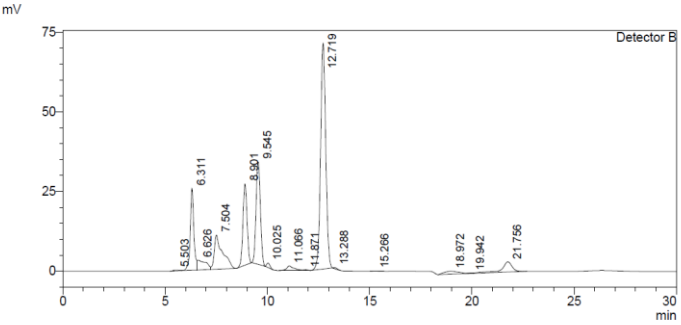

Detector B

Peak#	Ret. Time	Area	Height	Conc.
1	5.503	4406	224	0.146
2	6.311	315910	25656	10.491
3	6.626	87835	3070	2.917
4	7.504	303260	10672	10.071
5	8.901	360960	25456	11.987
6	9.545	463826	32139	15.403
7	10.025	15699	1405	0.521
8	11.066	36670	1433	1.218
9	11.871	3462	228	0.115
10	12.719	1256368	70797	41.721
11	13.288	4306	321	0.143
12	15.266	1215	70	0.040
13	18.972	43625	946	1.449
14	19.942	2380	122	0.079
15	21.756	111413	3172	3.700
Total		3011337	175710	

42 h

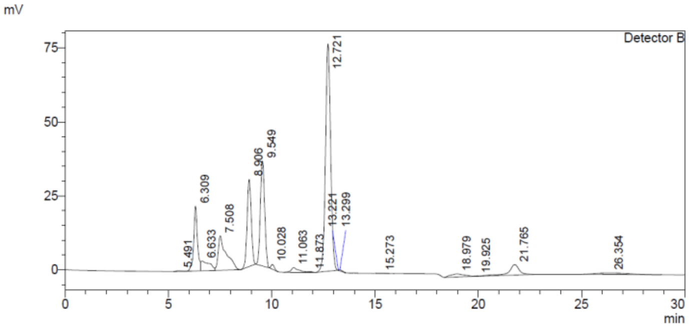

Detector B

Peak#	Ret. Time	Area	Height	Conc.
1	5.491	4455	245	0.137
2	6.309	277459	21888	8.529
3	6.633	95784	3260	2.945
4	7.508	320726	11635	9.860
5	8.906	412156	29431	12.670
6	9.549	505396	35459	15.537
7	10.028	17683	1597	0.544
8	11.063	40220	1591	1.236
9	11.873	3467	239	0.107
10	12.721	1340878	76744	41.220
11	13.221	3	-5	0.000
12	13.299	4562	330	0.140
13	15.273	1204	70	0.037
14	18.979	47292	1031	1.454
15	19.925	2252	118	0.069
16	21.765	127280	3559	3.913
17	26.354	52134	508	1.603
Total		3252949	187701	

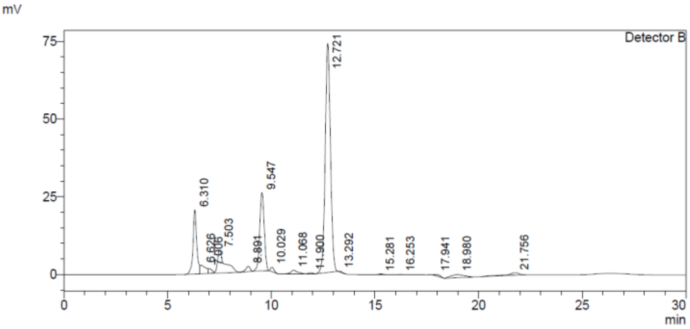

Detector B

Peak#	Ret. Time	Area	Height	Conc.
1	6.310	258615	20590	10.951
2	6.626	49631	2681	2.102
3	7.006	19838	1580	0.840
4	7.503	185760	7355	7.866
5	8.891	25460	1706	1.078
6	9.547	371819	25124	15.744
7	10.029	16336	1443	0.692
8	11.068	28805	1239	1.220
9	11.900	4855	358	0.206
10	12.721	1306073	73565	55.304
11	13.292	3803	288	0.161
12	15.281	3428	181	0.145
13	16.253	1017	47	0.043
14	17.941	9948	436	0.421
15	18.980	44698	1053	1.893
16	21.756	31526	759	1.335
Total		2361613	138405	

48 h

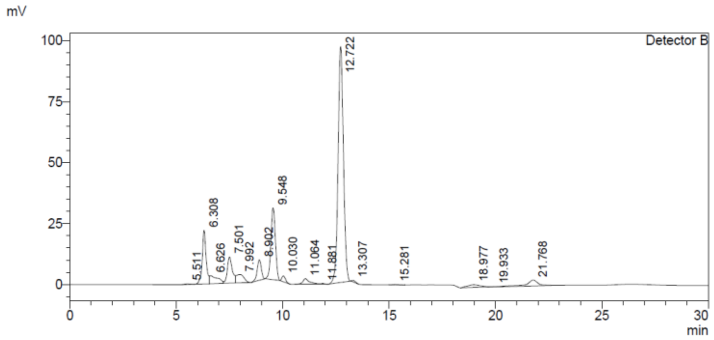

Detector B

Peak#	Ret. Time	Area	Height	Conc.
1	5.511	4705	236	0.152
2	6.308	278848	21917	8.989
3	6.626	87253	3268	2.813
4	7.501	177847	10744	5.733
5	7.992	84064	3411	2.710
6	8.902	114706	8385	3.698
7	9.548	425724	29355	13.724
8	10.030	27613	2363	0.890
9	11.064	51058	2248	1.646
10	11.881	3945	270	0.127
11	12.722	1692654	96641	54.565
12	13.307	7783	602	0.251
13	15.281	2456	136	0.079
14	18.977	47990	1118	1.547
15	19.933	2074	111	0.067
16	21.768	93359	2406	3.010
Total		3102078	183209	

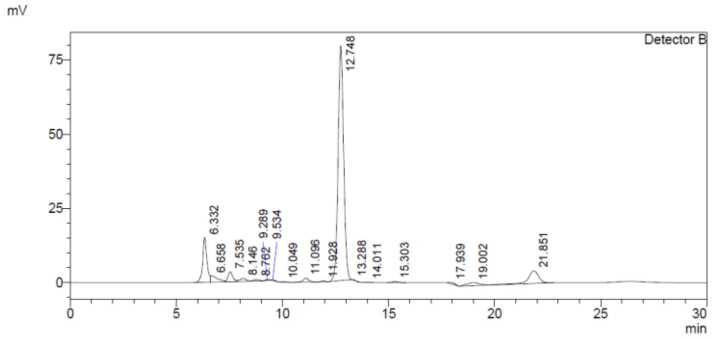

Detector B

Peak#	Ret. Time	Area	Height	Conc.
1	6.332	213083	15115	10.499
2	6.658	45044	2129	2.219
3	7.535	51110	3362	2.518
4	8.146	21312	1033	1.050
5	8.762	10689	522	0.527
6	9.289	3494	328	0.172
7	9.534	2869	236	0.141
8	10.049	2086	169	0.103
9	11.096	24748	1314	1.219
10	11.928	4903	363	0.242
11	12.748	1438321	79025	70.867
12	13.288	1148	36	0.057
13	14.011	1155	83	0.057
14	15.303	7268	373	0.358
15	17.939	8494	382	0.418
16	19.002	42384	1035	2.088
17	21.851	151496	4233	7.464
Total		2029604	109740	

60 h

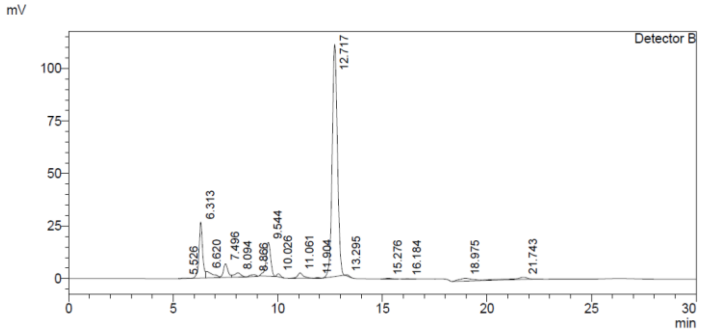

Detector B

Peak#	Ret. Time	Area	Height	Conc.
1	5.526	3579	175	0.122
2	6.313	322496	26621	10.979
3	6.620	67338	3003	2.292
4	7.496	97761	6489	3.328
5	8.094	45219	2036	1.539
6	8.866	17828	942	0.607
7	9.544	246544	16046	8.393
8	10.026	16238	1433	0.553
9	11.061	49015	2500	1.669
10	11.904	3585	286	0.122
11	12.717	1937506	110339	65.958
12	13.295	6983	554	0.238
13	15.276	5015	267	0.171
14	16.184	1196	62	0.041
15	18.975	57348	1312	1.952
16	21.743	59841	1035	2.037
Total		2937491	173101	

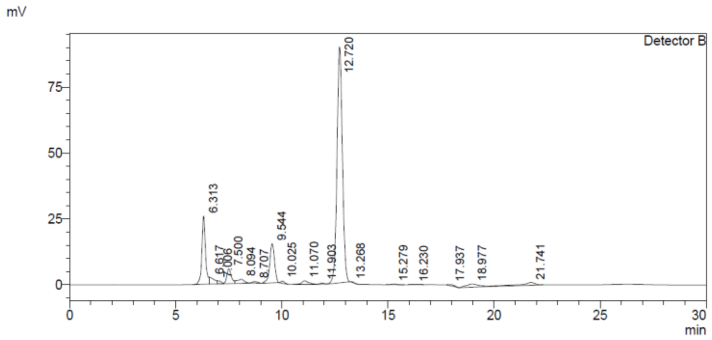

Detector B

Peak#	Ret. Time	Area	Height	Conc.
1	6.313	311687	25885	12.563
2	6.617	41114	2540	1.657
3	7.006	14545	1192	0.586
4	7.500	81918	5582	3.302
5	8.094	36517	1487	1.472
6	8.707	8922	519	0.360
7	9.544	226731	14788	9.139
8	10.025	9422	825	0.380
9	11.070	30357	1366	1.224
10	11.903	5275	396	0.213
11	12.720	1593393	89443	64.225
12	13.268	1741	67	0.070
13	15.279	4413	229	0.178
14	16.230	1234	57	0.050
15	17.937	8620	378	0.347
16	18.977	49018	1156	1.976
17	21.741	56030	1032	2.258
Total		2480937	146942	

66 h

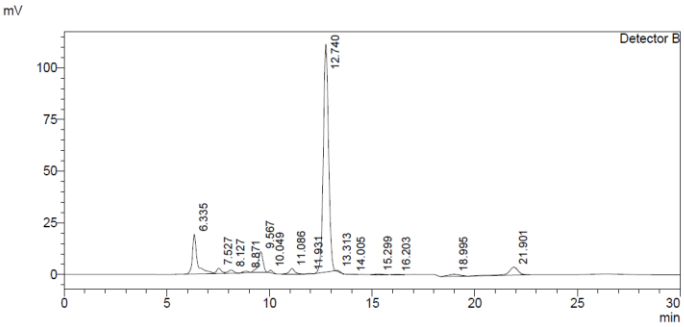

Detector B

Peak#	Ret. Time	Area	Height	Conc.
1	6.335	321013	19070	11.440
2	7.527	42480	2551	1.514
3	8.127	31700	1627	1.130
4	8.871	14087	705	0.502
5	9.567	167030	10045	5.952
6	10.049	16584	1311	0.591
7	11.086	48931	2604	1.744
8	11.931	3509	275	0.125
9	12.740	1972766	110066	70.303
10	13.313	7310	507	0.261
11	14.005	1084	81	0.039
12	15.299	6505	335	0.232
13	16.203	1374	73	0.049
14	18.995	39458	1026	1.406
15	21.901	132241	3872	4.713
Total		2806071	154146	

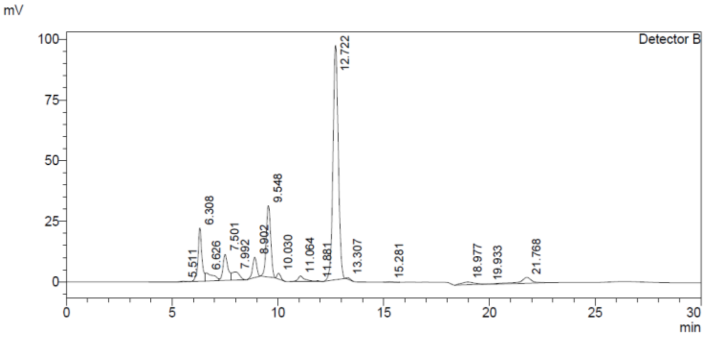

Detector B

Peak#	Ret. Time	Area	Height	Conc.
1	5.511	4705	236	0.152
2	6.308	278848	21917	8.989
3	6.626	87253	3268	2.813
4	7.501	177847	10744	5.733
5	7.992	84064	3411	2.710
6	8.902	114706	8385	3.698
7	9.548	425724	29355	13.724
8	10.030	27613	2363	0.890
9	11.064	51058	2248	1.646
10	11.881	3945	270	0.127
11	12.722	1692654	96641	54.565
12	13.307	7783	602	0.251
13	15.281	2456	136	0.079
14	18.977	47990	1118	1.547
15	19.933	2074	111	0.067
16	21.768	93359	2406	3.010
Total		3102078	183209	

72 h

Detector B

Peak#	Ret. Time	Area	Height	Conc.
1	5.521	6094	292	0.168
2	6.305	306250	23393	8.458
3	6.623	102463	3901	2.830
4	7.495	193260	12060	5.337
5	8.010	94017	3616	2.596
6	8.895	67814	4880	1.873
7	9.544	458153	30976	12.653
8	10.025	26052	2254	0.719
9	11.059	63550	2922	1.755
10	11.884	4643	323	0.128
11	12.717	2128535	121298	58.783
12	13.299	11203	875	0.309
13	15.278	3555	189	0.098
14	18.973	56303	1348	1.555
15	19.950	3314	163	0.092
16	21.727	43225	600	1.194
17	22.356	5373	177	0.148
18	26.291	47217	468	1.304
Total		3621022	209735	

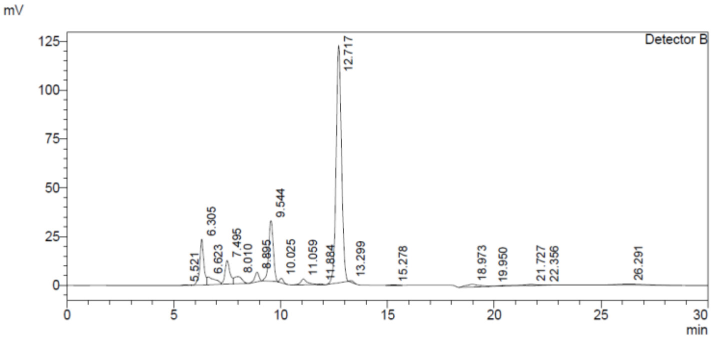

Detector B

Peak#	Ret. Time	Area	Height	Conc.
1	5.528	4256	204	0.136
2	6.311	314234	25292	10.021
3	6.620	82570	3335	2.633
4	7.495	139818	8999	4.459
5	8.066	67477	2653	2.152
6	8.886	26953	1758	0.860
7	9.544	322738	21232	10.292
8	10.025	20255	1742	0.646
9	11.061	53669	2588	1.712
10	11.895	4010	299	0.128
11	12.717	1957949	111488	62.441
12	13.295	9069	671	0.289
13	15.273	4020	215	0.128
14	16.195	1066	57	0.034
15	18.987	61884	1373	1.974
16	21.736	65728	953	2.096
Total		3135695	182859	

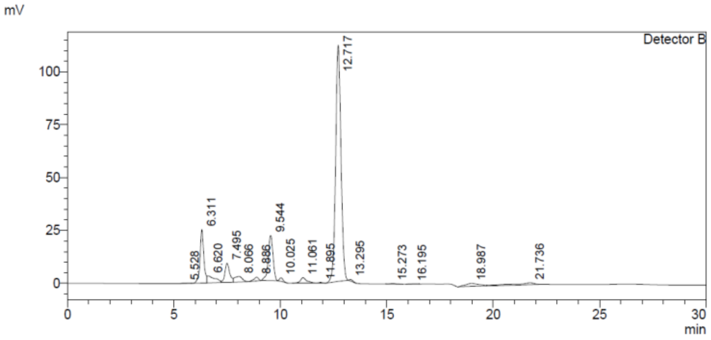

84 h

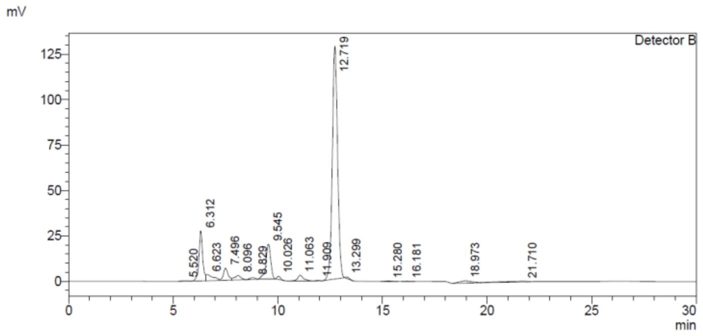

Detector B

Peak#	Ret. Time	Area	Height	Conc.
1	5.520	5788	268	0.174
2	6.312	340921	27489	10.226
3	6.623	77657	3420	2.329
4	7.496	102339	6605	3.070
5	8.096	51412	2378	1.542
6	8.829	17271	832	0.518
7	9.545	299152	19046	8.973
8	10.026	20972	1794	0.629
9	11.063	61584	3140	1.847
10	11.909	3375	275	0.101
11	12.719	2251534	127897	67.534
12	13.299	10237	813	0.307
13	15.280	4723	250	0.142
14	16.181	1294	73	0.039
15	18.973	49079	1244	1.472
16	21.710	36596	457	1.098
Total		3333935	195982	

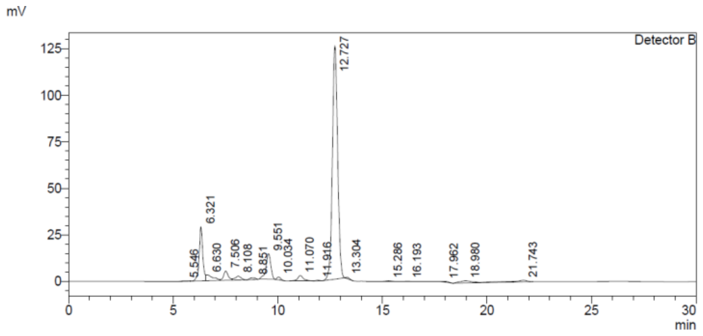

Detector B

Peak#	Ret. Time	Area	Height	Conc.
1	5.546	4001	186	0.126
2	6.321	351123	28936	11.070
3	6.630	71196	3205	2.245
4	7.506	76063	4964	2.398
5	8.108	41514	2080	1.309
6	8.851	16780	815	0.529
7	9.551	215022	13682	6.779
8	10.034	18299	1535	0.577
9	11.070	55688	2923	1.756
10	11.916	4074	322	0.128
11	12.727	2205593	125133	69.537
12	13.304	7998	656	0.252
13	15.286	6157	324	0.194
14	16.193	1399	75	0.044
15	17.962	7619	352	0.240
16	18.980	44409	1144	1.400
17	21.743	44882	824	1.415
Total		3171817	187157	

90 h

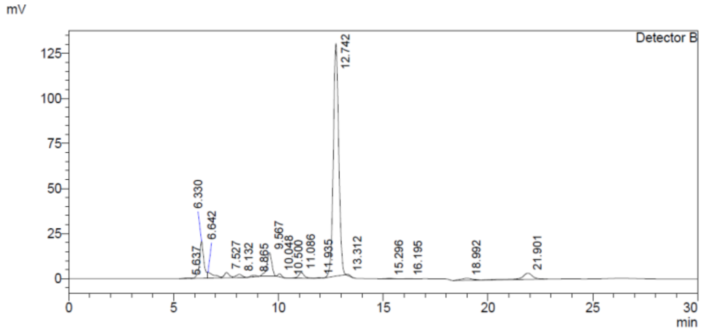

Detector B

Peak#	Ret. Time	Area	Height	Conc.
1	5.637	5399	224	0.165
2	6.330	295491	20454	9.043
3	6.642	66241	3151	2.027
4	7.527	49817	2956	1.525
5	8.132	36663	1806	1.122
6	8.865	15631	779	0.478
7	9.567	211750	12941	6.481
8	10.048	19532	1638	0.598
9	10.500	9	4	0.000
10	11.086	59966	3180	1.835
11	11.935	3292	261	0.101
12	12.742	2325091	128763	71.159
13	13.312	8629	655	0.264
14	15.296	5422	281	0.166
15	16.195	1517	82	0.046
16	18.992	42610	1141	1.304
17	21.901	120381	3440	3.684
Total		3267439	181757	

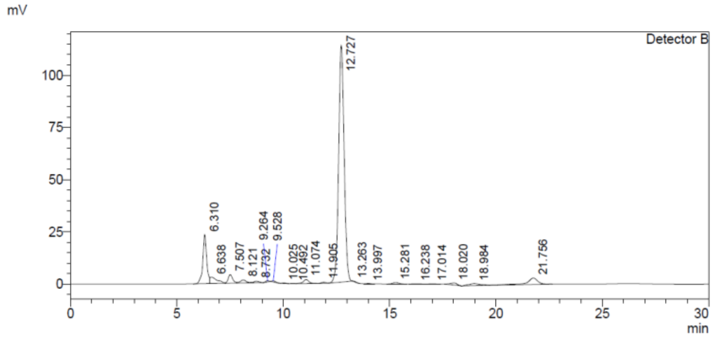

Detector B

Peak#	Ret. Time	Area	Height	Conc.
1	6.310	304389	23500	10.955
2	6.638	68041	3072	2.449
3	7.507	60125	4171	2.164
4	8.121	28688	1457	1.033
5	8.732	13114	691	0.472
6	9.264	5923	544	0.213
7	9.528	5351	463	0.193
8	10.025	3070	256	0.111
9	10.492	132	13	0.005
10	11.074	35967	1990	1.294
11	11.905	7907	580	0.285
12	12.727	2049582	113349	73.765
13	13.263	2511	76	0.090
14	13.997	2234	156	0.080
15	15.281	13581	691	0.489
16	16.238	2439	98	0.088
17	17.014	1781	86	0.064
18	18.020	21949	1004	0.790
19	18.984	39571	993	1.424
20	21.756	112162	3084	4.037
Total		2778520	156274	

Figure S2 LA fermentation on 8% (w/w) NH_3-H_2O_2 pretreated and unwashed corncob

A: The curves of fermentation on corncob substrate by NH_3 pretreatment for 1 day followed by H_2O_2 treatment for 1 day at pH6.0 adjusted by automatic feeding of NaOH solution.

0 h

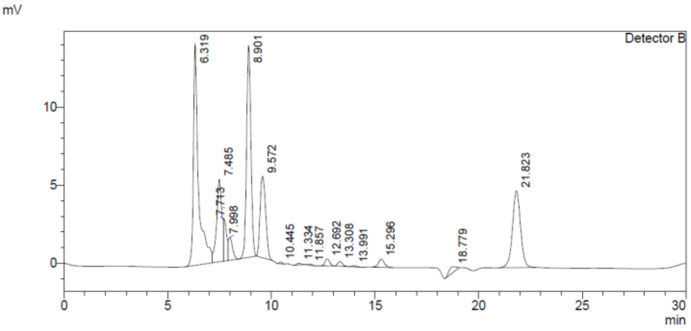

Detector B

Peak#	Ret. Time	Area	Height	Conc.
1	6.323	168100	11836	23.663
2	6.725	33721	1779	4.747
3	7.474	122882	5159	17.298
4	8.000	17664	1252	2.487
5	8.903	148718	10194	20.935
6	9.571	76619	4263	10.785
7	10.447	854	88	0.120
8	12.694	5822	378	0.820
9	13.309	12681	756	1.785
10	15.289	7034	389	0.990
11	18.811	39465	803	5.555
12	21.825	76829	2898	10.815
Total		710390	39795	

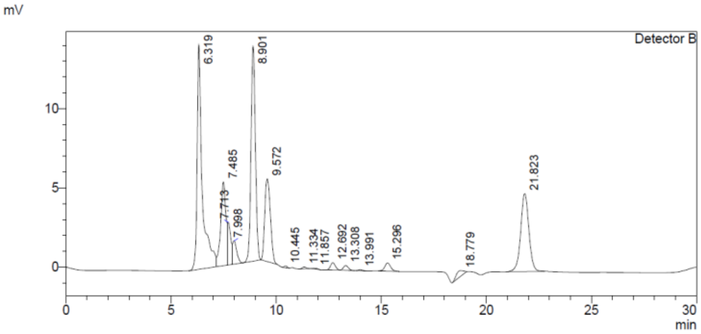

Detector B

Peak#	Ret. Time	Area	Height	Conc.
1	6.319	241738	14158	28.630
2	7.485	90671	5290	10.738
3	7.713	29415	2698	3.484
4	7.998	20334	1475	2.408
5	8.901	199230	13557	23.595
6	9.572	95354	5226	11.293
7	10.445	1077	109	0.128
8	11.334	1515	110	0.179
9	11.857	958	69	0.113
10	12.692	7134	453	0.845
11	13.308	4852	299	0.575
12	13.991	1103	73	0.131
13	15.296	10257	520	1.215
14	18.779	9150	347	1.084
15	21.823	131572	4913	15.582
Total		844358	49296	

12 h

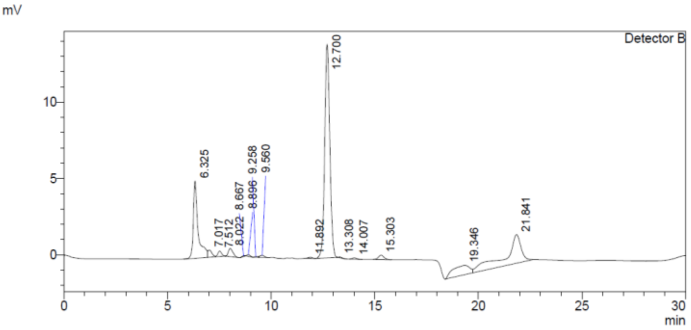

Detector B

Peak#	Ret. Time	Area	Height	Conc.
1	6.325	80561	5038	16.388
2	7.017	6403	466	1.303
3	7.512	4394	349	0.894
4	8.022	8087	527	1.645
5	8.667	414	51	0.084
6	8.896	1045	107	0.213
7	9.258	618	52	0.126
8	9.560	2038	141	0.415
9	11.892	1003	75	0.204
10	12.700	240875	13968	49.000
11	13.308	587	47	0.119
12	14.007	1435	95	0.292
13	15.303	5516	291	1.122
14	19.346	36248	603	7.374
15	21.841	102354	1864	20.821
Total		491579	23674	

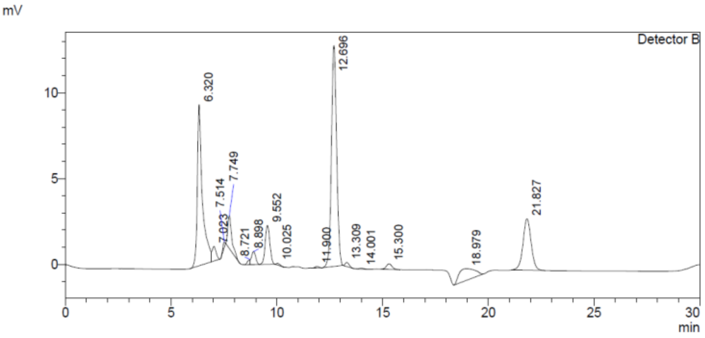

Detector B

Peak#	Ret. Time	Area	Height	Conc.
1	6.320	156885	9376	26.532
2	7.023	11287	848	1.909
3	7.514	2733	305	0.462
4	7.749	25604	1892	4.330
5	8.721	2406	274	0.407
6	8.898	10804	768	1.827
7	9.552	33875	2261	5.729
8	10.025	1346	103	0.228
9	11.900	1111	87	0.188
10	12.696	220846	12855	37.349
11	13.309	3082	234	0.521
12	14.001	792	55	0.134
13	15.300	6252	330	1.057
14	18.979	35146	675	5.944
15	21.827	79135	2986	13.383
Total		591306	33049	

18 h

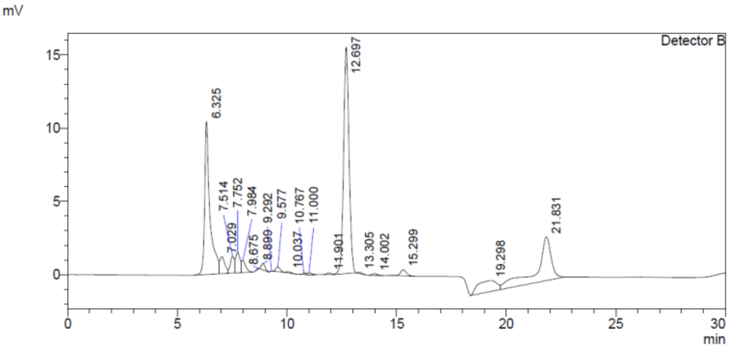

Detector B

Peak#	Ret. Time	Area	Height	Conc.
1	6.325	173674	10424	24.275
2	7.029	19017	1165	2.658
3	7.514	14621	1149	2.044
4	7.752	20386	1388	2.849
5	7.984	10155	804	1.419
6	8.675	448	51	0.063
7	8.899	4947	425	0.691
8	9.292	99	14	0.014
9	9.577	4136	301	0.578
10	10.037	1202	77	0.168
11	10.767	882	66	0.123
12	11.000	1383	102	0.193
13	11.901	1266	99	0.177
14	12.697	266600	15455	37.264
15	13.305	1186	100	0.166
16	14.002	1999	129	0.279
17	15.299	7873	415	1.100
18	19.298	45903	736	6.416
19	21.831	139658	2978	19.521
Total		715435	35878	

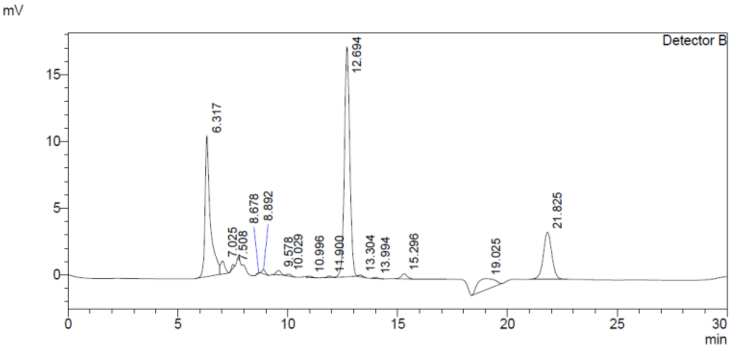

Detector B

Peak#	Ret. Time	Area	Height	Conc.
1	6.317	173827	10532	26.766
2	7.025	13664	981	2.104
3	7.508	2404	281	0.370
4	8.678	696	77	0.107
5	8.892	3317	300	0.511
6	9.578	4818	342	0.742
7	10.029	2072	146	0.319
8	10.996	1777	125	0.274
9	11.900	1528	118	0.235
10	12.694	296696	17211	45.686
11	13.304	1416	120	0.218
12	13.994	1051	73	0.162
13	15.296	7338	384	1.130
14	19.025	45171	843	6.956
15	21.825	93646	3518	14.420
Total		649423	35053	

24 h

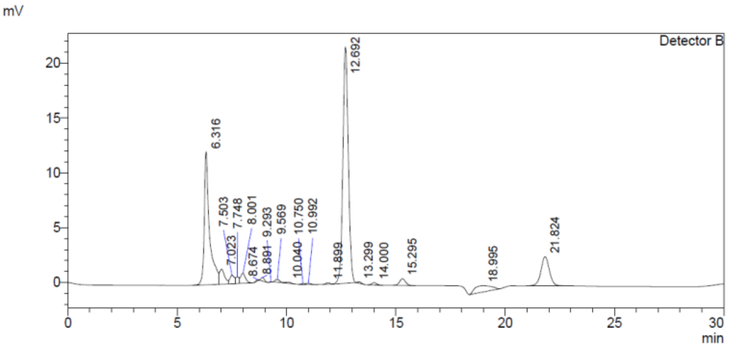

Detector B

Peak#	Ret. Time	Area	Height	Conc.
1	6.316	203426	12120	26.681
2	7.023	22328	1382	2.928
3	7.503	11175	780	1.466
4	7.748	7075	601	0.928
5	8.001	14281	924	1.873
6	8.674	766	83	0.101
7	8.891	3388	311	0.444
8	9.293	258	28	0.034
9	9.569	3562	257	0.467
10	10.040	1782	103	0.234
11	10.750	1088	81	0.143
12	10.992	1334	103	0.175
13	11.899	1720	134	0.226
14	12.692	372469	21521	48.853
15	13.299	1649	138	0.216
16	14.000	3487	221	0.457
17	15.295	11947	622	1.567
18	18.995	29970	554	3.931
19	21.824	70722	2658	9.276
Total		762429	42622	

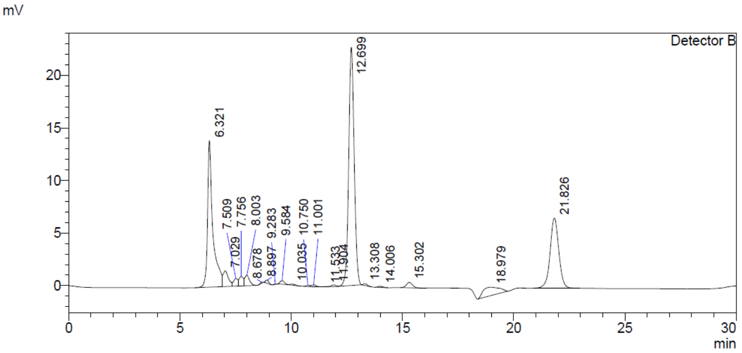

Detector B

Peak#	Ret. Time	Area	Height	Conc.
1	6.321	230366	13934	24.706
2	7.029	25186	1505	2.701
3	7.509	9628	730	1.033
4	7.756	12481	908	1.338
5	8.003	14906	999	1.599
6	8.678	820	92	0.088
7	8.897	2937	282	0.315
8	9.283	220	29	0.024
9	9.584	4420	333	0.474
10	10.035	1610	108	0.173
11	10.750	1109	90	0.119
12	11.001	2250	158	0.241
13	11.533	132	14	0.014
14	11.904	2022	154	0.217
15	12.699	390656	22670	41.896
16	13.308	2140	176	0.229
17	14.006	1674	110	0.180
18	15.302	9659	507	1.036
19	18.979	41633	803	4.465
20	21.826	178597	6666	19.154
Total		932446	50268	

36 h

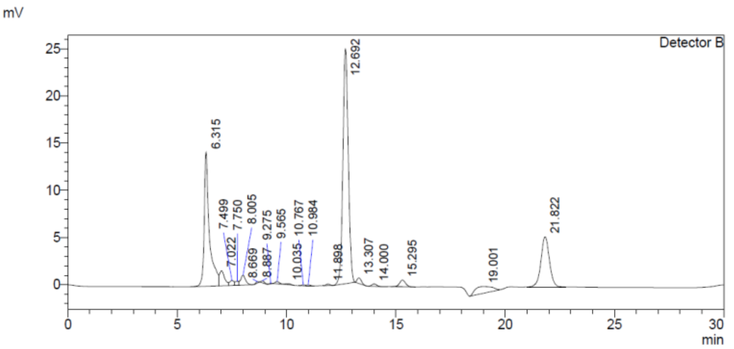

Detector B

Peak#	Ret. Time	Area	Height	Conc.
1	6.315	234254	14192	25.044
2	7.022	26387	1602	2.821
3	7.499	7652	528	0.818
4	7.750	4918	447	0.526
5	8.005	16385	1054	1.752
6	8.669	1254	141	0.134
7	8.887	2328	225	0.249
8	9.275	342	43	0.037
9	9.565	2683	216	0.287
10	10.035	1644	99	0.176
11	10.767	1128	93	0.121
12	10.984	1245	95	0.133
13	11.898	2064	159	0.221
14	12.692	427201	24850	45.671
15	13.307	8051	597	0.861
16	14.000	3889	251	0.416
17	15.295	13876	715	1.484
18	19.001	37367	708	3.995
19	21.822	142710	5321	15.257
Total		935378	51338	

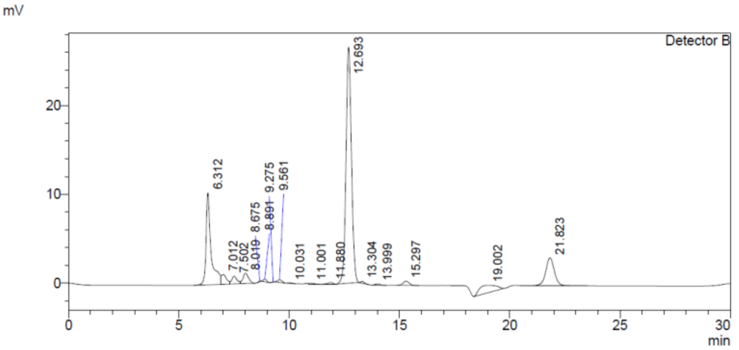

Detector B

Peak#	Ret. Time	Area	Height	Conc.
1	6.312	164897	10353	19.843
2	7.012	17848	1119	2.148
3	7.502	14598	880	1.757
4	8.019	20577	1172	2.476
5	8.675	580	64	0.070
6	8.891	3332	311	0.401
7	9.275	302	36	0.036
8	9.561	4061	295	0.489
9	10.031	712	53	0.086
10	11.001	1749	116	0.211
11	11.880	2987	180	0.359
12	12.693	457563	26574	55.061
13	13.304	2370	195	0.285
14	13.999	1805	121	0.217
15	15.297	9089	475	1.094
16	19.002	45367	856	5.459
17	21.823	83179	3114	10.009
Total		831017	45912	

42 h

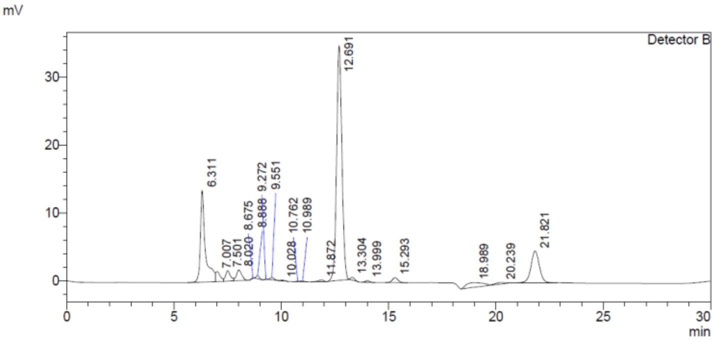

Detector B

Peak#	Ret. Time	Area	Height	Conc.
1	6.311	217932	13510	19.847
2	7.007	22135	1472	2.016
3	7.501	24534	1537	2.234
4	8.020	27162	1577	2.474
5	8.675	710	74	0.065
6	8.888	5069	463	0.462
7	9.272	416	53	0.038
8	9.551	4005	317	0.365
9	10.028	1157	80	0.105
10	10.762	1155	98	0.105
11	10.989	1285	107	0.117
12	11.872	4117	227	0.375
13	12.691	596073	34526	54.284
14	13.304	5339	418	0.486
15	13.999	3770	243	0.343
16	15.293	14199	733	1.293
17	18.989	36050	661	3.283
18	20.239	7053	206	0.642
19	21.821	125894	4683	11.465
Total		1098057	60985	

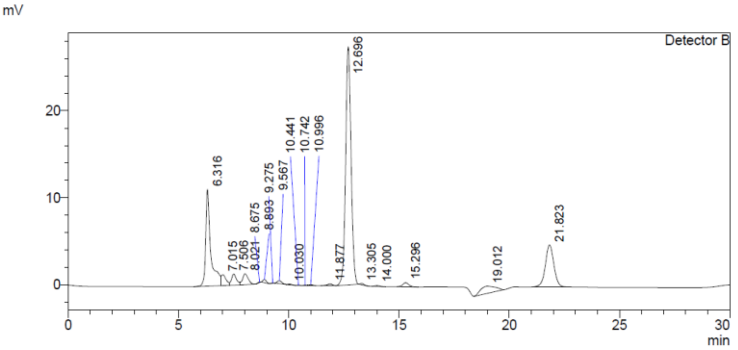

Detector B

Peak#	Ret. Time	Area	Height	Conc.
1	6.316	177250	11086	19.560
2	7.015	19448	1250	2.146
3	7.506	20206	1279	2.230
4	8.021	22296	1251	2.460
5	8.675	531	59	0.059
6	8.893	3868	363	0.427
7	9.275	323	41	0.036
8	9.567	4354	325	0.480
9	10.030	930	69	0.103
10	10.441	233	29	0.026
11	10.742	718	62	0.079
12	10.996	1428	116	0.158
13	11.877	3346	193	0.369
14	12.696	471548	27327	52.037
15	13.305	1751	149	0.193
16	14.000	1770	118	0.195
17	15.296	9192	481	1.014
18	19.012	38479	815	4.246
19	21.823	128499	4818	14.180
Total		906170	49831	

48 h

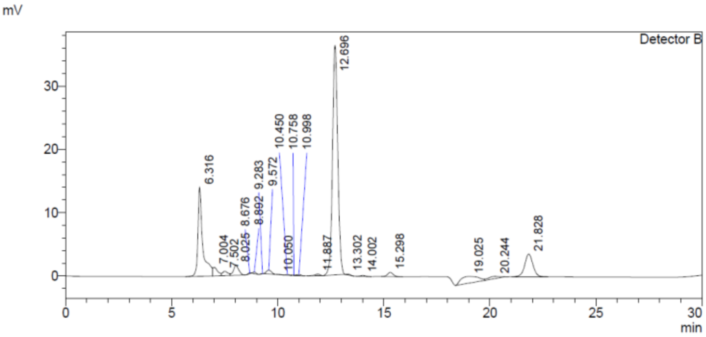

Detector B

Peak#	Ret. Time	Area	Height	Conc.
1	6.316	221914	14054	19.865
2	7.004	21227	1336	1.900
3	7.502	12301	693	1.101
4	8.025	26828	1563	2.401
5	8.676	1063	120	0.095
6	8.892	2454	246	0.220
7	9.283	321	32	0.029
8	9.572	9385	583	0.840
9	10.050	542	50	0.048
10	10.450	1275	117	0.114
11	10.758	886	79	0.079
12	10.998	2057	159	0.184
13	11.887	4224	244	0.378
14	12.696	628864	36283	56.293
15	13.302	1539	135	0.138
16	14.002	2414	160	0.216
17	15.298	12805	666	1.146
18	19.025	57908	1033	5.184
19	20.244	13247	341	1.186
20	21.828	95878	3585	8.583
Total		1117131	61479	

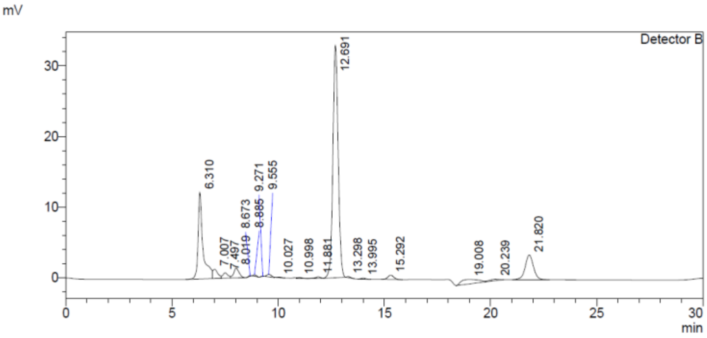

Detector B

Peak#	Ret. Time	Area	Height	Conc.
1	6.310	196652	12255	19.864
2	7.007	20865	1303	2.108
3	7.497	13148	775	1.328
4	8.019	24462	1398	2.471
5	8.673	860	96	0.087
6	8.885	2262	227	0.228
7	9.271	426	49	0.043
8	9.555	5150	357	0.520
9	10.027	943	68	0.095
10	10.998	2000	145	0.202
11	11.881	3545	216	0.358
12	12.691	568963	32853	57.471
13	13.298	1520	133	0.154
14	13.995	2203	146	0.223
15	15.292	11673	604	1.179
16	19.008	35262	635	3.562
17	20.239	7182	211	0.725
18	21.820	92881	3474	9.382
Total		989998	54946	

B: The curves of fermentation on corncob substrate by NH_3 pretreatment for 1 day followed by H_2O_2 treatment for 7 day at pH6.0 adjusted by automatic feeding of NaOH solution.

0 h

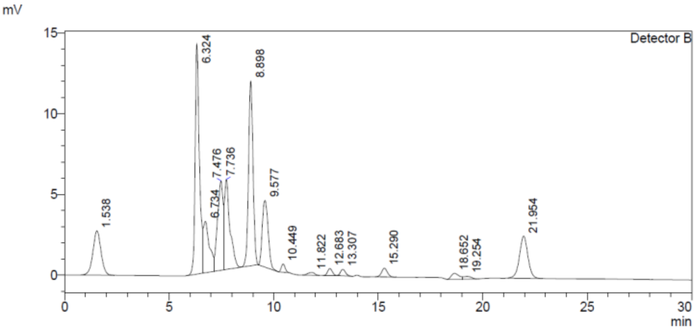

Detector B

Peak#	Ret. Time	Area	Height	Conc.
1	6.335	133956	9965	18.850
2	6.750	39230	2002	5.521
3	7.499	52554	3205	7.395
4	7.751	62891	3368	8.850
5	8.911	103145	6979	14.515
6	9.584	54432	2911	7.660
7	10.464	4526	362	0.637
8	11.833	2898	128	0.408
9	12.696	4907	311	0.691
10	13.317	4376	268	0.616
11	15.302	6499	335	0.915
12	19.322	37992	697	5.346
13	21.866	203217	5011	28.597
Total		710625	35542	

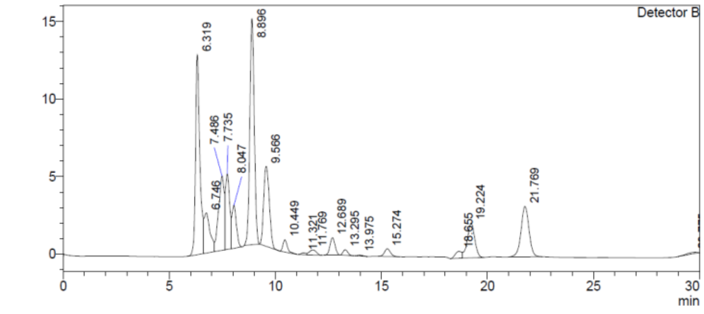

Detector B

Peak#	Ret. Time	Area	Height	Conc.
1	6.319	184772	12888	19.544
2	6.746	49162	2594	5.200
3	7.486	86523	4794	9.152
4	7.735	70722	4870	7.480
5	8.047	39592	2778	4.188
6	8.896	215032	14564	22.744
7	9.566	93039	5160	9.841
8	10.449	11412	782	1.207
9	11.321	1780	121	0.188
10	11.769	7256	319	0.768
11	12.689	18249	1107	1.930
12	13.295	5653	355	0.598
13	13.975	1292	84	0.137
14	15.274	9929	499	1.050
15	18.655	9557	445	1.011
16	19.224	54312	2240	5.745
17	21.769	83999	3247	8.885
18	29.775	3145	78	0.333
Total		945426	56925	

12 h

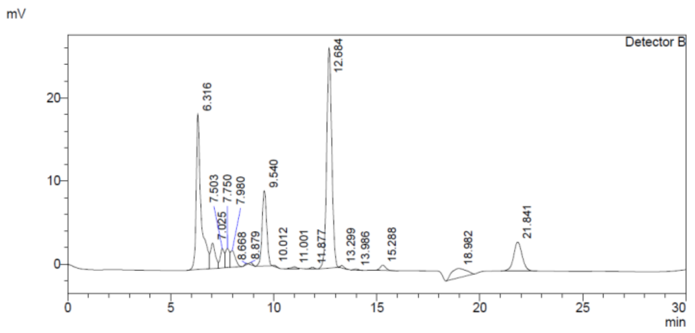

Detector B

Peak#	Ret. Time	Area	Height	Conc.
1	6.316	308408	18722	24.962
2	7.025	51521	3066	4.170
3	7.503	30812	2319	2.494
4	7.750	32924	2308	2.665
5	7.980	32574	2026	2.636
6	8.668	1224	139	0.099
7	8.879	2703	271	0.219
8	9.540	137332	9056	11.115
9	10.012	1564	129	0.127
10	11.001	5305	257	0.429
11	11.877	2868	195	0.232
12	12.684	456859	26472	36.978
13	13.299	3612	286	0.292
14	13.986	1688	114	0.137
15	15.288	11800	616	0.955
16	18.982	55499	1126	4.492
17	21.841	98812	3479	7.998
Total		1235504	70581	

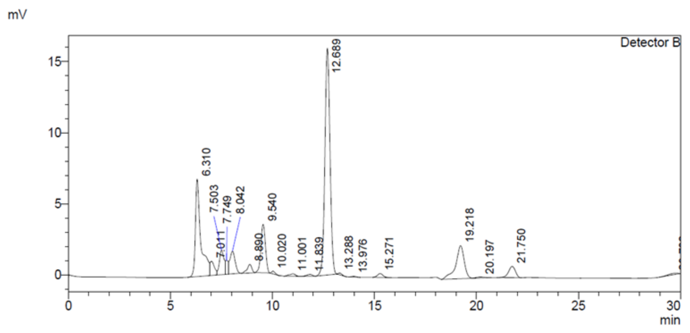

Detector B

Peak#	Ret. Time	Area	Height	Conc.
1	6.310	126512	6845	19.406
2	7.011	15321	992	2.350
3	7.503	27228	1728	4.177
4	7.749	8981	969	1.378
5	8.042	28112	1594	4.312
6	8.890	9519	599	1.460
7	9.540	51645	3402	7.922
8	10.020	2243	184	0.344
9	11.001	3568	168	0.547
10	11.839	2508	126	0.385
11	12.689	278962	15925	42.791
12	13.288	1395	119	0.214
13	13.976	871	56	0.134
14	15.271	5237	274	0.803
15	19.218	66423	2324	10.189
16	20.197	1901	73	0.292
17	21.750	18372	795	2.818
18	29.782	3115	76	0.478
Total		651913	36249	

18 h

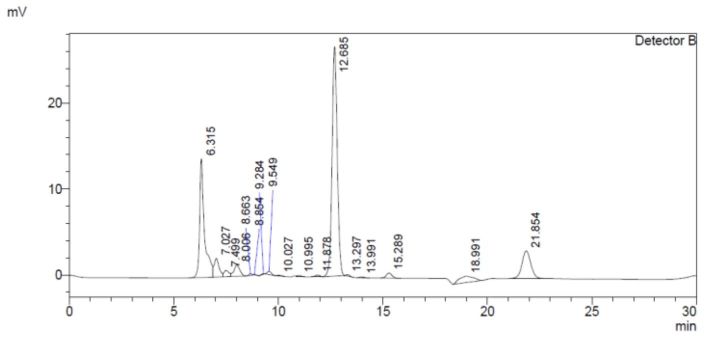

Detector B

Peak#	Ret. Time	Area	Height	Conc.
1	6.315	224211	13830	24.422
2	7.027	35735	2168	3.893
3	7.499	12879	753	1.403
4	8.006	26101	1341	2.843
5	8.663	2104	205	0.229
6	8.854	115	17	0.013
7	9.284	862	78	0.094
8	9.549	4750	355	0.517
9	10.027	1244	86	0.135
10	10.995	1928	148	0.210
11	11.878	2997	205	0.326
12	12.685	461324	26691	50.250
13	13.297	1981	166	0.216
14	13.991	1738	117	0.189
15	15.289	11574	603	1.261
16	18.991	34718	726	3.782
17	21.854	93794	3216	10.217
Total		918055	50704	

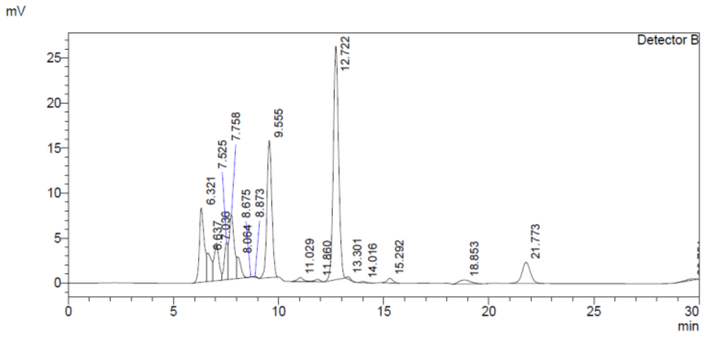

Detector B

Peak#	Ret. Time	Area	Height	Conc.
1	6.313	227888	13164	19.753
2	7.015	31330	1922	2.716
3	7.512	56751	3979	4.919
4	7.737	58009	3930	5.028
5	8.042	47333	3062	4.103
6	8.729	654	14	0.057
7	8.882	4173	365	0.362
8	9.540	176368	11473	15.288
9	10.020	936	86	0.081
10	10.450	7142	582	0.619
11	10.992	514	50	0.045
12	11.795	5884	275	0.510
13	12.690	378456	21656	32.804
14	13.289	3059	230	0.265
15	13.979	1038	69	0.090
16	15.274	7394	380	0.641
17	18.019	2056	123	0.178
18	19.217	69527	2315	6.027
19	21.769	72334	2803	6.270
20	29.805	2830	68	0.245
Total		1153675	66546	

24 h

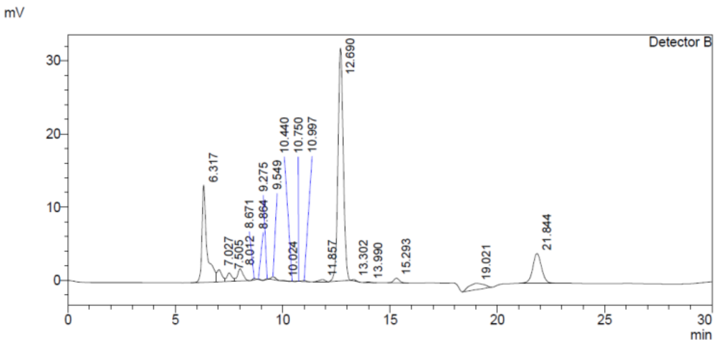

Detector B

Peak#	Ret. Time	Area	Height	Conc.
1	6.317	213532	13244	20.739
2	7.027	27147	1656	2.637
3	7.505	19083	1159	1.853
4	8.012	29011	1647	2.818
5	8.671	1667	174	0.162
6	8.864	509	53	0.049
7	9.275	960	92	0.093
8	9.549	5385	387	0.523
9	10.024	765	59	0.074
10	10.440	485	54	0.047
11	10.750	801	72	0.078
12	10.997	2024	159	0.197
13	11.857	5675	294	0.551
14	12.690	549100	31756	53.330
15	13.302	2339	193	0.227
16	13.990	1641	112	0.159
17	15.293	12296	641	1.194
18	19.021	41813	848	4.061
19	21.844	115401	4070	11.208
Total		1029634	56672	

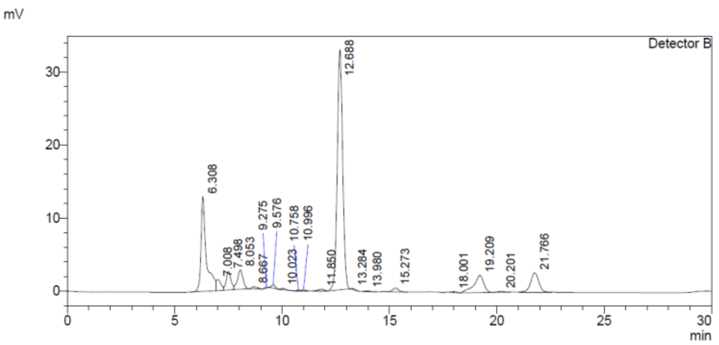

Detector B

Peak#	Ret. Time	Area	Height	Conc.
1	6.308	224027	12962	20.529
2	7.008	22624	1497	2.073
3	7.498	39749	2541	3.642
4	8.053	47111	2658	4.317
5	8.667	5450	279	0.499
6	9.275	1178	118	0.108
7	9.576	5201	417	0.477
8	10.023	2957	204	0.271
9	10.758	1212	97	0.111
10	10.996	1707	134	0.156
11	11.850	4491	235	0.412
12	12.688	576276	32856	52.807
13	13.284	2092	170	0.192
14	13.980	1676	112	0.154
15	15.273	10420	536	0.955
16	18.001	1225	79	0.112
17	19.209	74188	2354	6.798
18	20.201	2013	85	0.184
19	21.766	67688	2635	6.203
Total		1091287	59969	

36 h

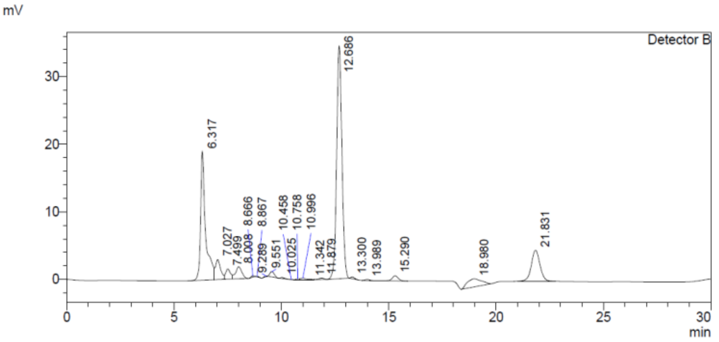

Detector B

Peak#	Ret. Time	Area	Height	Conc.
1	6.317	307762	19062	24.813
2	7.027	49390	2977	3.982
3	7.499	24977	1526	2.014
4	8.008	35584	1790	2.869
5	8.666	2133	229	0.172
6	8.867	919	94	0.074
7	9.289	1000	82	0.081
8	9.551	10926	786	0.881
9	10.025	2158	159	0.174
10	10.458	53	10	0.004
11	10.758	1149	109	0.093
12	10.996	3120	211	0.252
13	11.342	748	56	0.060
14	11.879	3948	259	0.318
15	12.686	594491	34432	47.931
16	13.300	2945	243	0.237
17	13.989	2302	154	0.186
18	15.290	14649	762	1.181
19	18.980	54739	1187	4.413
20	21.831	127319	4594	10.265
Total		1240312	68722	

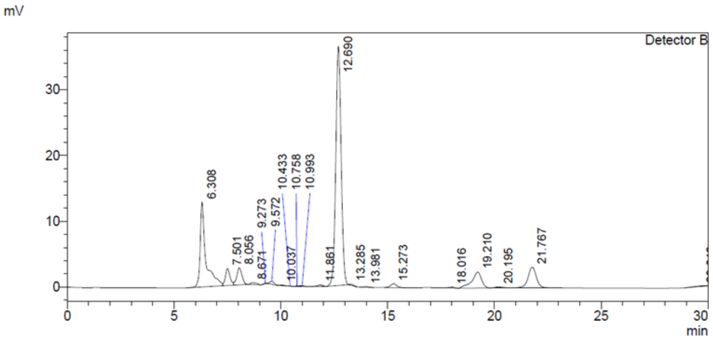

Detector B

Peak#	Ret. Time	Area	Height	Conc.
1	6.308	239911	12887	20.672
2	7.501	38942	2571	3.355
3	8.056	43497	2576	3.748
4	8.671	5505	291	0.474
5	9.273	1098	107	0.095
6	9.572	5958	443	0.513
7	10.037	1147	86	0.099
8	10.433	173	25	0.015
9	10.758	1224	97	0.105
10	10.993	1866	144	0.161
11	11.861	4776	247	0.412
12	12.690	637899	36338	54.966
13	13.285	2059	169	0.177
14	13.981	1713	113	0.148
15	15.273	11408	584	0.983
16	18.016	1934	114	0.167
17	19.210	75079	2424	6.469
18	20.195	2619	108	0.226
19	21.767	80991	3123	6.979
20	29.816	2742	63	0.236
Total		1160541	62509	

42 h

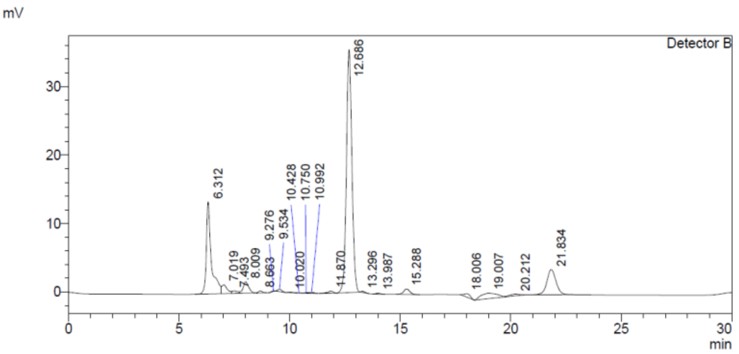

Detector B

Peak#	Ret. Time	Area	Height	Conc.
1	6.312	213014	13464	19.631
2	7.019	22190	1287	2.045
3	7.493	6592	359	0.608
4	8.009	28963	1674	2.669
5	8.663	3779	292	0.348
6	9.276	994	78	0.092
7	9.534	4339	333	0.400
8	10.020	1111	81	0.102
9	10.428	291	34	0.027
10	10.750	946	91	0.087
11	10.992	2671	194	0.246
12	11.870	5557	303	0.512
13	12.686	614157	35436	56.600
14	13.296	1883	161	0.173
15	13.987	1901	128	0.175
16	15.288	15571	803	1.435
17	18.006	9900	483	0.912
18	19.007	39049	771	3.599
19	20.212	8130	253	0.749
20	21.834	104046	3669	9.589
Total		1085083	59892	

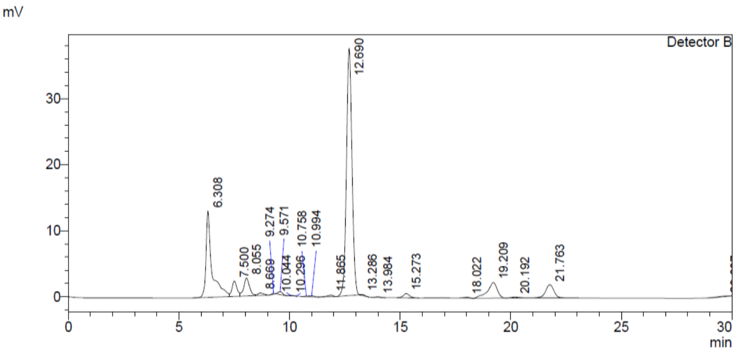

Detector B

Peak#	Ret. Time	Area	Height	Conc.
1	6.308	243734	13055	21.088
2	7.500	36953	2346	3.197
3	8.055	48399	2702	4.187
4	8.669	7360	379	0.637
5	9.274	1167	112	0.101
6	9.571	5806	433	0.502
7	10.044	1189	86	0.103
8	10.296	1	3	0.000
9	10.758	1299	103	0.112
10	10.994	2022	154	0.175
11	11.865	4773	249	0.413
12	12.690	656323	37399	56.785
13	13.286	2174	177	0.188
14	13.984	1698	113	0.147
15	15.273	12010	616	1.039
16	18.022	2131	127	0.184
17	19.209	73205	2368	6.334
18	20.192	2889	119	0.250
19	21.763	50112	1986	4.336
20	29.807	2558	62	0.221
Total		1155804	62587	

48 h

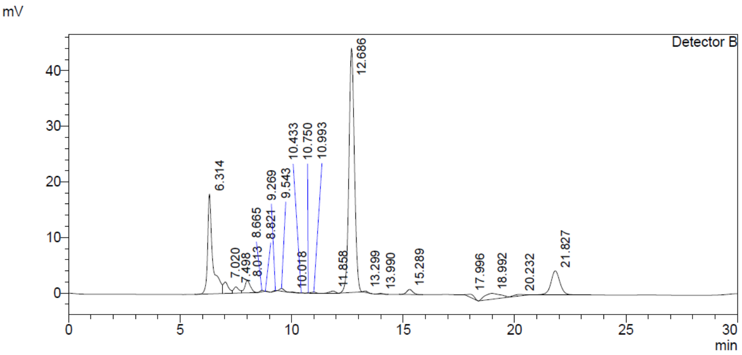

Detector B

Peak#	Ret. Time	Area	Height	Conc.
1	6.314	284519	17938	20.763
2	7.020	33861	2074	2.471
3	7.498	18836	1129	1.375
4	8.013	38101	2192	2.780
5	8.665	2581	264	0.188
6	8.821	385	21	0.028
7	9.269	1367	127	0.100
8	9.543	6713	491	0.490
9	10.018	1108	84	0.081
10	10.433	561	64	0.041
11	10.750	1100	108	0.080
12	10.993	3055	229	0.223
13	11.858	7596	395	0.554
14	12.686	757815	43834	55.302
15	13.299	3112	255	0.227
16	13.990	2241	152	0.164
17	15.289	17558	907	1.281
18	17.996	11574	527	0.845
19	18.992	51074	1057	3.727
20	20.232	9377	278	0.684
21	21.827	117776	4282	8.595
Total		1370310	76406	

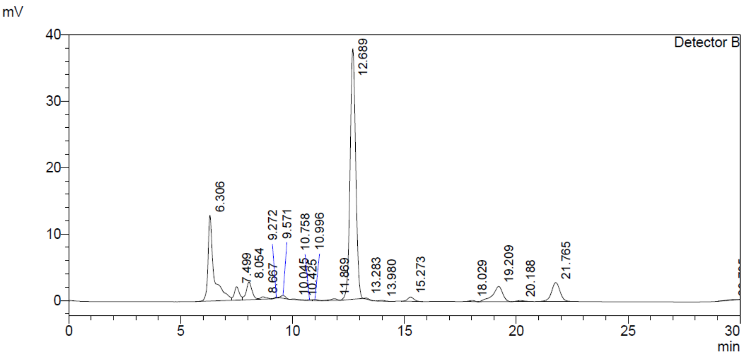

Detector B

Peak#	Ret. Time	Area	Height	Conc.
1	6.306	239677	12907	20.430
2	7.499	32186	2022	2.743
3	8.054	46696	2642	3.980
4	8.667	6951	355	0.592
5	9.272	1315	124	0.112
6	9.571	5742	424	0.489
7	10.045	1497	92	0.128
8	10.425	278	35	0.024
9	10.758	1265	104	0.108
10	10.996	2112	157	0.180
11	11.869	4753	248	0.405
12	12.689	661557	37690	56.390
13	13.283	1945	158	0.166
14	13.980	1634	109	0.139
15	15.273	12471	639	1.063
16	18.029	2589	150	0.221
17	19.209	71373	2309	6.084
18	20.188	3218	131	0.274
19	21.765	73237	2839	6.243
20	29.795	2680	67	0.228
Total		1173175	63203	

C: The curves of fermentation on corncob substrate by NH_3 pretreatment for 1

day followed by H_2O_2 treatment for 7 day at pH6.0 adjusted by $CaCO_3$

0 h

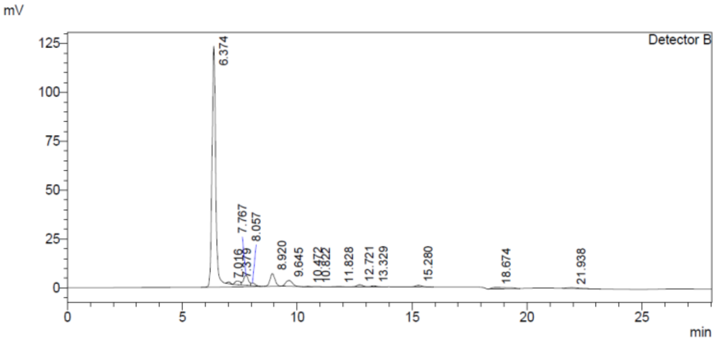

Detector B

Peak#	Ret. Time	Area	Height	Conc.
1	6.374	1448800	123197	79.306
2	7.016	9113	992	0.499
3	7.379	30427	1729	1.666
4	7.767	73652	5146	4.032
5	8.057	16019	1353	0.877
6	8.920	96458	6418	5.280
7	9.645	60314	2892	3.302
8	10.472	2241	175	0.123
9	10.822	1199	71	0.066
10	11.828	3962	160	0.217
11	12.721	17651	1002	0.966
12	13.329	7543	433	0.413
13	15.280	14707	737	0.805
14	18.674	33554	696	1.837
15	21.938	11201	377	0.613
Total		1826841	145379	

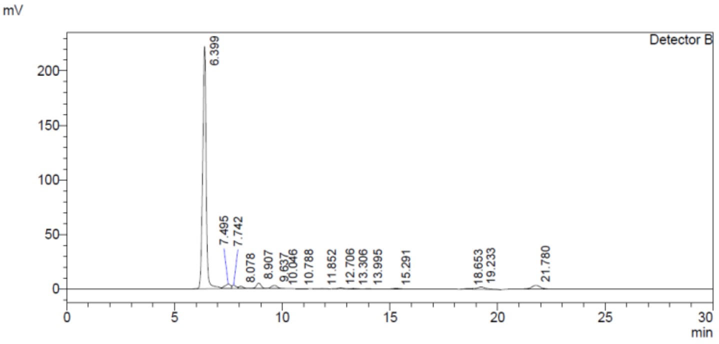

Detector B

Peak#	Ret. Time	Area	Height	Conc.
1	6.399	2514025	222594	83.637
2	7.495	79931	4180	2.659
3	7.742	42179	3101	1.403
4	8.078	29558	2095	0.983
5	8.907	73983	4931	2.461
6	9.637	54870	2905	1.825
7	10.046	16	15	0.001
8	10.788	1164	79	0.039
9	11.852	3940	181	0.131
10	12.706	18795	1112	0.625
11	13.306	6114	362	0.203
12	13.995	1568	101	0.052
13	15.291	10708	538	0.356
14	18.653	11798	525	0.393
15	19.233	62985	2157	2.095
16	21.780	94243	3503	3.135
Total		3005878	248379	

12 h

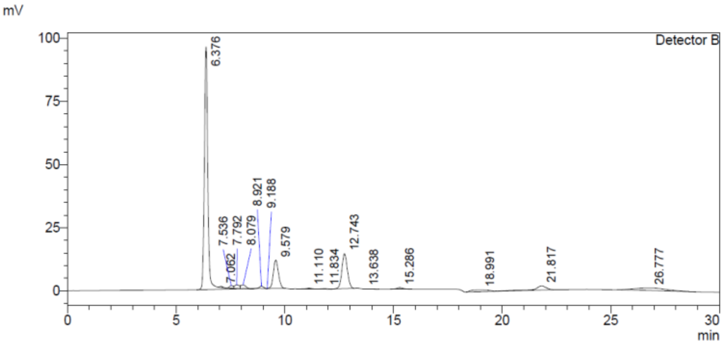

Detector B

Peak#	Ret. Time	Area	Height	Conc.
1	6.376	1061828	96134	59.727
2	7.062	4306	454	0.242
3	7.536	12014	1030	0.676
4	7.792	21480	1454	1.208
5	8.079	23514	1487	1.323
6	8.921	12268	922	0.690
7	9.188	9	5	0.001
8	9.579	178102	11174	10.018
9	11.110	6377	303	0.359
10	11.834	3383	161	0.190
11	12.743	244371	13808	13.746
12	13.638	-522	-3	-0.029
13	15.286	10748	539	0.605
14	18.991	37038	667	2.083
15	21.817	59411	1616	3.342
16	26.777	103485	1032	5.821
Total		1777812	130785	

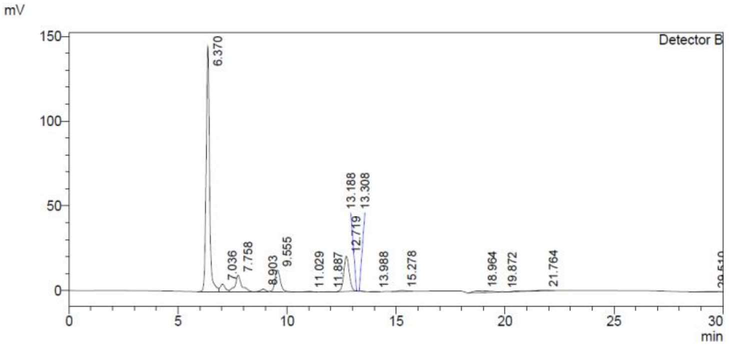

Detector B

Peak#	Ret. Time	Area	Height	Conc.
1	6.370	1567895	144669	61.257
2	7.036	69906	4438	2.731
3	7.758	200242	9679	7.823
4	8.903	24673	1592	0.964
5	9.555	215753	12912	8.429
6	11.029	7854	355	0.307
7	11.887	1955	132	0.076
8	12.719	363436	20750	14.199
9	13.188	1	-2	0.000
10	13.308	2031	181	0.079
11	13.988	1148	79	0.045
12	15.278	10367	519	0.405
13	18.964	50394	875	1.969
14	19.872	3122	174	0.122
15	21.764	33042	496	1.291
16	29.510	7707	189	0.301
Total		2559524	197039	

18 h

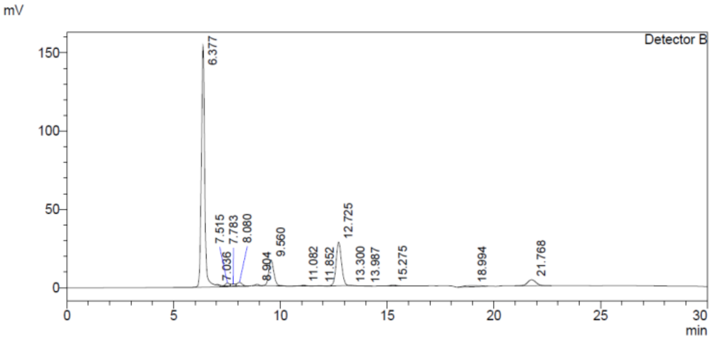

Detector B

Peak#	Ret. Time	Area	Height	Conc.
1	6.377	1711528	153967	62.204
2	7.036	3772	423	0.137
3	7.515	23411	1829	0.851
4	7.783	18120	1354	0.659
5	8.080	39567	2424	1.438
6	8.904	13030	1030	0.474
7	9.560	271101	16495	9.853
8	11.082	12821	663	0.466
9	11.852	3790	191	0.138
10	12.725	494758	27909	17.982
11	13.300	1468	121	0.053
12	13.987	1520	101	0.055
13	15.275	14928	747	0.543
14	18.994	33082	650	1.202
15	21.768	108584	3895	3.946
Total		2751480	211798	

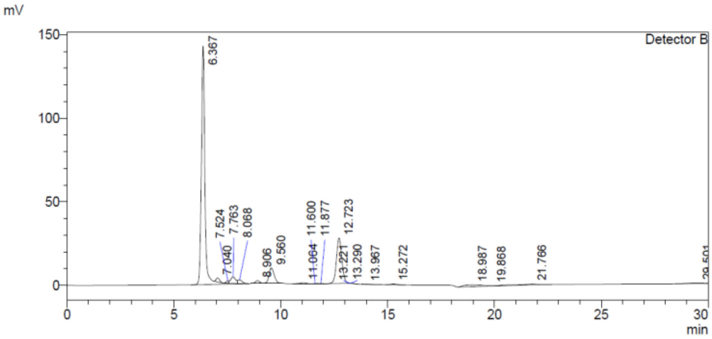

Detector B

Peak#	Ret. Time	Area	Height	Conc.
1	6.367	1629070	142911	63.666
2	7.040	27436	2428	1.072
3	7.524	12728	1408	0.497
4	7.763	60896	3914	2.380
5	8.068	31056	2185	1.214
6	8.906	23169	1654	0.905
7	9.560	151665	9146	5.927
8	11.064	10615	522	0.415
9	11.600	1137	83	0.044
10	11.877	2792	179	0.109
11	12.723	481526	27202	18.818
12	13.221	0	-1	0.000
13	13.290	1783	146	0.070
14	13.967	2234	146	0.087
15	15.272	12301	635	0.481
16	18.987	53327	940	2.084
17	19.868	5759	267	0.225
18	21.766	38627	557	1.510
19	29.501	12669	270	0.495
Total		2558790	194590	

24 h

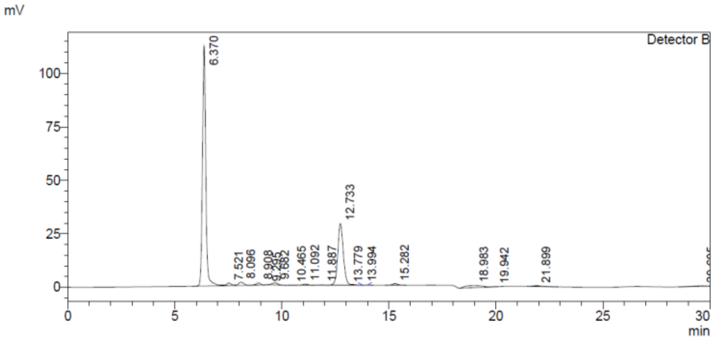

Detector B

Peak#	Ret. Time	Area	Height	Conc.
1	6.370	1220143	112269	63.302
2	7.521	22678	1208	1.177
3	8.096	27663	1597	1.435
4	8.908	14924	980	0.774
5	9.295	199	25	0.010
6	9.682	17586	968	0.912
7	10.465	463	48	0.024
8	11.092	10095	579	0.524
9	11.887	3010	150	0.156
10	12.733	524739	28814	27.224
11	13.779	0	-0	0.000
12	13.994	1053	71	0.055
13	15.282	13713	690	0.711
14	18.983	51334	938	2.663
15	19.942	1059	53	0.055
16	21.899	10138	339	0.526
17	29.605	8688	178	0.451
Total		1927486	148907	

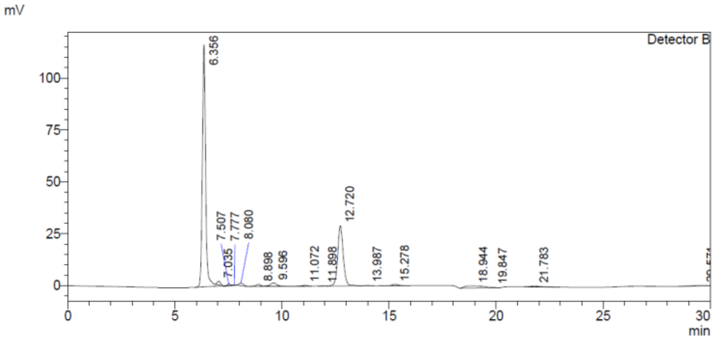

Detector B

Peak#	Ret. Time	Area	Height	Conc.
1	6.356	1243215	116190	62.845
2	7.035	20575	1884	1.040
3	7.507	8788	828	0.444
4	7.777	1440	138	0.073
5	8.080	17673	1284	0.893
6	8.898	16698	995	0.844
7	9.596	38551	1663	1.949
8	11.072	8940	450	0.452
9	11.898	2496	162	0.126
10	12.720	523589	28962	26.468
11	13.987	2786	168	0.141
12	15.278	12300	619	0.622
13	18.944	55252	988	2.793
14	19.847	2636	174	0.133
15	21.783	12486	378	0.631
16	29.571	10785	229	0.545
Total		1978210	155112	

36 h

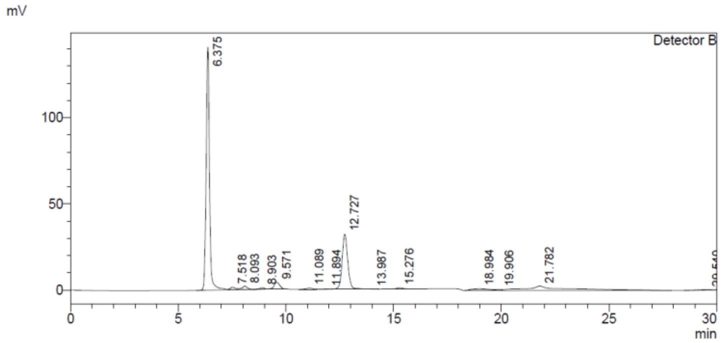

Detector B

Peak#	Ret. Time	Area	Height	Conc.
1	6.375	1542169	141305	56.204
2	7.518	17238	1250	0.628
3	8.093	34251	1848	1.248
4	8.903	13488	882	0.492
5	9.571	70463	3940	2.568
6	11.089	13715	713	0.500
7	11.894	4441	186	0.162
8	12.727	584367	31860	21.297
9	13.987	1384	92	0.050
10	15.276	13993	702	0.510
11	18.984	60643	1011	2.210
12	19.906	12859	608	0.469
13	21.782	364135	2512	13.271
14	29.540	10718	229	0.391
Total		2743863	187139	

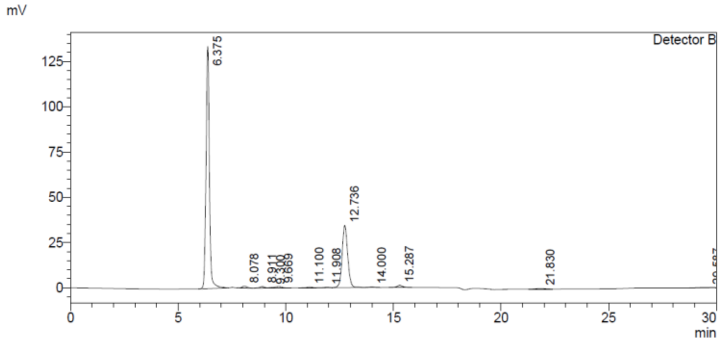

Detector B

Peak#	Ret. Time	Area	Height	Conc.
1	6.375	1445362	133959	66.356
2	8.078	16656	1114	0.765
3	8.911	13962	862	0.641
4	9.300	1363	150	0.063
5	9.669	15923	784	0.731
6	11.100	8936	438	0.410
7	11.908	5810	273	0.267
8	12.736	623848	34417	28.641
9	14.000	5263	328	0.242
10	15.287	24717	1236	1.135
11	21.830	9364	334	0.430
12	29.587	6990	148	0.321
Total		2178192	174044	

42 h

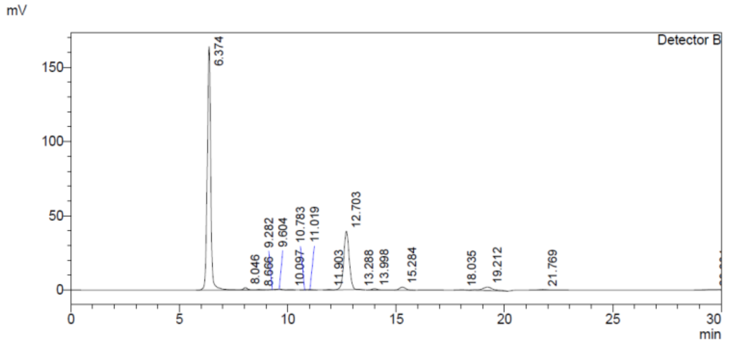

Detector B

Peak#	Ret. Time	Area	Height	Conc.
1	6.374	1839578	164172	67.340
2	8.046	17414	1445	0.637
3	8.666	3197	253	0.117
4	9.282	2145	169	0.079
5	9.604	5015	391	0.184
6	10.097	1968	110	0.072
7	10.783	1147	108	0.042
8	11.019	3781	252	0.138
9	11.903	2984	222	0.109
10	12.703	692628	39369	25.354
11	13.288	1552	140	0.057
12	13.998	14379	870	0.526
13	15.284	40837	2068	1.495
14	18.035	3578	196	0.131
15	19.212	87633	2502	3.208
16	21.769	8755	346	0.320
17	29.684	5195	120	0.190
Total		2731787	212733	

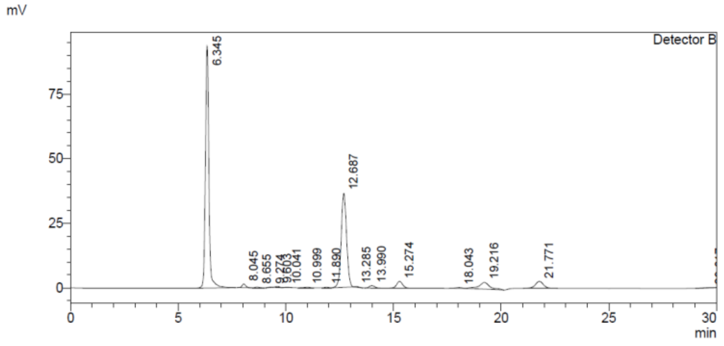

Detector B

Peak#	Ret. Time	Area	Height	Conc.
1	6.345	1054424	93695	53.644
2	8.045	16897	1405	0.860
3	8.655	3423	265	0.174
4	9.274	1525	136	0.078
5	9.603	4202	337	0.214
6	10.041	2024	147	0.103
7	10.999	4450	238	0.226
8	11.890	2472	189	0.126
9	12.687	637545	36433	32.435
10	13.285	2624	214	0.134
11	13.990	15977	959	0.813
12	15.274	51582	2620	2.624
13	18.043	5535	295	0.282
14	19.216	91540	2632	4.657
15	21.771	68802	2662	3.500
16	29.817	2576	59	0.131
Total		1965599	142285	

48 h

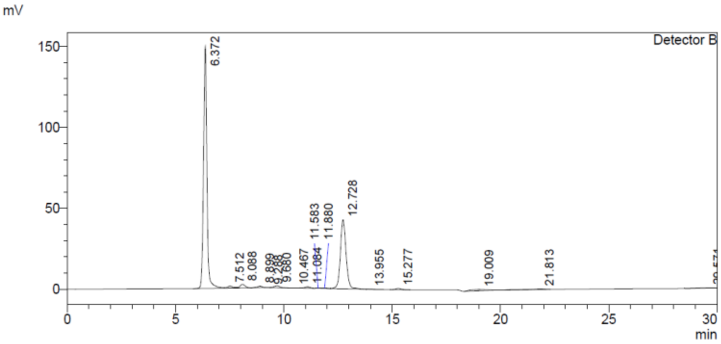

Detector B

Peak#	Ret. Time	Area	Height	Conc.
1	6.372	1624882	149624	61.886
2	7.512	23051	1322	0.878
3	8.088	38092	2278	1.451
4	8.899	15752	970	0.600
5	9.288	1014	114	0.039
6	9.680	23641	1235	0.900
7	10.467	283	30	0.011
8	11.084	17804	919	0.678
9	11.583	2401	167	0.091
10	11.880	6739	341	0.257
11	12.728	785402	42737	29.913
12	13.955	1229	84	0.047
13	15.277	16118	830	0.614
14	19.009	34640	702	1.319
15	21.813	25598	359	0.975
16	29.571	8963	187	0.341
Total		2625610	201900	

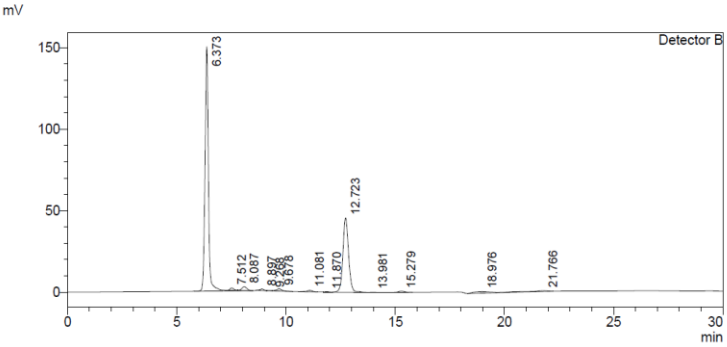

Detector B

Peak#	Ret. Time	Area	Height	Conc.
1	6.373	1626818	150068	61.018
2	7.512	19995	1467	0.750
3	8.087	38859	2401	1.458
4	8.897	11619	855	0.436
5	9.268	1685	162	0.063
6	9.678	23905	1284	0.897
7	11.081	16586	940	0.622
8	11.870	3137	210	0.118
9	12.723	823996	45648	30.906
10	13.981	1572	110	0.059
11	15.279	14924	755	0.560
12	18.976	46637	892	1.749
13	21.766	36404	462	1.365
Total		2666137	205254	

Figure S3 Fed-batch LA fermentation on the corncob by NH_3 pretreatment

A: The curves of fermentation on 8% pretreated and washed corncob substrate fed to 16% corncob from 18 to 24 hat pH6.0 adjusted by $CaCO_3$

0 h

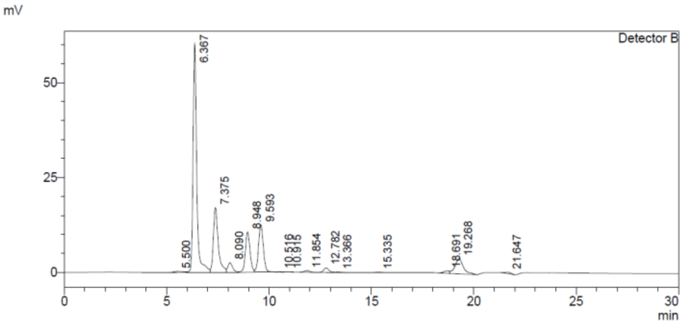

Detector B

Peak#	Ret. Time	Area	Height	Conc.
1	5.480	5460	251	0.291
2	5.885	3124	239	0.167
3	6.371	833665	68091	44.453
4	7.374	274020	18686	14.611
5	7.787	121694	6537	6.489
6	8.947	146074	9642	7.789
7	9.592	139638	9150	7.446
8	11.857	177718	1567	9.476
9	12.777	22517	1187	1.201
10	13.363	1782	105	0.095
11	15.304	1779	98	0.095
12	18.694	14779	708	0.788
13	19.279	133144	4355	7.100
Total		1875393	120617	

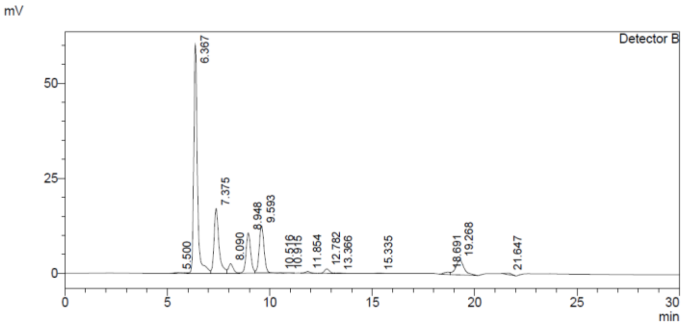

Detector B

Peak#	Ret. Time	Area	Height	Conc.
1	5.500	8508	276	0.000
2	6.367	746978	60097	0.000
3	7.375	283653	17065	0.000
4	8.090	41487	2516	0.000
5	8.948	169081	10599	0.000
6	9.593	204236	12499	0.000
7	10.516	1408	115	0.000
8	10.915	1312	95	0.000
9	11.854	8272	415	0.000
10	12.782	21268	1177	0.000
11	13.366	2398	131	0.000
12	15.335	2135	111	0.000
13	18.691	12685	615	0.000
14	19.268	112019	4012	0.000
15	21.647	8046	381	0.000
Total		1623485	110104	

12 h

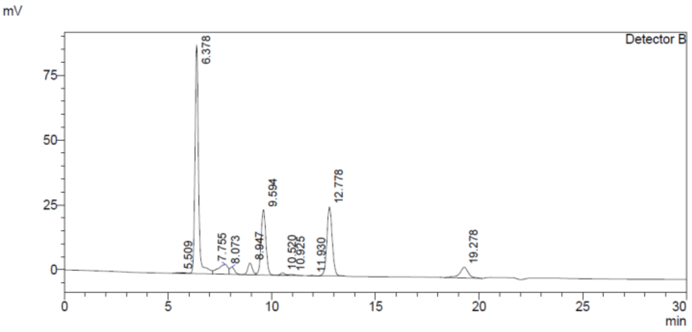

Detector B

Peak#	Ret. Time	Area	Height	Conc.
1	5.509	10467	304	0.453
2	6.378	1040648	87986	45.036
3	7.755	126749	3803	5.485
4	8.073	43706	2824	1.891
5	8.947	69057	4459	2.989
6	9.594	395619	25114	17.121
7	10.520	14064	937	0.609
8	10.925	7831	375	0.339
9	11.930	1466	113	0.063
10	12.778	478018	26534	20.687
11	19.278	123085	4137	5.327
Total		2310710	156586	

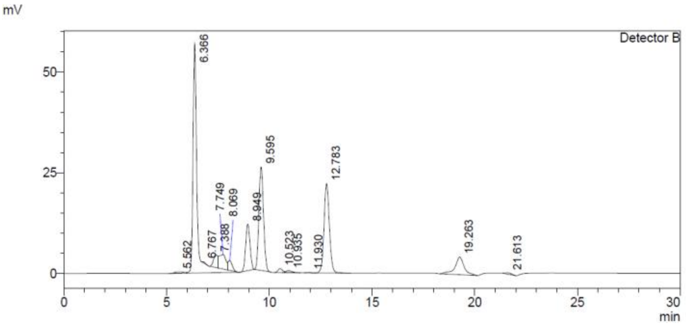

Detector B

Peak#	Ret. Time	Area	Height	Conc.
1	5.562	12514	384	0.587
2	6.366	804242	56833	37.751
3	6.767	2486	185	0.117
4	7.388	45620	3188	2.141
5	7.749	82959	3625	3.894
6	8.069	35251	2466	1.655
7	8.949	171151	11570	8.034
8	9.595	396233	25696	18.599
9	10.523	15104	996	0.709
10	10.935	9734	474	0.457
11	11.930	1773	126	0.083
12	12.783	409247	22210	19.210
13	19.263	137491	4366	6.454
14	21.613	6602	307	0.310
Total		2130407	132426	

18 h

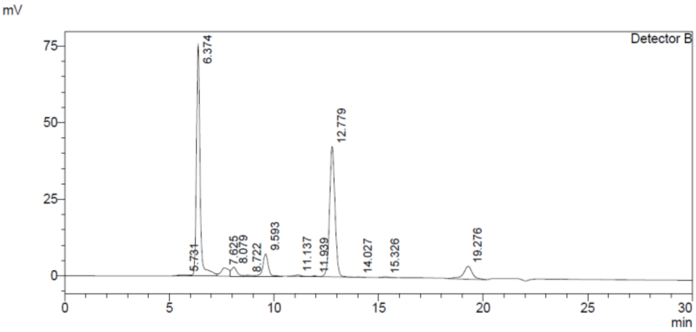

Detector B

Peak#	Ret. Time	Area	Height	Conc.
1	5.731	14160	393	0.000
2	6.374	912410	75437	0.000
3	7.625	71252	2653	0.000
4	8.079	54208	2896	0.000
5	8.722	8134	343	0.000
6	9.593	124067	7268	0.000
7	11.137	10321	507	0.000
8	11.939	2913	211	0.000
9	12.779	771277	42438	0.000
10	14.027	1144	78	0.000
11	15.326	4325	228	0.000
12	19.276	125602	4229	0.000
Total		2099812	136682	

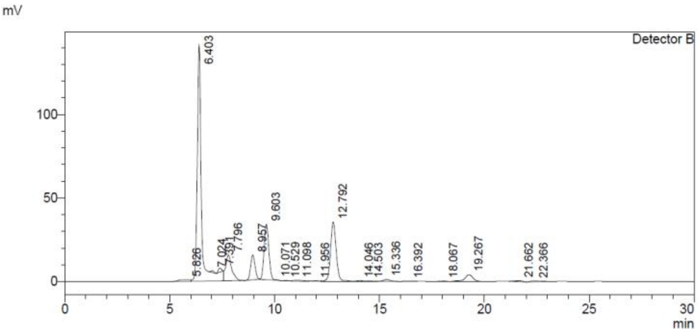

Detector B

Peak#	Ret. Time	Area	Height	Conc.
1	5.826	32328	919	0.826
2	6.403	1947950	141275	49.751
3	7.024	11452	1157	0.292
4	7.391	25127	2417	0.642
5	7.796	317149	15529	8.100
6	8.957	220601	14860	5.634
7	9.603	512317	33218	13.085
8	10.071	1743	172	0.045
9	10.529	2962	255	0.076
10	11.098	15672	507	0.400
11	11.956	2446	169	0.062
12	12.792	656971	35459	16.779
13	14.046	5381	307	0.137
14	14.503	2979	160	0.076
15	15.336	22906	1151	0.585
16	16.392	1462	67	0.037
17	18.067	3089	171	0.079
18	19.267	109304	4097	2.792
19	21.662	11355	479	0.290
20	22.366	12235	410	0.312
Total		3915430	252778	

24 h

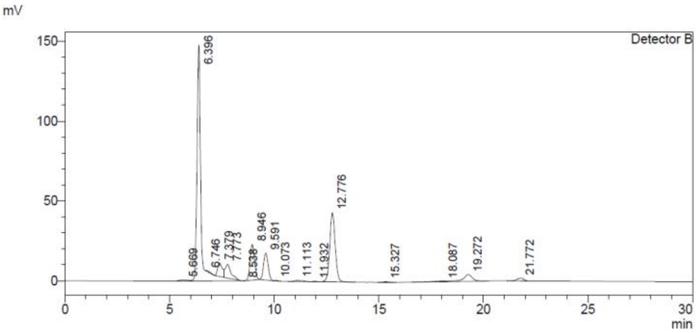

Detector B

Peak#	Ret. Time	Area	Height	Conc.
1	5.669	25126	737	0.647
2	6.396	1956606	147589	50.400
3	6.746	7233	293	0.186
4	7.379	134781	8331	3.472
5	7.773	164634	8502	4.241
6	8.538	1	-2	0.000
7	8.946	330324	22238	8.509
8	9.591	252787	16895	6.511
9	10.073	3390	317	0.087
10	11.113	20317	802	0.523
11	11.932	3203	232	0.083
12	12.776	786212	43260	20.252
13	15.327	4775	249	0.123
14	18.087	16397	340	0.422
15	19.272	132261	4287	3.407
16	21.772	44133	1874	1.137
Total		3882179	255944	

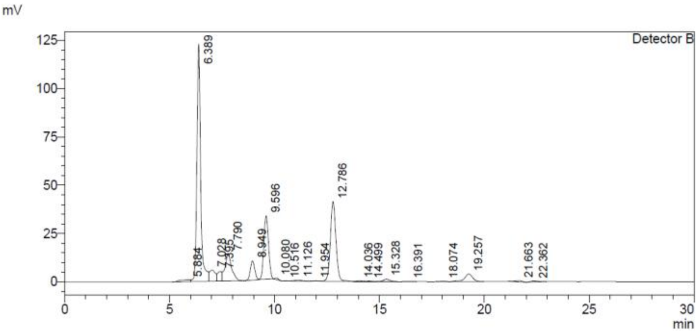

Detector B

Peak#	Ret. Time	Area	Height	Conc.
1	5.884	31436	959	0.862
2	6.389	1512635	122287	41.459
3	7.028	111921	5730	3.068
4	7.395	68352	4916	1.873
5	7.790	316657	14312	8.679
6	8.949	147245	9880	4.036
7	9.596	499952	32740	13.703
8	10.080	9717	852	0.266
9	10.516	858	90	0.024
10	11.126	16125	636	0.442
11	11.954	2431	176	0.067
12	12.786	764179	41333	20.945
13	14.036	4713	298	0.129
14	14.499	3078	167	0.084
15	15.328	25400	1276	0.696
16	16.391	1485	67	0.041
17	18.074	3545	196	0.097
18	19.257	104886	3951	2.875
19	21.663	12624	519	0.346
20	22.362	11228	392	0.308
Total		3648470	240777	

36 h

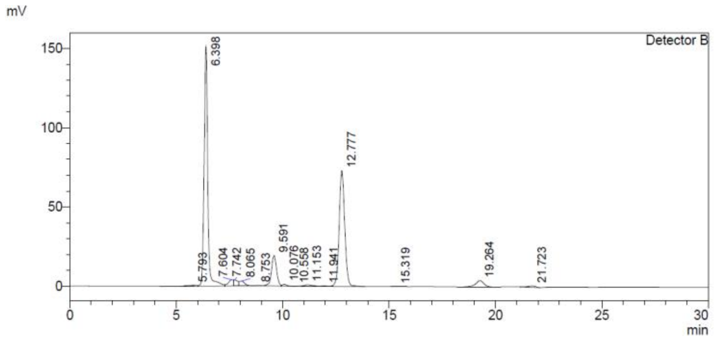

Peak#	Ret. Time	Area	Height	Conc.
1	5.793	25401	747	0.664
2	6.398	1784548	151223	46.675
3	7.604	64552	3918	1.688
4	7.742	52792	3814	1.381
5	8.065	53893	3127	1.410
6	8.753	5447	281	0.142
7	9.591	312418	19218	8.171
8	10.076	15871	1127	0.415
9	10.558	67	11	0.002
10	11.153	29880	1048	0.782
11	11.941	5854	360	0.153
12	12.777	1339193	73165	35.026
13	15.319	1390	75	0.036
14	19.264	113873	4119	2.978
15	21.723	18199	776	0.476
Total		3823378	263009	

Detector B

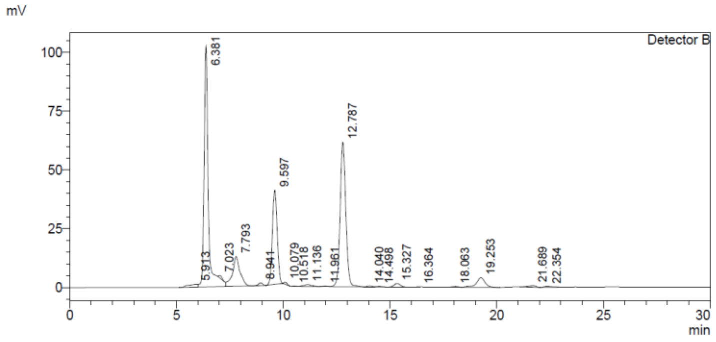

Peak#	Ret. Time	Area	Height	Conc.
1	5.913	35937	1147	0.959
2	6.381	1386479	102405	36.987
3	7.023	11760	1052	0.314
4	7.793	316805	12653	8.451
5	8.941	17331	1200	0.462
6	9.597	620890	40112	16.563
7	10.079	9257	871	0.247
8	10.518	917	98	0.024
9	11.136	23494	884	0.627
10	11.961	3540	237	0.094
11	12.787	1140299	61494	30.420
12	14.040	5094	335	0.136
13	14.498	2102	138	0.056
14	15.327	30955	1549	0.826
15	16.364	1003	75	0.027
16	18.063	4058	221	0.108
17	19.253	112472	4098	3.000
18	21.689	16189	672	0.432
19	22.354	9973	353	0.266
Total		3748555	229596	

42 h

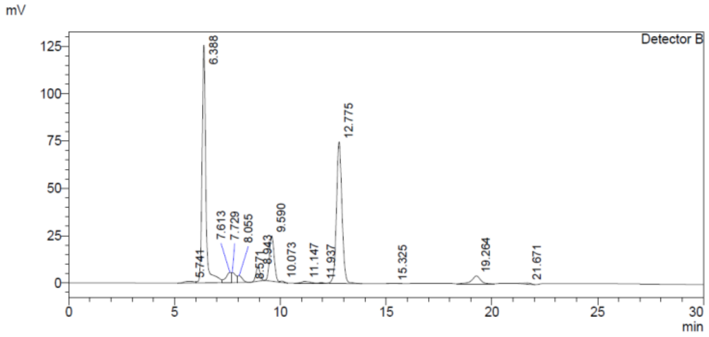

Peak#	Ret. Time	Area	Height	Conc.
1	5.741	29729	876	0.779
2	6.388	1551004	125467	40.663
3	7.613	92298	5314	2.420
4	7.729	74047	5248	1.941
5	8.055	57791	3603	1.515
6	8.571	2	-4	0.000
7	8.943	104325	7369	2.735
8	9.590	363393	23940	9.527
9	10.073	9178	752	0.241
10	11.147	29084	1029	0.762
11	11.937	4655	330	0.122
12	12.775	1364400	74789	35.771
13	15.325	2059	108	0.054
14	19.264	120240	4196	3.152
15	21.671	12074	513	0.317
Total		3814278	253532	

Detector B

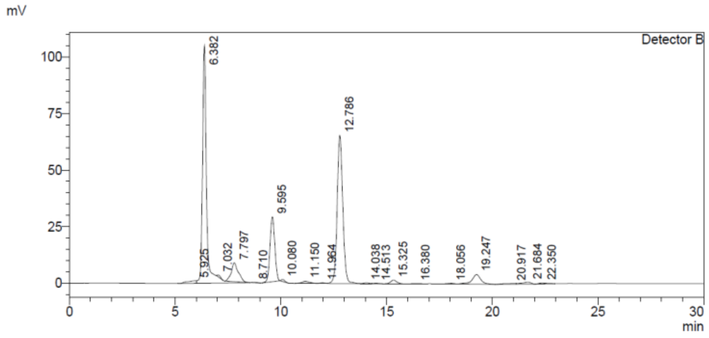

Peak#	Ret. Time	Area	Height	Conc.
1	5.925	34477	1141	0.967
2	6.382	1408376	105002	39.521
3	7.032	9281	796	0.260
4	7.797	196135	8381	5.504
5	8.710	3124	179	0.088
6	9.595	444949	28615	12.486
7	10.080	10466	924	0.294
8	11.150	24302	1003	0.682
9	11.964	4334	280	0.122
10	12.786	1215076	65459	34.097
11	14.038	5038	320	0.141
12	14.513	2961	168	0.083
13	15.325	32607	1625	0.915
14	16.380	1766	73	0.050
15	18.056	3733	205	0.105
16	19.247	120516	4258	3.382
17	20.917	13103	302	0.368
18	21.684	24160	805	0.678
19	22.350	9216	325	0.259
Total		3563618	219862	

48 h

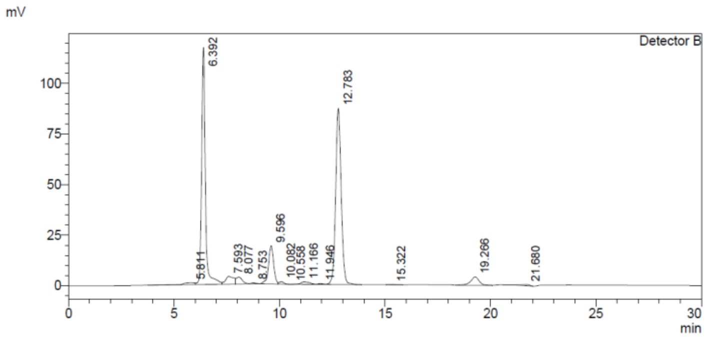

Peak#	Ret. Time	Area	Height	Conc.
1	5.811	32307	917	0.864
2	6.392	1432481	117220	38.310
3	7.593	105120	3814	2.811
4	8.077	66408	3389	1.776
5	8.753	6534	373	0.175
6	9.596	310248	18798	8.297
7	10.082	16909	1183	0.452
8	10.558	68	10	0.002
9	11.166	35839	1272	0.958
10	11.946	7091	429	0.190
11	12.783	1593322	86967	42.611
12	15.322	2297	121	0.061
13	19.266	118069	4158	3.158
14	21.680	12500	524	0.334
Total		3739195	239174	

Detector B

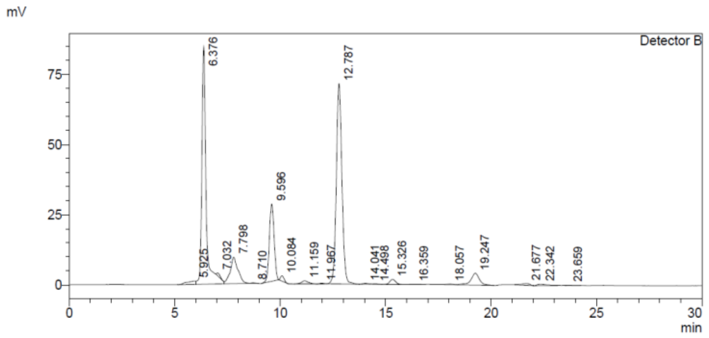

Detector B

Peak#	Ret. Time	Area	Height	Conc.
1	5.925	35533	1185	1.026
2	6.376	1170978	84567	33.816
3	7.032	11387	930	0.329
4	7.798	238663	9404	6.892
5	8.710	2152	181	0.062
6	9.596	425313	27551	12.282
7	10.084	22590	1878	0.652
8	11.159	28204	1162	0.814
9	11.967	3890	278	0.112
10	12.787	1323071	71423	38.208
11	14.041	5178	361	0.150
12	14.498	2318	149	0.067
13	15.326	37215	1861	1.075
14	16.359	1895	82	0.055
15	18.057	3806	210	0.110
16	19.247	117676	4202	3.398
17	21.677	15923	669	0.460
18	22.342	14640	397	0.423
19	23.659	2356	92	0.068
Total		3462789	206583	

60 h

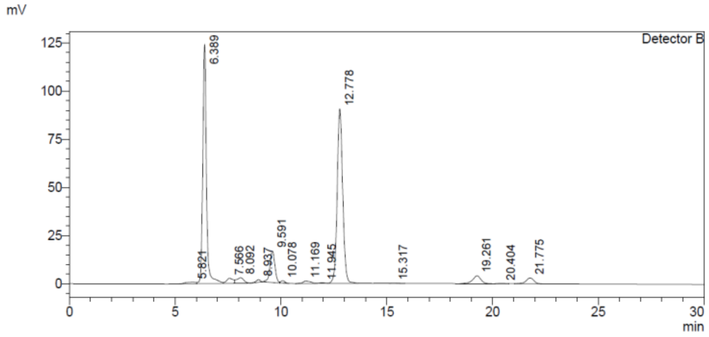

Detector B

Peak#	Ret. Time	Area	Height	Conc.
1	5.821	28378	842	0.740
2	6.389	1485174	123781	38.729
3	7.566	58137	2670	1.516
4	8.092	67282	2933	1.754
5	8.937	18884	1367	0.492
6	9.591	258856	16271	6.750
7	10.078	16846	1250	0.439
8	11.169	35739	1298	0.932
9	11.945	6705	414	0.175
10	12.778	1660303	90468	43.296
11	15.317	3010	152	0.078
12	19.261	117578	4171	3.066
13	20.404	2450	112	0.064
14	21.775	75468	3056	1.968
Total		3834810	248785	

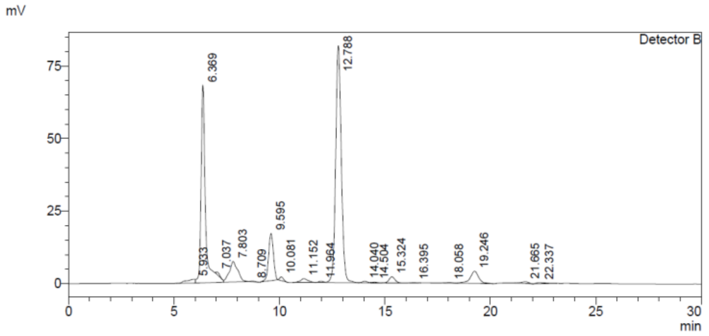

Detector B

Peak#	Ret. Time	Area	Height	Conc.
1	5.933	37157	1230	1.134
2	6.369	992117	68074	30.282
3	7.037	10083	826	0.308
4	7.803	198117	7066	6.047
5	8.709	2971	240	0.091
6	9.595	253835	16235	7.748
7	10.081	15968	1302	0.487
8	11.152	33444	1326	1.021
9	11.964	7429	447	0.227
10	12.788	1519341	81629	46.374
11	14.040	7589	468	0.232
12	14.504	3489	204	0.106
13	15.324	42737	2129	1.304
14	16.395	1539	84	0.047
15	18.058	3978	220	0.121
16	19.246	119503	4221	3.648
17	21.665	14866	623	0.454
18	22.337	12126	398	0.370
Total		3276290	186722	

66 h

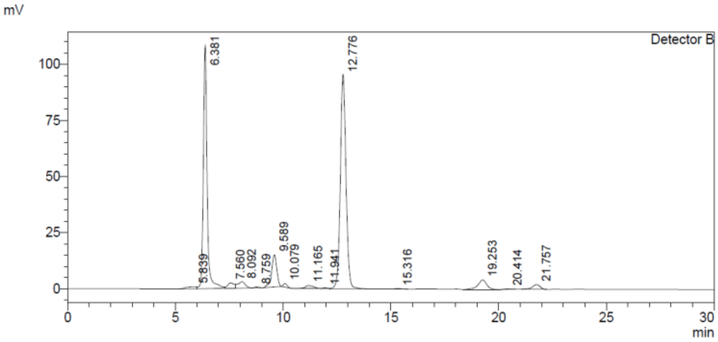

Detector B

Peak#	Ret. Time	Area	Height	Conc.
1	5.839	28675	871	0.775
2	6.381	1320347	108101	35.664
3	7.560	51043	2474	1.379
4	8.092	68711	2900	1.856
5	8.759	6107	373	0.165
6	9.589	230984	14262	6.239
7	10.079	20748	1670	0.560
8	11.165	39950	1438	1.079
9	11.941	7831	466	0.212
10	12.776	1751967	95384	47.322
11	15.316	3283	166	0.089
12	19.253	119828	4264	3.237
13	20.414	2620	120	0.071
14	21.757	50093	2082	1.353
Total		3702188	234571	

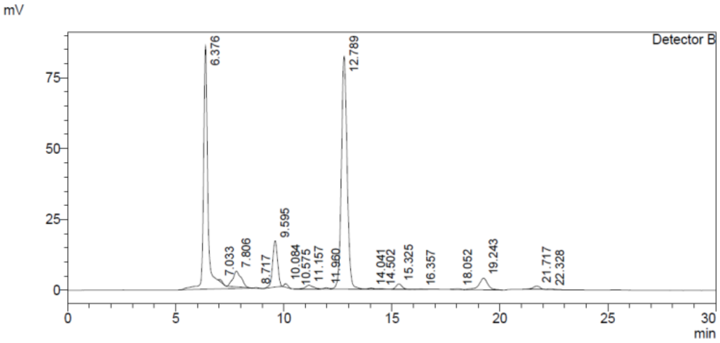

Detector B

Peak#	Ret. Time	Area	Height	Conc.
1	6.376	1244511	85940	36.060
2	7.033	7331	621	0.212
3	7.806	151349	5603	4.385
4	8.717	3799	251	0.110
5	9.595	258050	16389	7.477
6	10.084	15725	1264	0.456
7	10.575	72	10	0.002
8	11.157	33500	1313	0.971
9	11.960	7655	452	0.222
10	12.789	1525318	82199	44.197
11	14.041	6491	419	0.188
12	14.502	3263	183	0.095
13	15.325	38636	1929	1.119
14	16.357	1942	80	0.056
15	18.052	3790	211	0.110
16	19.243	117129	4115	3.394
17	21.717	28481	1240	0.825
18	22.328	4163	187	0.121
Total		3451206	202409	

72 h

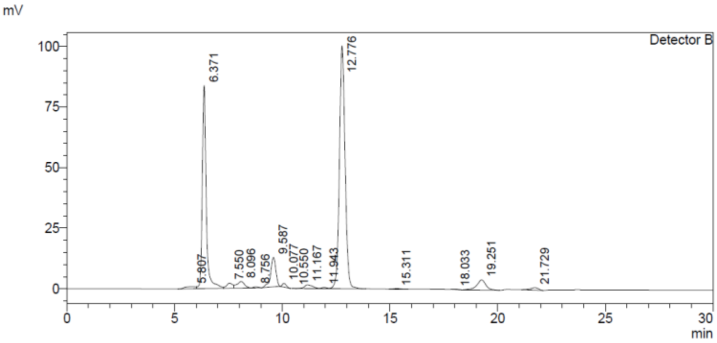

Detector B

Peak#	Ret. Time	Area	Height	Conc.
1	5.807	30224	896	0.871
2	6.371	1064583	83655	30.690
3	7.550	42321	2132	1.220
4	8.096	64052	2721	1.846
5	8.756	6133	390	0.177
6	9.587	201536	12200	5.810
7	10.077	19206	1539	0.554
8	10.550	33	8	0.001
9	11.167	39880	1477	1.150
10	11.943	8677	508	0.250
11	12.776	1840594	99945	53.061
12	15.311	4380	220	0.126
13	18.033	1406	88	0.041
14	19.251	121220	4181	3.495
15	21.729	24595	1045	0.709
Total		3468839	211006	

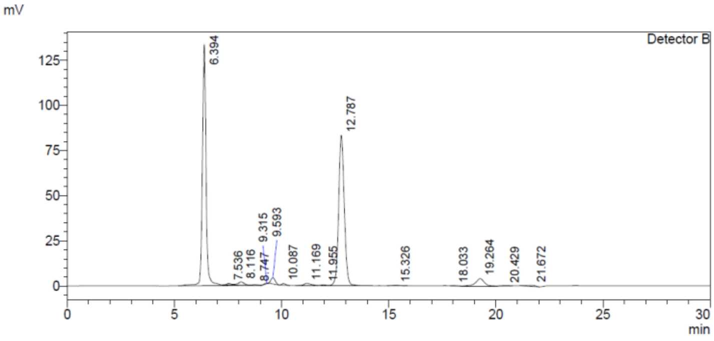

Detector B

Peak#	Ret. Time	Area	Height	Conc.
1	6.394	1604709	133180	46.725
2	7.536	12282	818	0.358
3	8.116	38108	1824	1.110
4	8.747	4921	359	0.143
5	9.315	4490	363	0.131
6	9.593	46994	3416	1.368
7	10.087	10956	849	0.319
8	11.169	30326	1223	0.883
9	11.955	7288	428	0.212
10	12.787	1536056	83424	44.726
11	15.326	6245	307	0.182
12	18.033	1416	85	0.041
13	19.264	114243	4109	3.326
14	20.429	2303	105	0.067
15	21.672	14040	551	0.409
Total		3434377	231040	

84 h

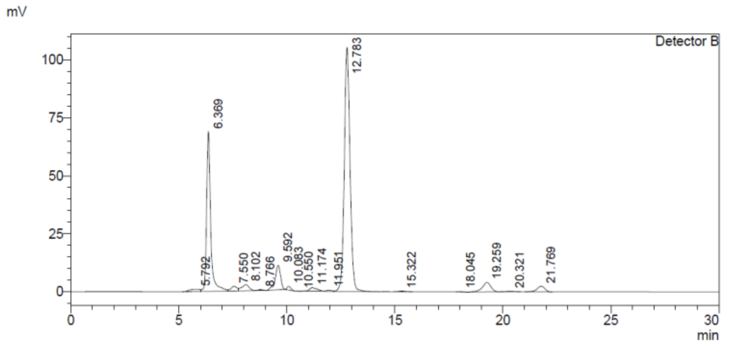

Detector B

Peak#	Ret. Time	Area	Height	Conc.
1	5.792	31270	916	0.914
2	6.369	907211	68797	26.519
3	7.550	40907	2060	1.196
4	8.102	62939	2676	1.840
5	8.766	6099	388	0.178
6	9.592	176933	10531	5.172
7	10.083	19882	1574	0.581
8	10.550	38	8	0.001
9	11.174	40744	1537	1.191
10	11.951	8848	523	0.259
11	12.783	1940222	105174	56.716
12	15.322	5159	259	0.151
13	18.045	1596	96	0.047
14	19.259	114859	4130	3.358
15	20.321	4514	184	0.132
16	21.769	59726	2444	1.746
Total		3420948	201296	

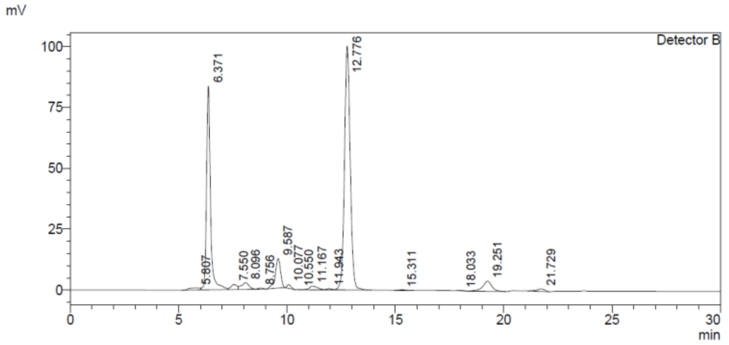

Detector B

Peak#	Ret. Time	Area	Height	Conc.
1	5.807	30224	896	0.871
2	6.371	1064583	83655	30.690
3	7.550	42321	2132	1.220
4	8.096	64052	2721	1.846
5	8.756	6133	390	0.177
6	9.587	201536	12200	5.810
7	10.077	19206	1539	0.554
8	10.550	33	8	0.001
9	11.167	39880	1477	1.150
10	11.943	8677	508	0.250
11	12.776	1840594	99945	53.061
12	15.311	4380	220	0.126
13	18.033	1406	88	0.041
14	19.251	121220	4181	3.495
15	21.729	24595	1045	0.709
Total		3468839	211006	

90 h

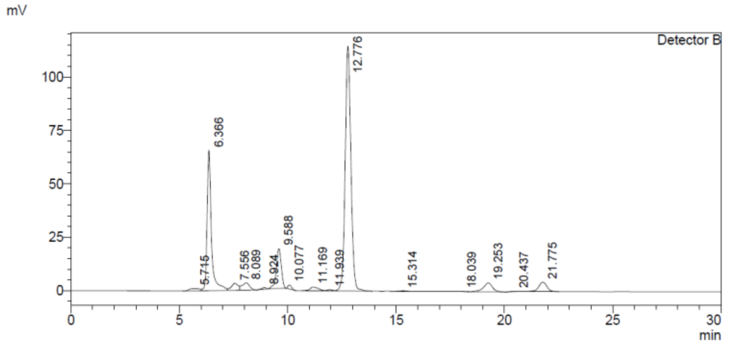

Detector B

Peak#	Ret. Time	Area	Height	Conc.
1	5.715	35947	1059	0.936
2	6.366	908209	65583	23.644
3	7.556	72789	3261	1.895
4	8.089	82017	3459	2.135
5	8.924	12293	813	0.320
6	9.588	293218	18582	7.633
7	10.077	23407	1910	0.609
8	11.169	49572	1746	1.291
9	11.939	10369	600	0.270
10	12.776	2109226	114444	54.911
11	15.314	4032	201	0.105
12	18.039	1707	103	0.044
13	19.253	123145	4229	3.206
14	20.437	3091	140	0.080
15	21.775	112181	4387	2.920
Total		3841202	220516	

Detector B

Peak#	Ret. Time	Area	Height	Conc.
1	6.310	304389	23500	10.955
2	6.638	68041	3072	2.449
3	7.507	60125	4171	2.164
4	8.121	28688	1457	1.033
5	8.732	13114	691	0.472
6	9.264	5923	544	0.213
7	9.528	5351	463	0.193
8	10.025	3070	256	0.111
9	10.492	132	13	0.005
10	11.074	35967	1990	1.294
11	11.905	7907	580	0.285
12	12.727	2049582	113349	73.765
13	13.263	2511	76	0.090
14	13.997	2234	156	0.080
15	15.281	13581	691	0.489
16	16.238	2439	98	0.088
17	17.014	1781	86	0.064
18	18.020	21949	1004	0.790
19	18.984	39571	993	1.424
20	21.756	112162	3084	4.037
Total		2778520	156274	

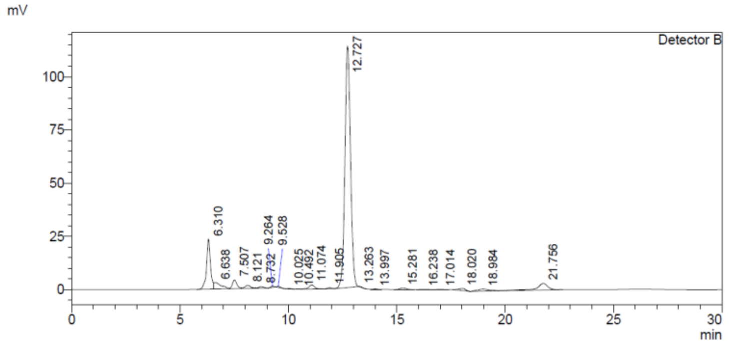

B：The curves of fermentationon 8% pretreated and unwashed corncob substrate fed to 18.4% corncob at 24 h at pH6.0 adjusted by automatic feeding of NaOH solution.

0 h

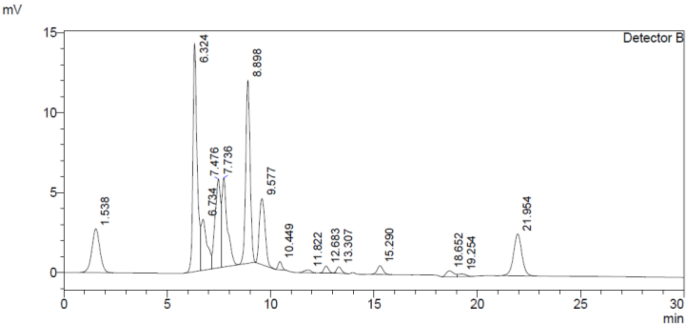

Detector B

Peak#	Ret. Time	Area	Height	Conc.
1	1.538	73798	2746	8.033
2	6.324	203582	14224	22.160
3	6.734	63446	3185	6.906
4	7.476	102762	5548	11.186
5	7.736	103650	5577	11.282
6	8.898	171207	11434	18.636
7	9.577	78802	4123	8.578
8	10.449	6343	501	0.690
9	11.822	4161	182	0.453
10	12.683	6848	432	0.745
11	13.307	6708	405	0.730
12	15.290	10658	543	1.160
13	18.652	9848	373	1.072
14	19.254	4548	181	0.495
15	21.954	72337	2611	7.874
Total		918698	52064	

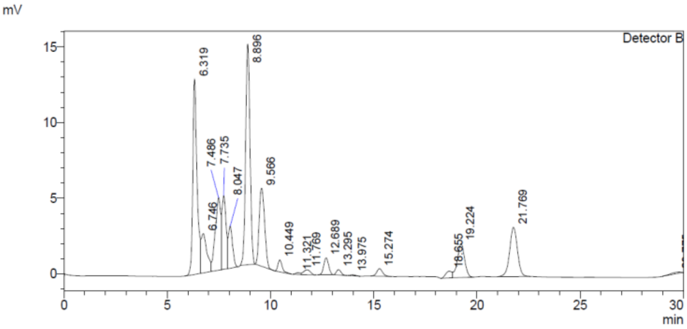

Detector B

Peak#	Ret. Time	Area	Height	Conc.
1	0.218	72589	434	8.012
2	6.327	123506	7570	13.632
3	6.720	62176	3114	6.863
4	7.460	91118	5874	10.057
5	7.705	155397	8538	17.152
6	8.844	146690	9642	16.191
7	9.505	88019	4115	9.715
8	10.431	13485	919	1.488
9	10.867	1063	74	0.117
10	11.211	1086	64	0.120
11	11.960	3720	230	0.411
12	12.702	6357	436	0.702
13	13.253	11078	700	1.223
14	14.036	2498	164	0.276
15	15.309	13699	696	1.512
16	18.894	15511	436	1.712
17	21.633	97991	3699	10.816
Total		905984	46705	

12 h

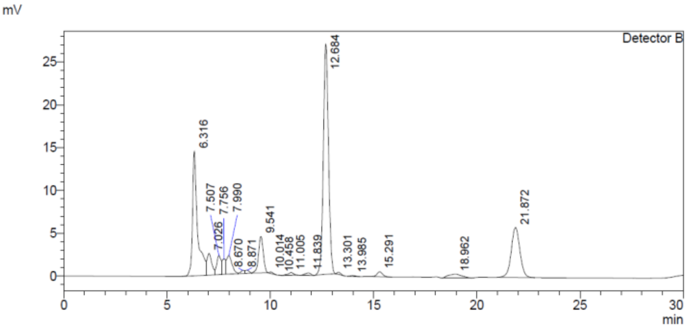

Detector B

Peak#	Ret. Time	Area	Height	Conc.
1	6.316	247710	14522	21.633
2	7.026	42002	2539	3.668
3	7.507	32030	2225	2.797
4	7.756	21690	1817	1.894
5	7.990	39453	2171	3.445
6	8.670	4320	349	0.377
7	8.871	4325	340	0.378
8	9.541	66385	4248	5.797
9	10.014	2029	167	0.177
10	10.458	27	6	0.002
11	11.005	5560	300	0.486
12	11.839	5244	272	0.458
13	12.684	469457	26867	40.998
14	13.301	3091	246	0.270
15	13.985	1733	114	0.151
16	15.291	11448	591	1.000
17	18.962	19914	448	1.739
18	21.872	168657	5827	14.729
Total		1145075	63050	

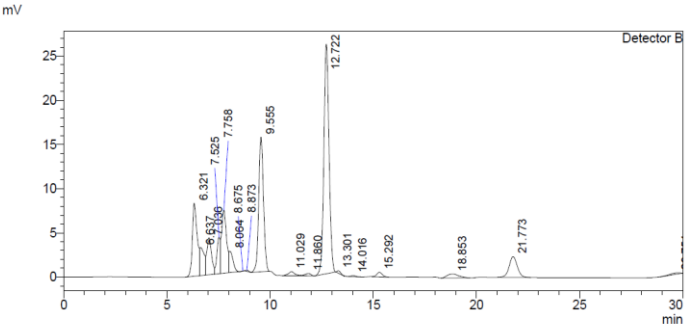

Detector B

Peak#	Ret. Time	Area	Height	Conc.
1	6.321	135210	8246	10.711
2	6.637	46816	3166	3.709
3	7.036	67599	4171	5.355
4	7.525	46842	4115	3.711
5	7.758	120506	7140	9.546
6	8.064	30810	2402	2.441
7	8.675	266	33	0.021
8	8.873	1088	112	0.086
9	9.555	239335	15219	18.960
10	11.029	10145	466	0.804
11	11.860	5590	265	0.443
12	12.722	455617	25944	36.094
13	13.301	3477	279	0.275
14	14.016	2145	142	0.170
15	15.292	10022	524	0.794
16	18.853	17683	447	1.401
17	21.773	63878	2344	5.060
18	29.751	5281	136	0.418
Total		1262311	75152	

18 h

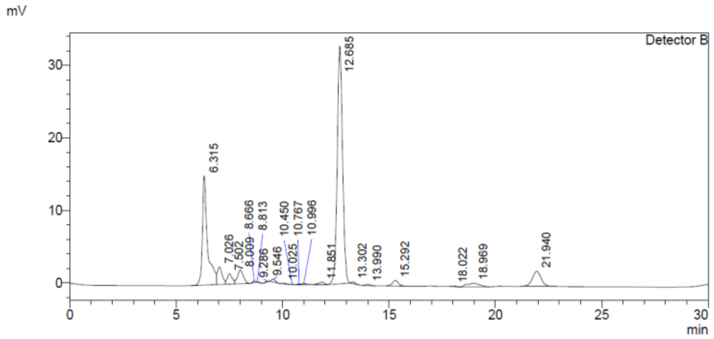

Detector B

Peak#	Ret. Time	Area	Height	Conc.
1	6.315	253326	15049	24.170
2	7.026	39907	2438	3.808
3	7.502	23838	1443	2.274
4	8.009	36181	1908	3.452
5	8.666	2007	205	0.191
6	8.813	382	18	0.036
7	9.286	860	75	0.082
8	9.546	4916	373	0.469
9	10.025	1570	112	0.150
10	10.450	56	12	0.005
11	10.767	935	94	0.089
12	10.996	3057	199	0.292
13	11.851	6499	335	0.620
14	12.685	571702	32693	54.547
15	13.302	2845	232	0.271
16	13.990	2360	155	0.225
17	15.292	14791	760	1.411
18	18.022	1015	65	0.097
19	18.969	21896	522	2.089
20	21.940	59955	2094	5.720
Total		1048097	58779	

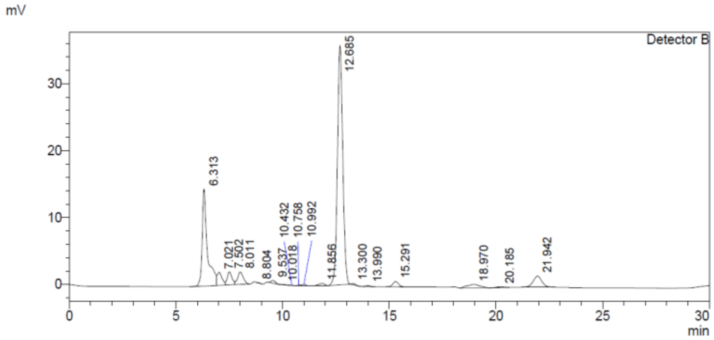

Detector B

Peak#	Ret. Time	Area	Height	Conc.
1	6.313	247292	14520	22.935
2	7.021	31011	1942	2.876
3	7.502	31044	1952	2.879
4	8.011	33994	1867	3.153
5	8.804	314	7	0.029
6	9.537	5789	422	0.537
7	10.018	934	71	0.087
8	10.432	371	43	0.034
9	10.758	765	80	0.071
10	10.992	2489	184	0.231
11	11.856	6817	352	0.632
12	12.685	625444	35706	58.005
13	13.300	2517	208	0.233
14	13.990	2471	162	0.229
15	15.291	15793	812	1.465
16	18.970	21175	517	1.964
17	20.185	2171	86	0.201
18	21.942	47861	1674	4.439
Total		1078252	60604	

24 h

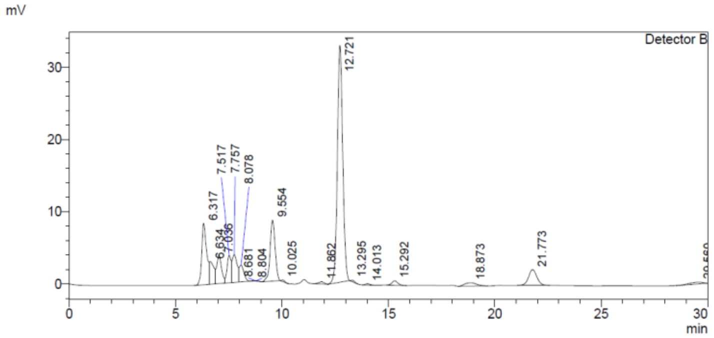

Detector B

Peak#	Ret. Time	Area	Height	Conc.
1	6.317	135427	8510	11.095
2	6.634	47135	3132	3.862
3	7.036	62522	3875	5.122
4	7.517	49056	3787	4.019
5	7.757	63168	3856	5.175
6	8.078	35372	2329	2.898
7	8.681	745	79	0.061
8	8.804	365	26	0.030
9	9.554	135889	8430	11.133
10	10.025	2189	214	0.179
11	11.862	5576	296	0.457
12	12.721	577137	32757	47.283
13	13.295	2456	217	0.201
14	14.013	2460	161	0.202
15	15.292	11464	602	0.939
16	18.873	18452	472	1.512
17	21.773	59643	2188	4.886
18	29.560	11554	258	0.947
Total		1220612	71190	

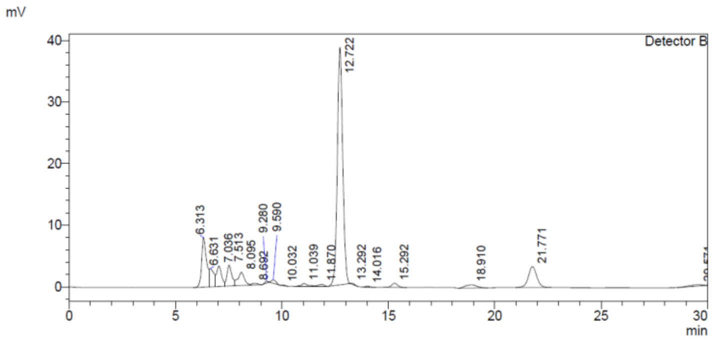

Detector B

Peak#	Ret. Time	Area	Height	Conc.
1	6.313	128479	8042	10.829
2	6.631	45496	2960	3.835
3	7.036	54242	3355	4.572
4	7.513	54879	3366	4.626
5	8.095	45482	2137	3.834
6	8.692	5079	259	0.428
7	9.280	3215	297	0.271
8	9.590	8192	530	0.690
9	10.032	1112	104	0.094
10	11.039	10881	479	0.917
11	11.870	7153	351	0.603
12	12.722	681003	38524	57.402
13	13.292	1686	152	0.142
14	14.016	2468	162	0.208
15	15.292	12951	671	1.092
16	18.910	20394	548	1.719
17	21.771	92518	3382	7.798
18	29.571	11153	252	0.940
Total		1186383	65573	

36 h

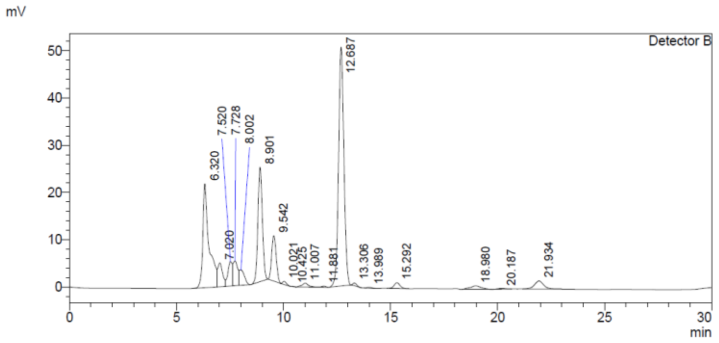

Detector B

Peak#	Ret. Time	Area	Height	Conc.
1	6.320	444939	22015	19.755
2	7.020	82364	5027	3.657
3	7.520	73502	5181	3.263
4	7.728	81970	5251	3.639
5	8.002	52484	3249	2.330
6	8.901	355195	24229	15.770
7	9.542	137270	9597	6.095
8	10.021	7851	638	0.349
9	10.425	248	36	0.011
10	11.007	17635	819	0.783
11	11.881	3232	235	0.144
12	12.687	883096	50547	39.209
13	13.306	7037	566	0.312
14	13.989	3201	215	0.142
15	15.292	24448	1247	1.085
16	18.980	25070	726	1.113
17	20.187	3182	128	0.141
18	21.934	49580	1685	2.201
Total		2252303	131391	

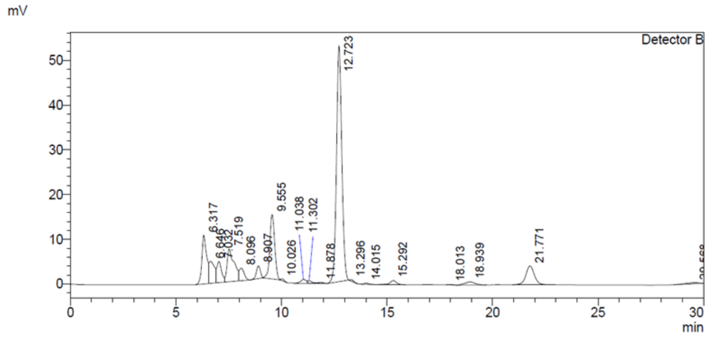

Detector B

Peak#	Ret. Time	Area	Height	Conc.
1	6.317	174129	10905	8.903
2	6.646	85330	4925	4.363
3	7.032	74276	4720	3.798
4	7.519	171243	7337	8.755
5	8.096	45640	2815	2.333
6	8.907	38014	2841	1.944
7	9.555	226258	14373	11.568
8	10.026	3861	381	0.197
9	11.038	16536	906	0.845
10	11.302	9283	596	0.475
11	11.878	5721	307	0.292
12	12.723	928809	52679	47.487
13	13.296	3854	322	0.197
14	14.015	3968	260	0.203
15	15.292	19296	914	0.987
16	18.013	1284	80	0.066
17	18.939	23315	720	1.192
18	21.771	114227	4170	5.840
19	29.568	10859	245	0.555
Total		1955902	109497	

42 h

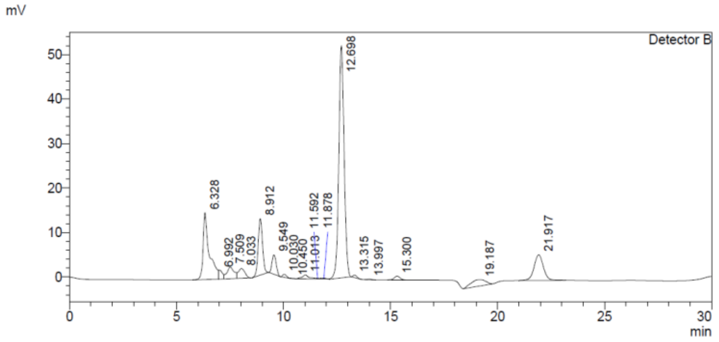

Detector B

Peak#	Ret. Time	Area	Height	Conc.
1	6.328	301297	14980	16.092
2	6.992	23770	2000	1.270
3	7.509	61656	2653	3.293
4	8.033	46193	2157	2.467
5	8.912	181519	12704	9.695
6	9.549	58514	4358	3.125
7	10.030	8657	685	0.462
8	10.450	214	33	0.011
9	11.013	19380	854	1.035
10	11.592	1627	154	0.087
11	11.878	2749	192	0.147
12	12.698	900677	52096	48.105
13	13.315	7351	574	0.393
14	13.997	1997	143	0.107
15	15.300	17441	891	0.932
16	19.187	67076	1345	3.583
17	21.917	172191	5770	9.197
Total		1872309	101588	

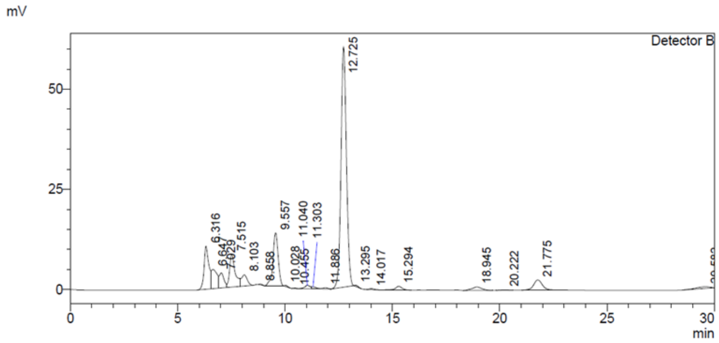

Detector B

Peak#	Ret. Time	Area	Height	Conc.
1	6.316	167921	10769	8.523
2	6.647	89201	4815	4.528
3	7.029	57465	3773	2.917
4	7.515	148760	8255	7.551
5	8.103	51572	2806	2.618
6	8.858	1675	175	0.085
7	9.557	218723	13237	11.102
8	10.028	2900	293	0.147
9	10.455	1120	120	0.057
10	11.040	15253	867	0.774
11	11.303	7864	526	0.399
12	11.886	5281	300	0.268
13	12.725	1060390	59963	53.823
14	13.295	3054	276	0.155
15	14.017	3902	256	0.198
16	15.294	19237	924	0.976
17	18.945	27047	864	1.373
18	20.222	1789	73	0.091
19	21.775	68722	2514	3.488
20	29.582	18277	408	0.928
Total		1970155	111213	

60 h

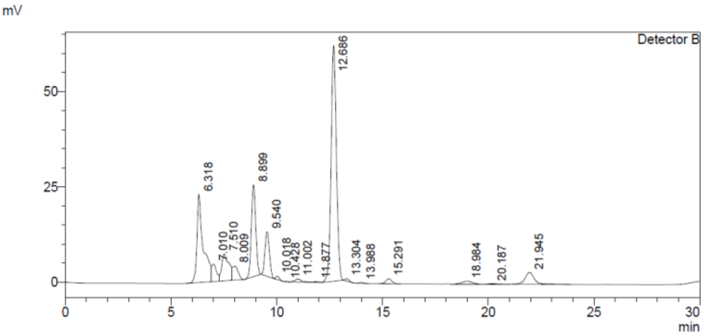

Detector B

Peak#	Ret. Time	Area	Height	Conc.
1	6.318	480183	23050	18.593
2	7.010	70499	4638	2.730
3	7.510	171953	7059	6.658
4	8.009	66286	3686	2.567
5	8.899	350046	24031	13.554
6	9.540	166325	11601	6.440
7	10.018	10538	880	0.408
8	10.428	727	91	0.028
9	11.002	18261	838	0.707
10	11.877	3453	253	0.134
11	12.686	1080006	61765	41.819
12	13.304	8800	682	0.341
13	13.988	3313	225	0.128
14	15.291	26185	1331	1.014
15	18.984	30006	862	1.162
16	20.187	3583	144	0.139
17	21.945	92385	3105	3.577
Total		2582550	144242	

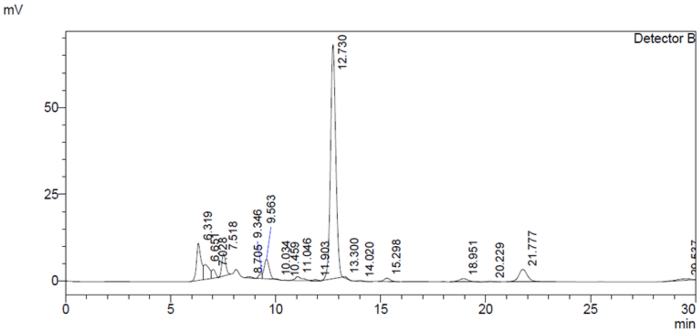

Detector B

Peak#	Ret. Time	Area	Height	Conc.
1	6.319	162059	10721	8.658
2	6.651	77463	4284	4.138
3	7.028	32441	2528	1.733
4	7.518	84705	6500	4.525
5	8.705	5450	304	0.291
6	9.346	13868	1355	0.741
7	9.563	93159	5630	4.977
8	10.034	1394	141	0.074
9	10.459	738	83	0.039
10	11.046	28138	1149	1.503
11	11.903	4578	293	0.245
12	12.730	1191783	67439	63.669
13	13.300	4346	371	0.232
14	14.020	3730	250	0.199
15	15.298	18618	959	0.995
16	18.951	29206	953	1.560
17	20.229	2935	111	0.157
18	21.777	96600	3523	5.161
19	29.537	20627	448	1.102
Total		1871840	107040	

66 h

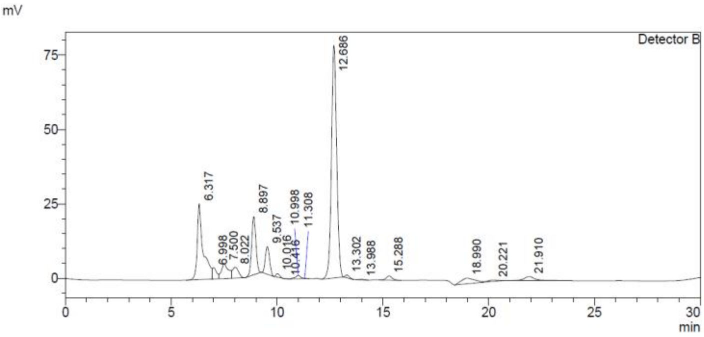

Detector B

Peak#	Ret. Time	Area	Height	Conc.
1	6.317	494833	25474	18.210
2	6.998	54682	3797	2.012
3	7.500	116452	5006	4.285
4	8.022	74830	3693	2.754
5	8.897	275197	19550	10.127
6	9.537	127451	9276	4.690
7	10.016	14809	1195	0.545
8	10.416	331	45	0.012
9	10.998	19234	1099	0.708
10	11.308	1352	154	0.050
11	12.686	1351787	78108	49.745
12	13.302	11502	898	0.423
13	13.988	3306	231	0.122
14	15.288	27943	1432	1.028
15	18.990	82449	1910	3.034
16	20.221	15562	433	0.573
17	21.910	45705	1312	1.682
Total		2717425	153613	

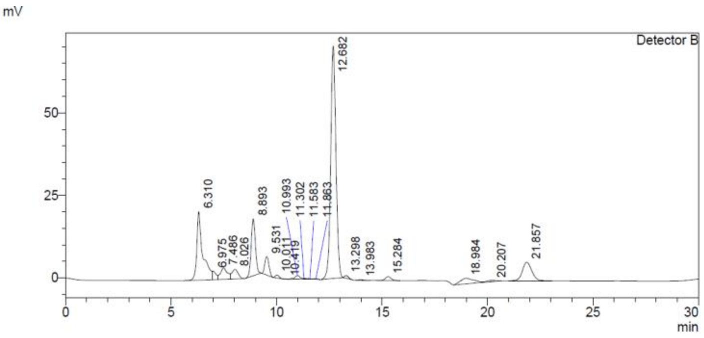

Detector B

Peak#	Ret. Time	Area	Height	Conc.
1	6.310	403961	20740	16.570
2	6.975	31038	2586	1.273
3	7.486	80633	3470	3.307
4	8.026	59947	2851	2.459
5	8.893	243525	17204	9.989
6	9.531	74529	5600	3.057
7	10.011	11452	911	0.470
8	10.419	338	48	0.014
9	10.993	19270	1094	0.790
10	11.302	2225	198	0.091
11	11.583	566	63	0.023
12	11.863	1833	160	0.075
13	12.682	1213883	70288	49.793
14	13.298	10278	802	0.422
15	13.983	2722	191	0.112
16	15.284	23284	1192	0.955
17	18.984	72529	1684	2.975
18	20.207	12144	358	0.498
19	21.857	173724	5622	7.126
Total		2437880	135062	

72 h

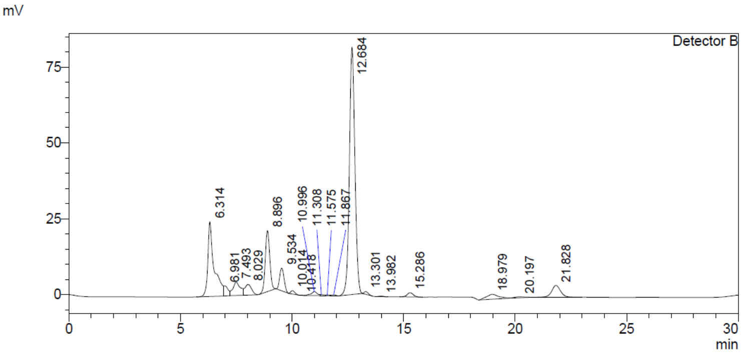

Detector B

Peak#	Ret. Time	Area	Height	Conc.
1	6.314	485637	24615	17.488
2	6.981	43313	3315	1.560
3	7.493	103695	4413	3.734
4	8.029	72573	3493	2.613
5	8.896	286416	20109	10.314
6	9.534	101030	7462	3.638
7	10.014	14001	1115	0.504
8	10.418	301	43	0.011
9	10.996	24777	1329	0.892
10	11.308	4703	353	0.169
11	11.575	2425	229	0.087
12	11.867	4244	298	0.153
13	12.684	1413187	81388	50.888
14	13.301	11728	918	0.422
15	13.982	3172	223	0.114
16	15.286	27454	1401	0.989
17	18.979	61221	1584	2.205
18	20.197	10751	335	0.387
19	21.828	106408	3866	3.832
Total		2777035	156492	

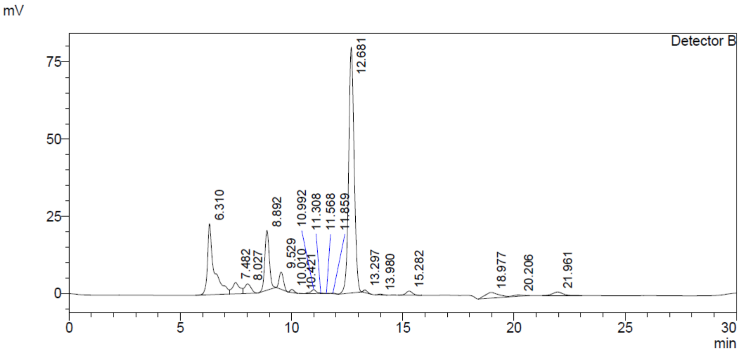

Detector B

Peak#	Ret. Time	Area	Height	Conc.
1	6.310	477309	22972	18.708
2	7.482	85567	3671	3.354
3	8.027	64289	3086	2.520
4	8.892	276695	19387	10.845
5	9.529	73743	5587	2.890
6	10.010	12322	978	0.483
7	10.421	428	60	0.017
8	10.992	22629	1269	0.887
9	11.308	1253	164	0.049
10	11.568	735	73	0.029
11	11.859	1656	154	0.065
12	12.681	1373085	79416	53.817
13	13.297	10731	848	0.421
14	13.980	3020	213	0.118
15	15.282	26212	1339	1.027
16	18.977	74842	1810	2.933
17	20.206	13490	394	0.529
18	21.961	33389	1096	1.309
Total		2551393	142516	

C: the curves of fermentation on 4% pretreated and washed corncob substrate fed to 8% with pretreated and unwashed corncob at 12 h, and fed to 16% with pretreated and unwashed corncob at 24 h.

0 h

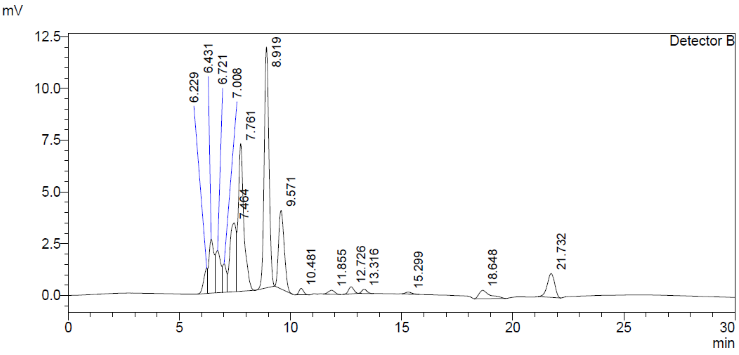

Detector B

Peak#	Ret. Time	Area	Height	Conc.
1	6.229	14071	1211	2.373
2	6.431	41135	2611	6.938
3	6.721	33357	2041	5.626
4	7.008	17399	1376	2.934
5	7.464	64454	3329	10.870
6	7.761	124665	7126	21.025
7	8.919	173501	11618	29.262
8	9.571	65557	3793	11.056
9	10.481	4457	312	0.752
10	11.855	3807	187	0.642
11	12.726	5582	341	0.941
12	13.316	3279	203	0.553
13	15.299	1721	91	0.290
14	18.648	13548	397	2.285
15	21.732	26397	1152	4.452
Total		592928	35788	

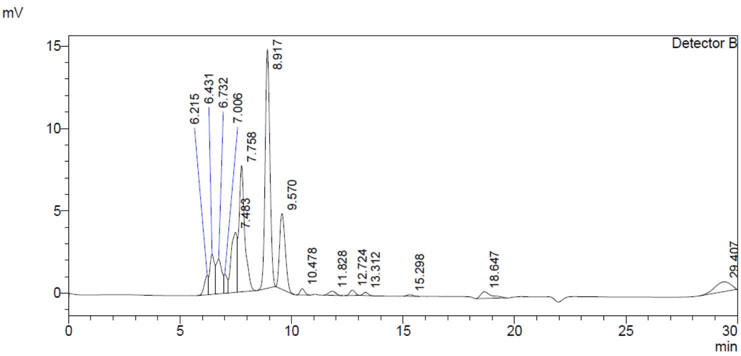

Detector B

Peak#	Ret. Time	Area	Height	Conc.
1	6.215	14601	1184	2.193
2	6.431	36774	2429	5.523
3	6.732	36238	2108	5.442
4	7.006	12829	1150	1.927
5	7.483	68732	3644	10.322
6	7.758	135680	7659	20.377
7	8.917	216372	14472	32.496
8	9.570	79965	4598	12.009
9	10.478	5550	385	0.834
10	11.828	5424	253	0.815
11	12.724	5390	330	0.810
12	13.312	3096	195	0.465
13	15.298	1763	97	0.265
14	18.647	13672	402	2.053
15	29.407	29761	586	4.470
Total		665848	39493	

12 h

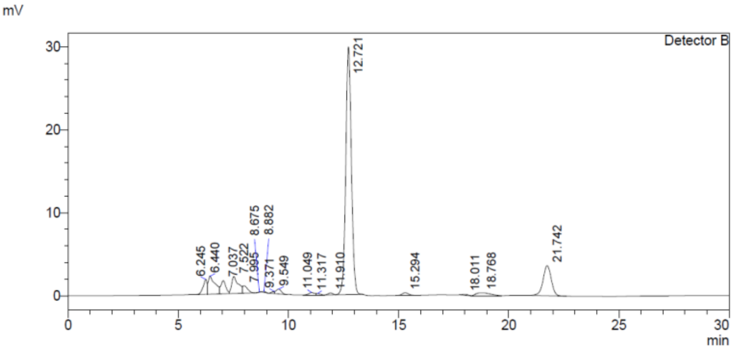

Detector B

Peak#	Ret. Time	Area	Height	Conc.
1	6.245	22561	1816	2.738
2	6.440	46938	2168	5.696
3	7.037	25068	1585	3.042
4	7.522	42796	2038	5.193
5	7.995	14176	881	1.720
6	8.675	295	37	0.036
7	8.882	1065	105	0.129
8	9.371	2168	229	0.263
9	9.549	9416	578	1.143
10	11.049	5464	303	0.663
11	11.317	2049	174	0.249
12	11.910	3481	245	0.422
13	12.721	532245	29846	64.589
14	15.294	6302	326	0.765
15	18.011	1657	102	0.201
16	18.768	15773	354	1.914
17	21.742	92592	3631	11.236
Total		824046	44418	

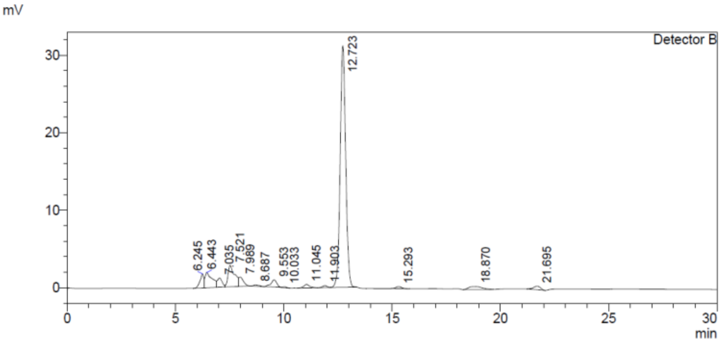

Detector B

Peak#	Ret. Time	Area	Height	Conc.
1	6.245	22187	1807	2.835
2	6.443	43830	1958	5.600
3	7.035	18686	1183	2.387
4	7.521	59296	2773	7.576
5	7.989	17793	1180	2.273
6	8.687	2764	147	0.353
7	9.553	17137	926	2.189
8	10.033	1277	112	0.163
9	11.045	8189	434	1.046
10	11.903	4106	264	0.525
11	12.723	555128	31149	70.922
12	15.293	4988	264	0.637
13	18.870	17361	400	2.218
14	21.695	9983	460	1.275
Total		782726	43055	

18 h

Detector B

Peak#	Ret. Time	Area	Height	Conc.
1	6.295	93268	4486	8.086
2	6.636	43726	2809	3.791
3	7.037	46536	3012	4.035
4	7.520	56561	3009	4.904
5	8.056	23323	1207	2.022
6	8.895	3647	332	0.316
7	9.283	1041	102	0.090
8	9.559	20762	1506	1.800
9	10.031	1065	96	0.092
10	11.053	7072	417	0.613
11	11.317	7118	414	0.617
12	11.888	7014	396	0.608
13	12.720	739312	41960	64.097
14	13.305	5061	400	0.439
15	13.998	1334	90	0.116
16	15.290	10721	558	0.929
17	18.019	1842	109	0.160
18	18.941	20115	577	1.744
19	21.738	63904	2564	5.540
Total		1153423	64044	

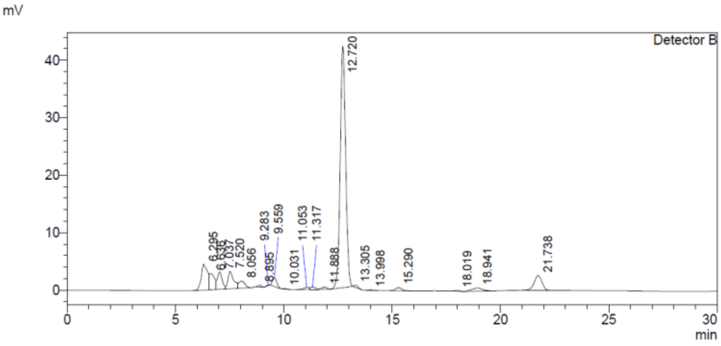

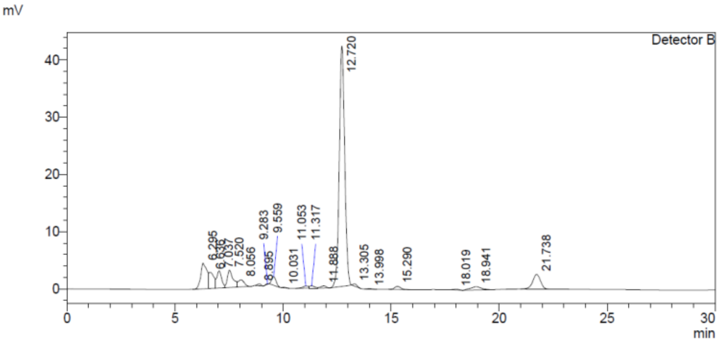

Detector B

Peak#	Ret. Time	Area	Height	Conc.
1	6.297	91914	4627	7.726
2	6.643	47877	2863	4.024
3	7.039	45270	2896	3.805
4	7.521	78645	3901	6.610
5	8.035	25288	1422	2.126
6	8.746	412	29	0.035
7	8.884	2094	198	0.176
8	9.562	64646	3839	5.434
9	10.032	1279	121	0.107
10	10.473	162	20	0.014
11	11.053	16057	616	1.350
12	11.887	6857	371	0.576
13	12.725	760621	42944	63.934
14	13.297	1255	106	0.105
15	14.006	1267	84	0.107
16	15.296	9586	501	0.806
17	18.012	1576	98	0.132
18	18.943	24300	738	2.042
19	21.699	10599	472	0.891
Total		1189705	65847	

24 h

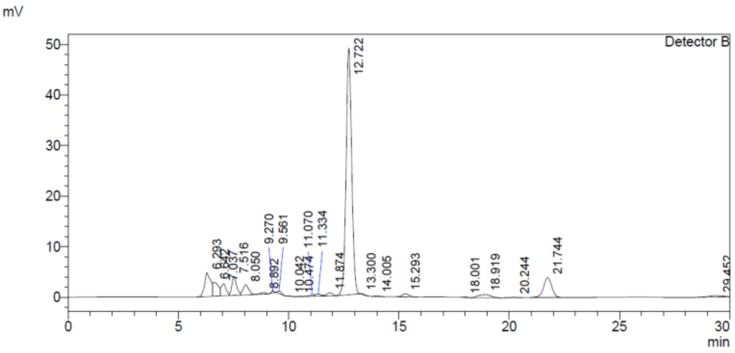

Detector B

Peak#	Ret. Time	Area	Height	Conc.
1	6.293	90260	4716	6.799
2	6.642	47905	2737	3.609
3	7.037	37353	2428	2.814
4	7.516	56625	3732	4.265
5	8.050	36169	1979	2.725
6	8.892	6133	369	0.462
7	9.270	3943	384	0.297
8	9.561	5993	486	0.451
9	10.042	248	30	0.019
10	10.474	604	59	0.046
11	11.070	4157	305	0.313
12	11.334	8166	457	0.615
13	11.874	11857	599	0.893
14	12.722	863231	48748	65.026
15	13.300	1669	141	0.126
16	14.005	1613	110	0.122
17	15.293	12002	615	0.904
18	18.001	1239	80	0.093
19	18.919	25130	669	1.893
20	20.244	1957	78	0.147
21	21.744	100742	3933	7.589
22	29.452	10515	221	0.792
Total		1327510	72875	

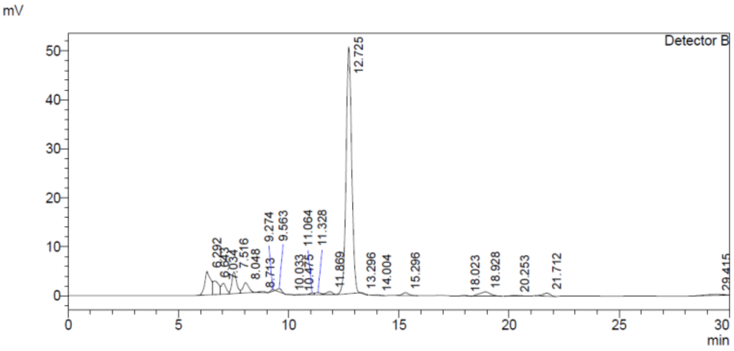

Detector B

Peak#	Ret. Time	Area	Height	Conc.
1	6.292	90694	4804	7.030
2	6.643	50273	2792	3.897
3	7.034	34069	2230	2.641
4	7.516	66742	4412	5.173
5	8.048	38078	2081	2.952
6	8.713	4655	215	0.361
7	9.274	3828	360	0.297
8	9.563	8785	670	0.681
9	10.033	407	44	0.032
10	10.475	678	65	0.053
11	11.064	6146	413	0.476
12	11.328	7953	456	0.616
13	11.869	11883	587	0.921
14	12.725	891718	50304	69.121
15	13.296	1637	123	0.127
16	14.004	1288	90	0.100
17	15.296	11636	602	0.902
18	18.023	1453	94	0.113
19	18.928	30301	872	2.349
20	20.253	2477	94	0.192
21	21.712	13601	602	1.054
22	29.415	11788	239	0.914
Total		1290089	72149	

36 h

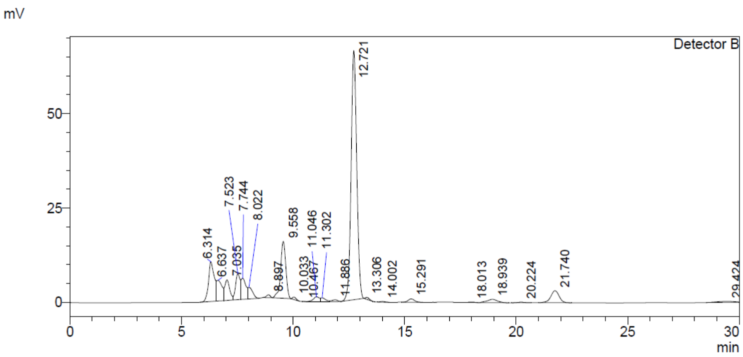

Detector B

Peak#	Ret. Time	Area	Height	Conc.
1	6.314	190099	10562	8.592
2	6.637	91439	5504	4.133
3	7.035	85504	5435	3.865
4	7.523	98436	6870	4.449
5	7.744	83443	5602	3.772
6	8.022	44578	3049	2.015
7	8.897	8814	760	0.398
8	9.558	240246	15056	10.859
9	10.033	7061	639	0.319
10	10.467	112	20	0.005
11	11.046	23649	1277	1.069
12	11.302	15277	959	0.690
13	11.886	7979	462	0.361
14	12.721	1164573	65954	52.638
15	13.306	6105	506	0.276
16	14.002	2728	184	0.123
17	15.291	17884	914	0.808
18	18.013	1687	102	0.076
19	18.939	28982	906	1.310
20	20.224	2957	116	0.134
21	21.740	80277	3165	3.628
22	29.424	10585	220	0.478
Total		2212412	128265	

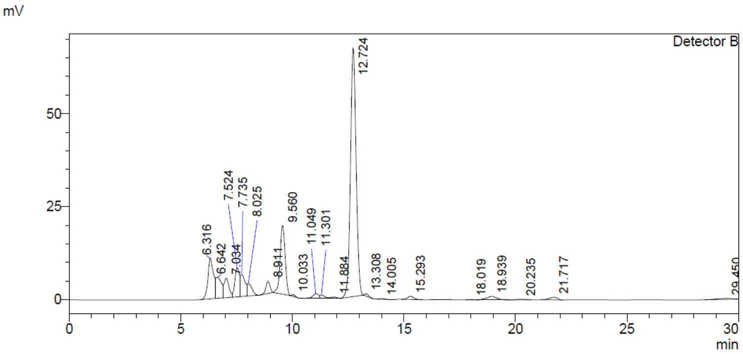

Detector B

Peak#	Ret. Time	Area	Height	Conc.
1	6.316	193140	10926	8.535
2	6.642	97253	5678	4.298
3	7.034	81277	5244	3.592
4	7.524	114508	7845	5.060
5	7.735	83000	5855	3.668
6	8.025	49621	3248	2.193
7	8.911	42816	3279	1.892
8	9.560	283982	18454	12.550
9	10.033	4981	476	0.220
10	11.049	23601	1274	1.043
11	11.301	14091	897	0.623
12	11.884	7884	427	0.348
13	12.724	1176998	66837	52.014
14	13.308	7447	599	0.329
15	14.005	2510	172	0.111
16	15.293	16845	868	0.744
17	18.019	1626	100	0.072
18	18.939	30575	958	1.351
19	20.235	3728	141	0.165
20	21.717	16269	713	0.719
21	29.450	10694	224	0.473
Total		2262845	134214	

42 h

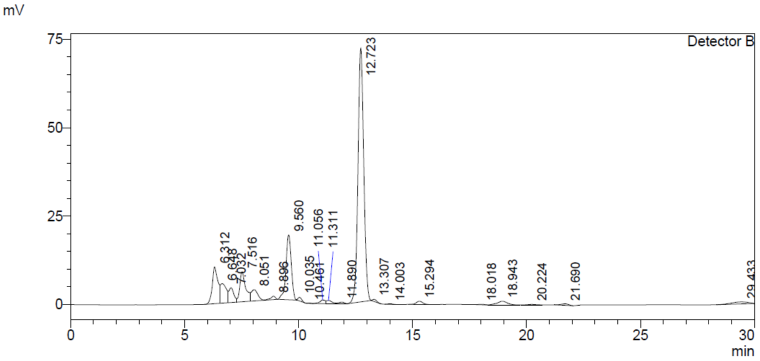

Detector B

Peak#	Ret. Time	Area	Height	Conc.
1	6.312	181049	10411	7.894
2	6.648	101346	5540	4.419
3	7.032	63839	4200	2.784
4	7.516	151481	8472	6.605
5	8.051	65737	3202	2.866
6	8.896	14454	963	0.630
7	9.560	292508	18319	12.754
8	10.035	11340	1023	0.494
9	10.461	803	94	0.035
10	11.056	17041	1017	0.743
11	11.311	14749	871	0.643
12	11.890	7464	448	0.325
13	12.723	1266112	71693	55.207
14	13.307	7471	604	0.326
15	14.003	2624	180	0.114
16	15.294	18828	958	0.821
17	18.018	2128	125	0.093
18	18.943	33412	1088	1.457
19	20.224	3561	141	0.155
20	21.690	9378	428	0.409
21	29.433	28066	565	1.224
Total		2293390	130343	

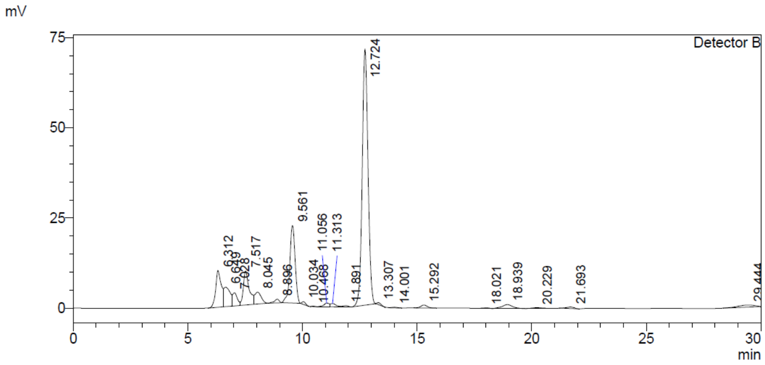

Detector B

Peak#	Ret. Time	Area	Height	Conc.
1	6.312	175241	10189	7.553
2	6.649	101261	5388	4.364
3	7.028	54280	3658	2.339
4	7.517	166245	9396	7.165
5	8.045	65937	3273	2.842
6	8.896	15317	1032	0.660
7	9.561	340091	21486	14.658
8	10.034	7924	746	0.342
9	10.468	1218	135	0.052
10	11.056	17403	1038	0.750
11	11.313	15370	899	0.662
12	11.891	6162	376	0.266
13	12.724	1250346	70925	53.890
14	13.307	6665	552	0.287
15	14.001	2377	162	0.102
16	15.292	16409	841	0.707
17	18.021	1873	112	0.081
18	18.939	33479	1092	1.443
19	20.229	4393	166	0.189
20	21.693	9326	421	0.402
21	29.444	28851	583	1.243
Total		2320166	132471	

48 h

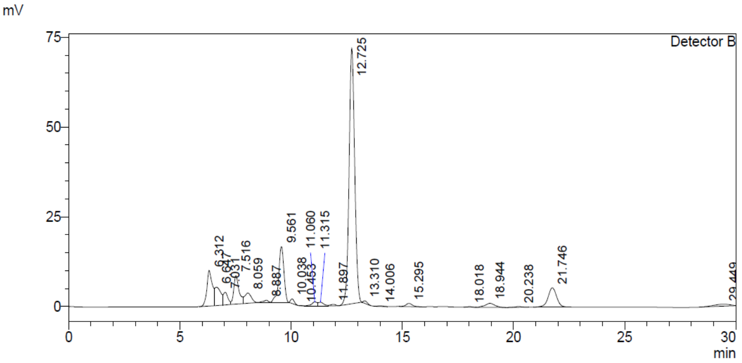

Detector B

Peak#	Ret. Time	Area	Height	Conc.
1	6.312	170103	9905	7.340
2	6.647	94558	5121	4.080
3	7.031	51930	3522	2.241
4	7.516	129502	7923	5.588
5	8.059	60954	2855	2.630
6	8.887	9501	614	0.410
7	9.561	250891	15571	10.827
8	10.038	14801	1280	0.639
9	10.453	345	45	0.015
10	11.060	18818	1109	0.812
11	11.315	16617	984	0.717
12	11.897	6851	420	0.296
13	12.725	1255885	71180	54.194
14	13.310	8592	680	0.371
15	14.006	2525	174	0.109
16	15.295	17920	910	0.773
17	18.018	1963	119	0.085
18	18.944	33805	1128	1.459
19	20.238	3937	154	0.170
20	21.746	140445	5363	6.061
21	29.449	27431	553	1.184
Total		2317375	129609	

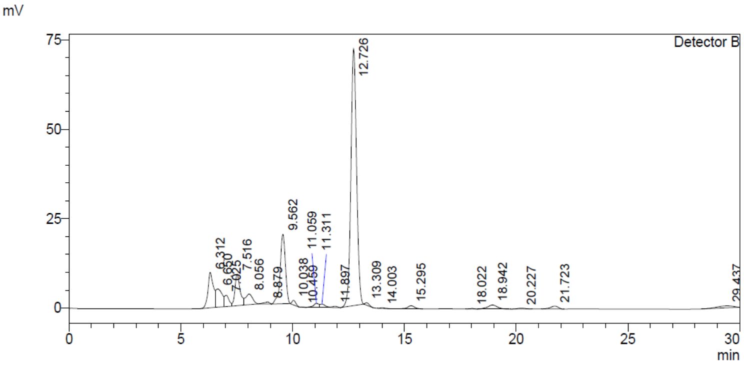

Detector B

Peak#	Ret. Time	Area	Height	Conc.
1	6.312	168700	9920	7.423
2	6.650	97267	5132	4.280
3	7.025	45640	3162	2.008
4	7.516	141845	8814	6.242
5	8.056	64967	2990	2.859
6	8.879	9423	561	0.415
7	9.562	308567	19415	13.578
8	10.038	14955	1275	0.658
9	10.459	515	66	0.023
10	11.059	19901	1149	0.876
11	11.311	15474	931	0.681
12	11.897	5694	360	0.251
13	12.726	1264112	71769	55.625
14	13.309	8376	673	0.369
15	14.003	2270	157	0.100
16	15.295	16324	839	0.718
17	18.022	1929	114	0.085
18	18.942	34708	1153	1.527
19	20.227	4889	188	0.215
20	21.723	18639	802	0.820
21	29.437	28360	569	1.248
Total		2272558	130041	

60 h

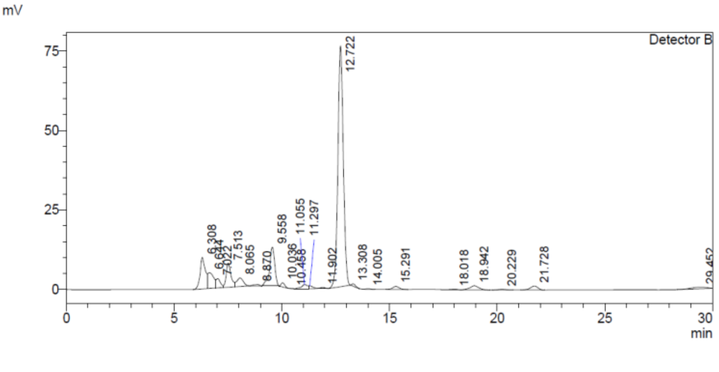

Detector B

Peak#	Ret. Time	Area	Height	Conc.
1	6.308	166647	9890	7.534
2	6.644	94798	4988	4.286
3	7.022	41291	2902	1.867
4	7.513	117759	7542	5.324
5	8.065	57330	2686	2.592
6	8.870	9440	518	0.427
7	9.558	201077	12190	9.091
8	10.036	14967	1270	0.677
9	10.458	196	31	0.009
10	11.055	25319	1347	1.145
11	11.297	12500	876	0.565
12	11.902	6259	409	0.283
13	12.722	1333658	75698	60.297
14	13.308	9191	739	0.416
15	14.005	2450	171	0.111
16	15.291	18718	948	0.846
17	18.018	2227	132	0.101
18	18.942	38037	1300	1.720
19	20.229	4857	190	0.220
20	21.728	29330	1239	1.326
21	29.452	25752	525	1.164
Total		2211804	125592	

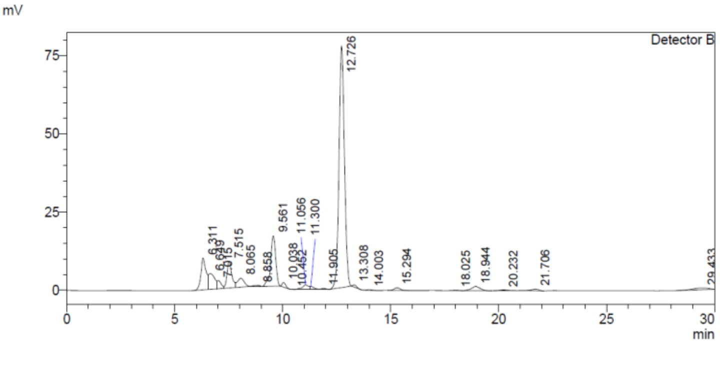

Detector B

Peak#	Ret. Time	Area	Height	Conc.
1	6.311	168747	10186	7.314
2	6.649	101732	5083	4.410
3	7.015	33929	2636	1.471
4	7.515	132091	8474	5.726
5	8.065	61537	2839	2.667
6	8.858	8070	430	0.350
7	9.561	260895	16150	11.309
8	10.038	17480	1493	0.758
9	10.452	194	30	0.008
10	11.056	28845	1481	1.250
11	11.300	13071	937	0.567
12	11.905	5301	356	0.230
13	12.726	1359413	77181	58.924
14	13.308	8533	713	0.370
15	14.003	2229	158	0.097
16	15.294	17134	875	0.743
17	18.025	2296	132	0.100
18	18.944	40036	1368	1.735
19	20.232	5610	217	0.243
20	21.706	11905	524	0.516
21	29.433	28006	558	1.214
Total		2307055	131821	

66 h

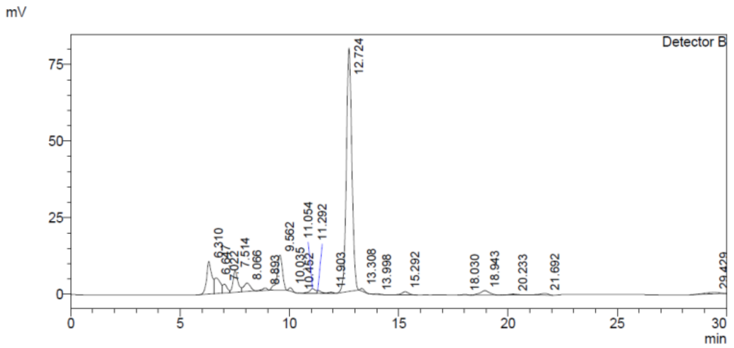

Detector B

Peak#	Ret. Time	Area	Height	Conc.
1	6.310	175507	10665	7.765
2	6.647	98259	5108	4.347
3	7.022	39777	2857	1.760
4	7.514	110659	7011	4.896
5	8.066	58261	2755	2.578
6	8.893	11630	780	0.515
7	9.562	191241	11453	8.461
8	10.035	13047	1146	0.577
9	10.452	285	40	0.013
10	11.054	28847	1490	1.276
11	11.292	11731	886	0.519
12	11.903	6428	423	0.284
13	12.724	1395936	79275	61.760
14	13.308	10387	831	0.460
15	13.998	2446	170	0.108
16	15.292	19677	1000	0.871
17	18.030	2596	149	0.115
18	18.943	40600	1405	1.796
19	20.233	5101	199	0.226
20	21.692	10821	471	0.479
21	29.429	27031	545	1.196
Total		2260268	128659	

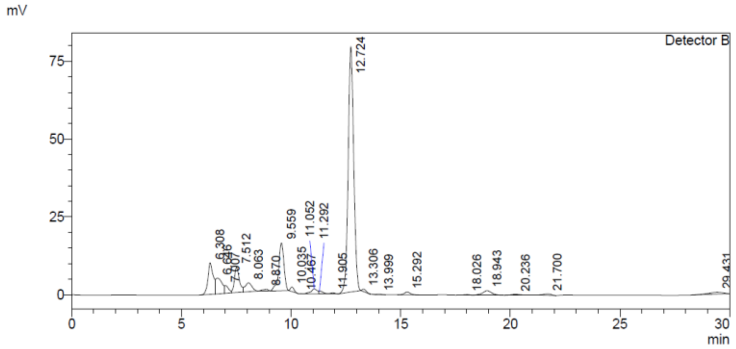

Detector B

Peak#	Ret. Time	Area	Height	Conc.
1	6.308	169349	10163	7.297
2	6.646	102354	5087	4.410
3	7.007	30558	2491	1.317
4	7.512	127107	8142	5.477
5	8.063	60644	2831	2.613
6	8.870	8921	499	0.384
7	9.559	249927	15313	10.769
8	10.035	16565	1428	0.714
9	10.467	171	28	0.007
10	11.052	32299	1589	1.392
11	11.292	11828	935	0.510
12	11.905	5110	349	0.220
13	12.724	1386759	78681	59.751
14	13.306	9234	734	0.398
15	13.999	2240	157	0.097
16	15.292	17829	907	0.768
17	18.026	2531	145	0.109
18	18.943	42587	1468	1.835
19	20.236	6074	230	0.262
20	21.700	9978	446	0.430
21	29.431	28831	573	1.242
Total		2320895	132196	

72 h

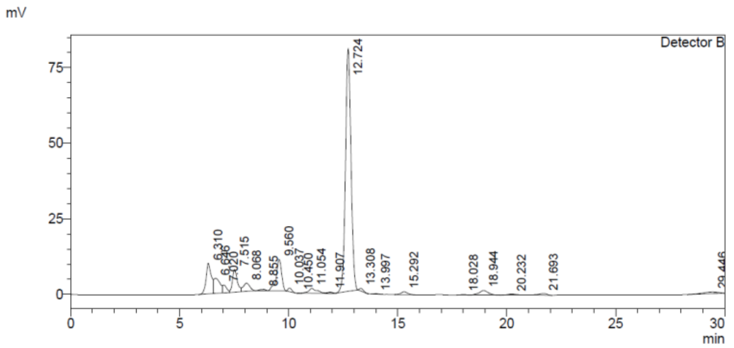

Detector B

Peak#	Ret. Time	Area	Height	Conc.
1	6.310	169121	10151	7.494
2	6.646	97728	5016	4.331
3	7.020	35215	2621	1.560
4	7.515	115704	7505	5.127
5	8.068	56638	2689	2.510
6	8.855	9078	456	0.402
7	9.560	177191	10405	7.852
8	10.037	14486	1217	0.642
9	10.450	227	33	0.010
10	11.054	42006	1584	1.861
11	11.907	6117	413	0.271
12	12.724	1411919	80180	62.566
13	13.308	10791	848	0.478
14	13.997	2430	169	0.108
15	15.292	19020	969	0.843
16	18.028	2440	140	0.108
17	18.944	42510	1474	1.884
18	20.232	5903	225	0.262
19	21.693	10643	474	0.472
20	29.446	27506	555	1.219
Total		2256674	127125	

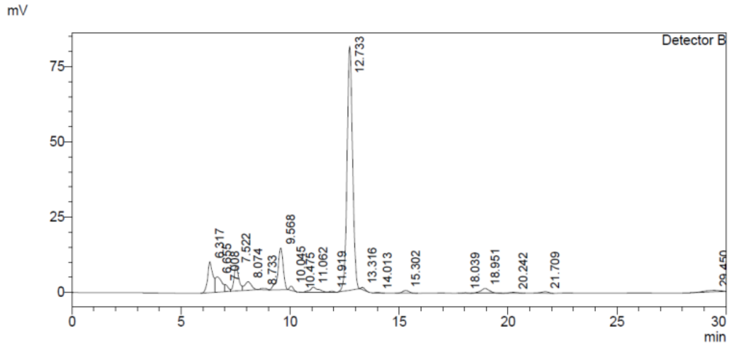

Detector B

Peak#	Ret. Time	Area	Height	Conc.
1	6.317	169276	10221	7.242
2	6.655	102733	5055	4.395
3	7.008	27514	2360	1.177
4	7.522	127299	8260	5.446
5	8.074	59331	2784	2.538
6	8.733	8566	418	0.366
7	9.568	229237	13837	9.808
8	10.045	16693	1418	0.714
9	10.475	140	25	0.006
10	11.062	46528	1719	1.991
11	11.919	5022	353	0.215
12	12.733	1424748	80895	60.957
13	13.316	9132	772	0.391
14	14.013	2291	161	0.098
15	15.302	17624	893	0.754
16	18.039	2382	138	0.102
17	18.951	44462	1548	1.902
18	20.242	6352	243	0.272
19	21.709	10536	468	0.451
20	29.450	27440	555	1.174
Total		2337305	132121	

84 h

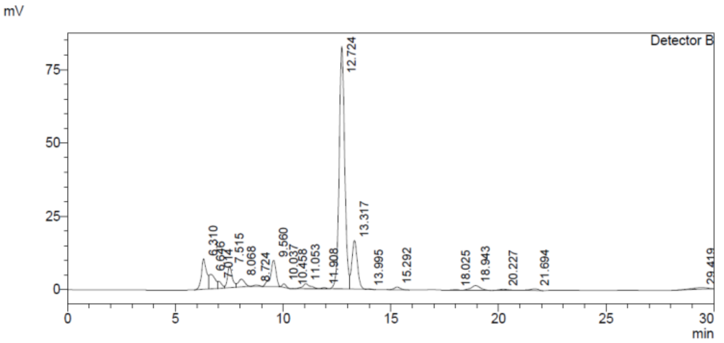

Detector B

Peak#	Ret. Time	Area	Height	Conc.
1	6.310	170853	10388	6.599
2	6.646	97097	4932	3.750
3	7.014	30927	2377	1.195
4	7.515	111841	7289	4.320
5	8.068	55243	2653	2.134
6	8.724	8682	447	0.335
7	9.560	156735	8978	6.054
8	10.037	14209	1189	0.549
9	10.458	309	44	0.012
10	11.053	43796	1740	1.692
11	11.908	5851	408	0.226
12	12.724	1480308	82511	57.178
13	13.317	297731	16583	11.500
14	13.995	1735	135	0.067
15	15.292	19120	969	0.739
16	18.025	2304	134	0.089
17	18.943	45890	1603	1.773
18	20.227	6437	246	0.249
19	21.694	10621	478	0.410
20	29.419	29275	579	1.131
Total		2588964	143683	

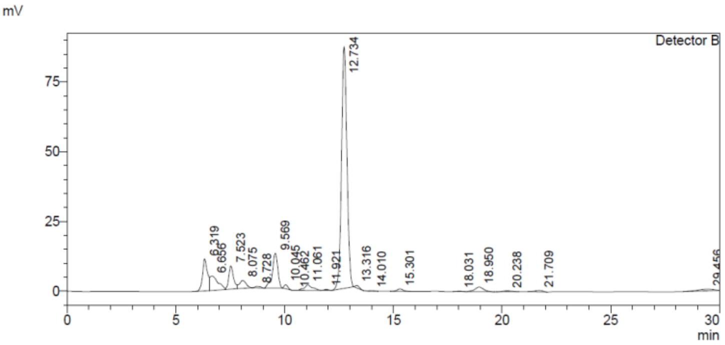

Detector B

Peak#	Ret. Time	Area	Height	Conc.
1	6.319	183403	11466	7.502
2	6.656	131105	5169	5.363
3	7.523	128284	8338	5.248
4	8.075	59891	2849	2.450
5	8.728	9455	499	0.387
6	9.569	208286	12399	8.520
7	10.045	16763	1412	0.686
8	10.462	157	26	0.006
9	11.061	51936	1990	2.124
10	11.921	4842	355	0.198
11	12.734	1523499	86526	62.320
12	13.316	11111	902	0.455
13	14.010	2209	158	0.090
14	15.301	17451	885	0.714
15	18.031	2171	128	0.089
16	18.950	48281	1694	1.975
17	20.238	7221	278	0.295
18	21.709	10769	475	0.441
19	29.456	27816	562	1.138
Total		2444652	136113	

Figure S4 Standard curves

1. Lactic acid (12.68 min) and acetic acid (15.29 min)

   0.02 g/L

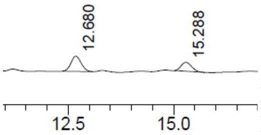

Detector B

Peak#	Ret. Time	Area	Height
1	0.582	98281	3750
2	6.315	24697	2408
3	12.680	3273	197
4	15.288	1993	115

   0.05 g/L

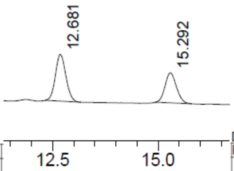

Detector B

Peak#	Ret. Time	Area	Height
1	1.532	91537	2912
2	6.317	22109	2150
3	12.681	8268	479
4	15.292	5818	309

0.101 g/L

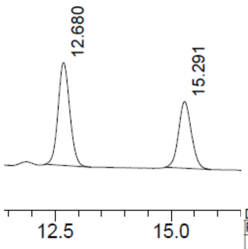

Detector B

Peak#	Ret. Time	Area	Height
1	1.554	80690	2995
2	6.318	18548	1812
3	12.680	17260	987
4	15.291	12340	640

0.202 g/L

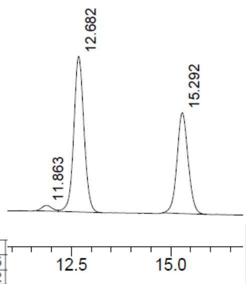

Detector B

Peak#	Ret. Time	Area	Height
1	1.547	92990	3445
2	6.325	11672	1139
3	11.863	1222	69
4	12.682	35195	1976
5	15.292	25036	1281

0.505 g/L

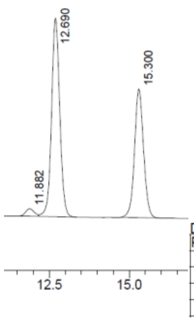

Detector B

Peak#	Ret. Time	Area	Height
1	1.554	91338	3390
2	11.882	3607	190
3	12.690	89481	5011
4	15.300	63961	3247

1.01 g/L

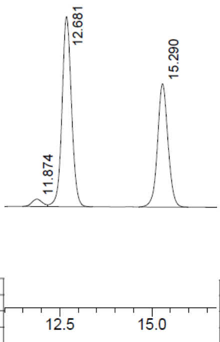

Detector B

Peak#	Ret. Time	Area	Height
1	1.563	83540	3101
2	11.874	7588	389
3	12.681	179666	10055
4	15.290	128942	6521

2.02 g/L

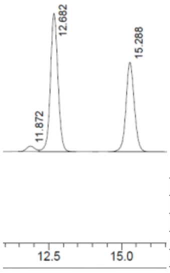

Detector B

Peak#	Ret. Time	Area	Height
1	1.547	76876	2849
2	11.872	15256	776
3	12.682	358454	20038
4	15.288	257402	12975

2. Formic acid

0.02 g/L

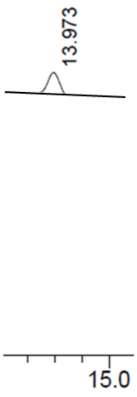

Detector B

Peak#	Ret. Time	Area
1	6.310	26160
2	13.973	12975

0.05 g/L

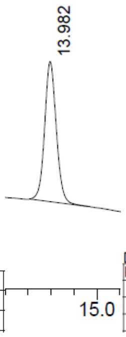

Detector B

Peak#	Ret. Time	Area	Height
1	1.923	71490	496
2	6.313	21111	2114
3	13.982	29621	1686

0.1 g/L

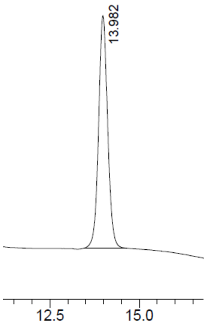

Detector B

Peak#	Ret. Time	Area	Height
1	6.314	16675	1676
2	13.982	59769	3385

0.2 g/L

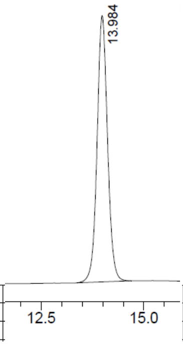

Detector B

Peak#	Ret. Time	Area	Height
1	6.321	10178	948
2	13.984	117902	6660

0.5 g/L

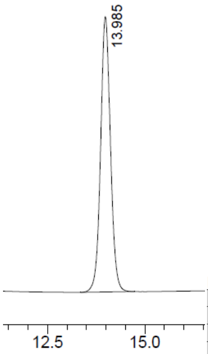

Detector B

Peak#	Ret. Time	Area	Height
1	0.447	37610	1688
2	6.573	10880	510
3	13.985	291259	16483

1 g/L

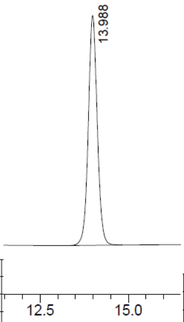

Detector B

Peak#	Ret. Time	Area	Height
1	6.582	35684	1575
2	13.988	580263	32805

2 g/L

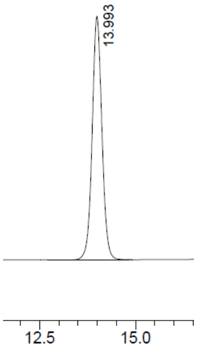

Detector B

Peak#	Ret. Time	Area	Height
1	6.587	81662	3400
2	9.528	2912	203
3	13.993	1148894	64898

3. Glucose (8.9 min) and xylose (9.5 min)

0.02 g/L

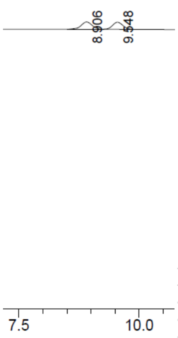

Detector B

Peak#	Ret. Time	Area	Height
1	8.906	5214	335
2	9.548	4846	323
Total		10060	658

0.05 g/L

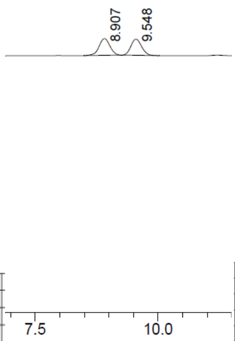

Detector B

Peak#	Ret. Time	Area	Height
1	3.790	48056	1867
2	8.907	12746	838
3	9.548	12114	806
Total		72916	3511

0.1 g/L

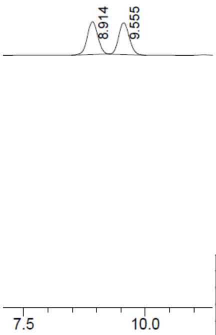

Detector B

Peak#	Ret. Time	Area	Height
1	3.805	51554	2008
2	8.914	25244	1682
3	9.555	24358	1621
Total		101155	5311

0.2 g/L

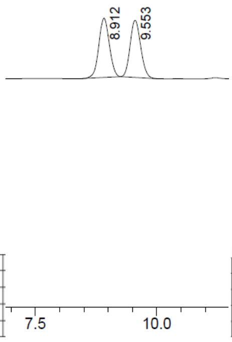

Detector B

Peak#	Ret. Time	Area	Height
1	3.811	50617	1968
2	8.912	50475	3365
3	9.553	48815	3245
Total		149907	8579

0.5 g/L

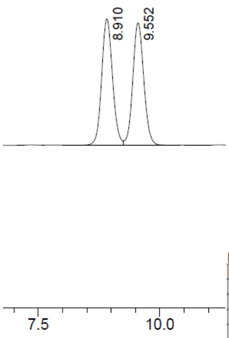

Detector B

Peak#	Ret. Time	Area	Height
1	3.810	50988	1989
2	8.910	151122	10184
3	9.552	147346	9816
Total		349456	21989

1 g/L

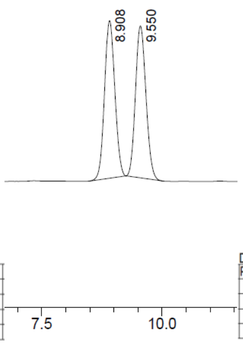

Detector B

Peak#	Ret. Time	Area	Height
1	3.815	51245	2001
2	8.908	254100	17178
3	9.550	248009	16553
Total		553354	35732

2 g/L

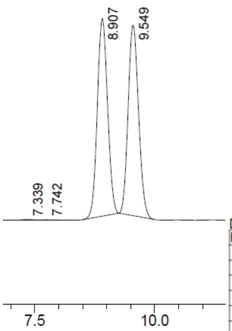

Detector B

Peak#	Ret. Time	Area	Height
1	3.805	50907	1982
2	7.339	1965	121
3	7.742	1085	59
4	8.907	509951	34520
5	9.549	498222	33252
Total		1062130	69934

5 g/L

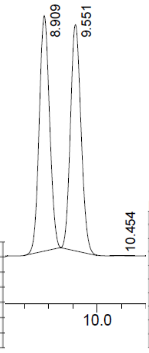

Detector B

Peak#	Ret. Time	Area	Height
1	3.807	50513	1972
2	6.788	348334	9008
3	7.340	201472	5659
4	7.744	65693	2862
5	8.909	1282274	87084
6	9.551	1255533	83661
7	10.454	1019	76
8	11.196	1037	73
Total		3205876	190394
